# Isolation and Cytotoxic Activity of Phyllocladanes from the Roots of *Acacia schaffneri* (Leguminosae)

**DOI:** 10.3390/molecules25173944

**Published:** 2020-08-28

**Authors:** José de Jesús Manríquez-Torres, Marco Antonio Hernández-Lepe, José Román Chávez-Méndez, Susana González-Reyes, Idanya Rubí Serafín-Higuera, Genaro Rodríguez-Uribe, Jesús Martín Torres-Valencia

**Affiliations:** 1 Medical and Psychology School, Autonomous University of Baja California, Universidad 14418, Parque Internacional Industrial Tijuana, Tijuana 22390, Mexico; marco.antonio.hernandez.lepe@uabc.edu.mx (M.A.H.-L.); roman.chavez@uabc.edu.mx (J.R.C.-M.); susana.gonzalez.reyes@uabc.edu.mx (S.G.-R.); serafin.idanya@uabc.edu.mx (I.R.S.-H.); genaro.rodriguez@uabc.edu.mx (G.R.-U.); 2 Department of Chemistry, Autonomous University of Hidalgo, Km 4.5 Carretera Pachuca-Tulancingo, Mineral de la Reforma 42184, Mexico; jmartin@uaeh.edu.mx

**Keywords:** phyllocladanes, cytotoxicity, G0/G1 cell cycle arrest, migration

## Abstract

In research on natural molecules with cytotoxic activity that can be used for the development of new anticancer agents, the cytotoxic activity of hexane, chloroform, and methanol extracts from the roots of *Acacia schaffneri* against colon, lung, and skin cancer cell lines was explored. The hexane extract showed the best activity with an average IC_50_ of 10.6 µg mL^−1^. From this extract, three diterpenoids, phyllocladan-16α,19-diol (**1**), phyllocladan-16α-ol (**2**), and phylloclad-16-en-3-ol (**3**), were isolated and characterized by their physical and spectroscopic properties. Diterpenoids **1** and **2** were tested against the same cancer cell lines, as well as their healthy counterparts, CCD841 CoN, MRC5, and VH10, respectively. Compound **1** showed moderate activity (IC_50_ values between 24 and 70 μg mL^−1^), although it showed a selective effect against cancer cell lines. Compound **2** was practically inactive. The cytotoxicity mechanism of **1** was analyzed by cell cycle, indicating that the compound induces G0/G1 cell cycle arrest. This effect might be generated by DNA alkylation damage. In addition, compound **1** decreased migration of HT29 cells.

## 1. Introduction

Cancer is a major public health problem worldwide and it is one of the main causes of death [1]. This type of chronic non-communicable diseases is among the most prominent human diseases. It has become a major health problem in past decades and is now the second leading cause of death globally. Although there are different types of treatment, such as chemotherapy, immune therapy, radiation, hormone therapy, and targeted therapy used against cancer, they have possible side effects and significant deficiencies, which has stimulated scientific interest in the discovery of newer anticancer, anti-tumor, and anti-inflammatory agents from natural sources [2,3].

Approximately 60% of drugs currently used for cancer treatment have been isolated from natural products [4] with plants being the most important source of these compounds with biological activity [5]. Studies from all over the world indicate that patients with cancer commonly resort to alternative therapies as a simultaneous resource to find a cure, particularly in these medicinal plants [6]. In different studies it is mentioned that more than 3000 plants with anti-cancer properties are acknowledged around the world [7]. As stated above, in urban areas of Mexico, more than 30% of patients diagnosed with cancer use plant extracts as an alternative or complementary therapy. Hence, this demonstrates the important role of complementary and alternative medicine in the society and culture of the Mexican population [6,7,8].

*Acacia schaffneri* (Leguminosae) is abundant in the south-central valleys of Mexico, where it has been used traditionally for the treatment of symptoms associated with cancer and inflammation. A decoction of the bark is employed to treat gastric ulcers and skin. This species is a source of *seco*-oxacassanes, which are rare diterpenes, and whose isolation, characterization [9], and cytotoxic activity were described [10].

**Figure 1:**
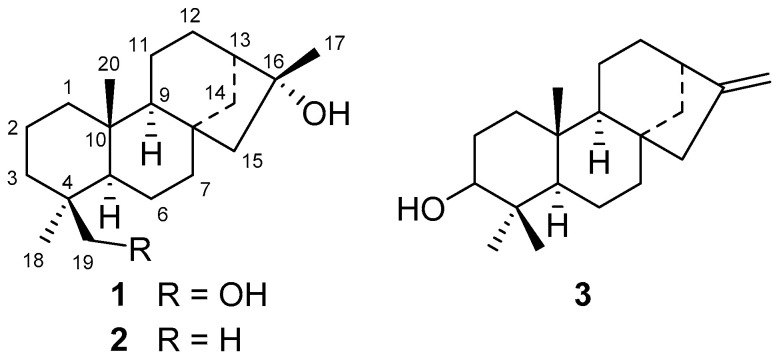
Chemical structures of phyllocladanes **1**–**3** from *Acacia schaffneri*.

The present manuscript describes the isolation and characterization of phyllocladanes **1**–**3** (Figure 1) isolated from the hexane extract of the roots of *A. schaffneri*. This is the first time that this type of diterpene is reported in *Acacia*. Additionally, the anti-proliferative activity of hexane, chloroform, and methanol extracts from the roots of *A. schaffneri*, and of phyllocladanes, was explored. A preliminary study of the mechanism of cytotoxic activity exhibited by diterpene **1** cell cycle analysis and inhibition of migration capacity was also realized.

## 2. Results

### 2.1. Extraction and Isolation

**Figure 2:**
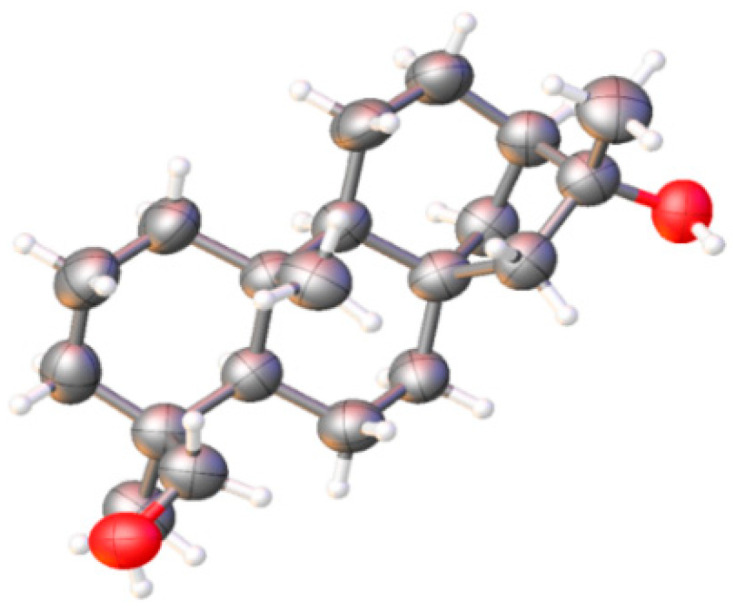
X-ray diffraction perspective of phyllocladan-16α, 19-diol (**1**).

The hexane extract from the roots of *A. schaffneri* led to a precipitate, which was filtered to obtain a yellow powder that was labeled (IP.R), while the soluble part was concentrated and named as (SP.R). Subsequently, these parts were subjected to separation by column chromatography and TLC preparative plates. Chromatographic separation of IP.R in a preparative plate of 10 × 20 cm using CHCl_3_-AcOEt (4:1) led to obtaining four main compounds, which were analyzed by ^1^H NMR. Three of them were previously reported as the aerial part of *A. schaffneri* [9], while the new diterpenoid phyllocladan-16α,19-diol (**1**) was obtained in a 30% yield, the molecular formula of which was established as C_20_H_34_O_2_ by GS-MS, and its relative configuration was determined by X-ray diffraction analysis (Figure 2). The crystallographic data is found in Supplementary Materials. Compound **1** showed [α]_D_ = −7, and in its ^1^H NMR spectrum the methylene protons at C-19 were observed as an AB system at δ 3.75 and 3.41 (*J*_19,19′_ = 10.9 Hz). The signals for the tertiary methyls of the structure were seen at δ 1.33 (3H, s), δ 0.96 (3H, s), and 0.85 (3H, s).

**Figure 3:**
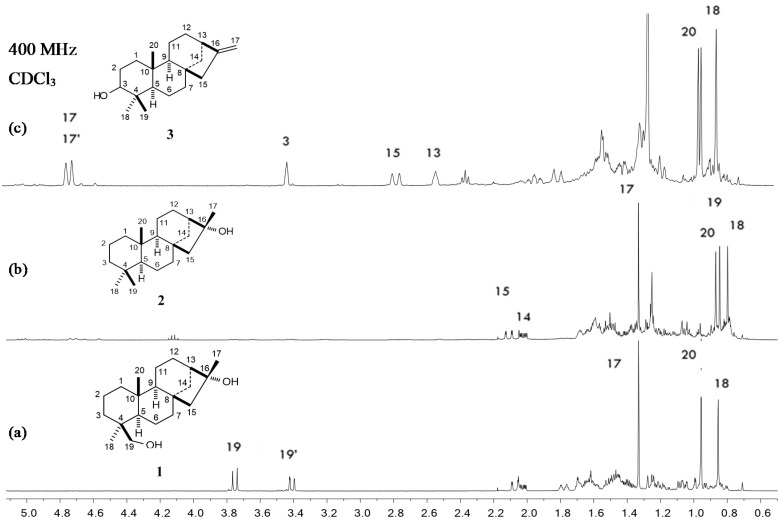
^1^H NMR spectra of phyllocladanes: (**a**) phyllocladan-16α,19-diol (**1**), (**b**) phyllocladan-16α-ol (**2**), and phylloclad-16-en-3-ol (**3**) (**c**).

Isolation and characterization of metabolites contained in the soluble part (SP.R) of the hexane extract were carried out on a column using silica gel and hexane, hexane-CHCl_3_, CHCl_3_, and CHCl_3_ as eluents to obtain four coarse fractions (F1–F4), which were analyzed by TLC and NMR. From fraction 1 (F1), only fatty material was identified by NMR. Recomatography of fraction 2 (F2), using hexane-CHCl_3_ mixtures in increasing polarity, showed the presence of two metabolites, phyllocladan-16α-ol (**2**) and traces of a diterpene identified as phylloclad-16-en-3-ol (3) (Figure 3). The isolated compounds were assigned by comparison with phyllocladan-16α,19-diol (**1**) and described data [11,12,13] (Table 1).

Phyllocladan-16α,19-diol (1) were further evaluated by single-crystal X-ray diffraction studies which provided independent structural proof and evidenced some stereochemical details. The relevant crystal data, showing that XRD structure determinations could be performed in all cases, can be inspected in supplementary material.

### 2.2. Cytotoxic Activity

Of the isolated secondary metabolites, only two (compound **1** and **2**) could be obtained in sufficient quantities to evaluate their cytotoxic activity against HT-29, A-549, and UACC-62 cancer cell lines with the SRB assay. Their cytotoxic activity was compared with the activity against their counterpart non-carcinogenic cells, CCD-841 CoN, MRC-5, and VH-10, correspondingly. 5-FU was used as a positive control, and the results are shown in Table 2.

### 2.3. Cell Cycle Analysis

Flow cytometry was used to evaluate if the mechanism by which **1** produced cytotoxicity was related to the arrest of cell cycle progression in HT-29 and A-549 cells. The results are shown in Table 3. Furthermore, **1** was tested using the IC_50_ obtained in the SRB assay as a base.

### 2.4. Wound Migration Assay

HT-29 cells were incubated for 24 h in either the absence or the presence of 1 for wound migration assay, in which 50 μg mL^−1^ 5-FU was used as a positive control. Results show moderate suppressed cell migrations to the wounded area in a concentration dependent manner, by 40% to 60% at 15 μg mL^−1^ of 1.

## 3. Discussion

In previous works, we reported the isolation and the cytotoxic activity of *seco*-oxacassanes isolated from the aerial parts of *A. schaffneri* [9,10]. These compounds showed relevant cytotoxic activity [10], but they were not selective against the non-malignant cell lines tested. In this paper, we explored the chemical composition of the roots of this species. According to the National Cancer Institute (NCI), plant extracts and pure compounds with cytotoxic ED_50_ (Effective Dose 50) values of ≤30 µg mL^−1^ and ≤4 µg mL^−1^, respectively, are considered active [5]. In the cell lines analyzed (Table 2), it was discovered that the hexane and chloroform extracts of *A. schaffneri* showed IC_50_ values of 10.1 to 10.9 µg mL^−1^ which were candidates for chromatographic separation. This process led to obtaining *seco*-oxacasanes [9] and phyllocladanes **1**–**3**. The cytotoxic activity of **1** and **2** was tested using the SRB assay. Diterpenoid **2** was recognized as inactive (IC_50_ > 100 µg mL^−1^). Although the activity of compound **1** does not meet the criteria established by the NCI (their IC_50_ values range from 24.1 to 33.5 µg mL^−1^), this molecule has selectivity for cancer cells. In some cases, as much as 245% more doses were needed in order to affect normal cells, as can be observed on skin cells UACC‒62 (28.3 µg mL^−1^) and VH‒10 (70.2 µg mL^−1^) (Table 2).

A flow cytometry analysis was performed to assess whether the mechanism by which **1** produced cytotoxicity was related to the cell cycle arrest progression in HT-29 and A-549 cells (Table 3). In control cells, a percentage range (49–51%) was in the G0/G1 phase of the cell cycle, another percentage range (8–12%) in the S phase, and the remaining cells (28–31%) in the G2/M phase. Treated with colchicine, which is known to cause cell cycle arrest by inhibiting mitosis, the percentage of cells in G0/G1 and S significantly decreased, while the percentage of cells in G2/M markedly increased. Diterpene **1** caused an increase in the number of cells in the G0/G1 phase, while those of G2/M decreased. This finding implies that **1** induced cell cycle arrest progression in HT-29 and A-549 cells in the G0/G1 phase at a higher percentage rate (70%) (Table 3). These increases were reached with statistical significance in HT-29 and A-549 cells in a concentration of 60 µg mL^−1^ and 30 µg mL^–1^, respectively.

HT-29 cells were incubated for 24 h in the absence and presence of **1** in a wound-healing assay, in which 50 µg mL^−1^ of 5-FU was used as a positive control. The results showed that diterpene 1 moderately prevents cell migration in the wound area at a concentration of 15 mg (ranging from 40% to 60% of cells). HT-29 cells were selected for this study due to the suitability of their growth characteristics to observe wound healing. Using the wound-healing assay, the results showed that **1** can inhibit migration in HT-29 cells, ascertaining that it has some antimetastatic effect.

## 4. Materials and Methods

### 4.1. General Experimental Procedures

Melting points were determined on a Fisher–Johns melting point apparatus and are uncorrected. Optical rotations were determined in CHCl_3_ on a Perkin–Elmer 341 polarimeter. UV spectra were determined on a Perkin–Elmer Lambda 12 UV/vis spectrophotometer. NMR measurements, including APT and COSY, were performed at 400 MHz for ^1^H and 100 MHz for ^13^C on a JEOL Eclipse 400 spectrometer from CDCl_3_ solutions using TMS as an internal standard. The experiments, including gHMQC and gHSQC, were acquired as additional spectroscopy for the compound phyllocladan-16α, 19-diol (**1**). LR-MS were recorded at 70 eV on a Hewlett-Packard 5890 Series II spectrometer and at 20 eV on a Hewlett-Packard 5989A spectrometer, while HR-MS were measured on an Agilent LCTOF instrument at the UCR Mass Spectrometry Facility, University of California, Riverside. TLC was performed on silica gel 60 (Analtech, layer thickness 0.1 mm, 20 × 20 cm with fluorescent indicator F_254_) precoated glass plates. Column chromatography was carried out on Merck silica gel 60 (Aldrich, 230,400 mesh). Single-crystal X-ray diffraction analysis data were obtained using an Agilent Xcalibur Atlas Gemini diffractometer in the w-2θ scan mode at 298(2) K. Crystals of 1 were measured with graphite-monochromated Mo Kα (λ = 0.71073 Å) radiation. The structures were solved by direct methods using the Sir2004 software. All structure refinements were done by full-matrix least-squares on F2 and the non-hydrogen atoms were treated anisotropically.

### 4.2. Plant Material

Specimens of *A. schaffneri* (S. Watson) F. J. Hermann were collected from the municipality of Zempoala, Hidalgo state, Mexico during March 2019 and identified by Prof. Manuel Gonzalez Ledesma. A voucher specimen (JM Torres Valencia 124) is preserved in the Herbarium of Universidad Autonoma del Estado de Hidalgo, Mineral de la Reforma, Hidalgo, México.

### 4.3. Extraction and Isolation

Air-dried, ground root (1.0 kg) of *A. schaffneri* was extracted three times with *n*-hexane (3 L) for 24 h at room temperature. Filtration and evaporation of the extract afforded a yellow, viscous oil (6 g). The hexane extract (1 g) was separated by column chromatography using chloroform and ethyl acetate in increasing polarity obtaining 50 fractions. Eluates 10–15 were combined to obtain 425 mg, from which a portion (100 mg) was purified by TLC (CHCl_3_-EtOAc, 4:1), affording **1** (18 mg, Rf 0.2), **2** (69 mg, Rf 0.5), and **3** (6 mg, Rf 0.6).

### 4.4. Cell Line and Culture Conditions

Human colon cancer cells HT-29, lung cancer cells A-549, melanoma cells UACC-62, and noncarcinogenic counterpart cells of colon CCD-841 CoN, lung MRC5, and skin VH-10 were procured from the American Type Culture Collection (ATCC, Manassas, VA, USA) and European Collection of Cell Cultures (ECACC). The cells were maintained in DMEM. All cell lines were cultured in a humidified atmosphere containing 5% CO_2_ at 37 °C.

### 4.5. Cytotoxic Activity and Cell Viability Assays

The cytotoxic activity of the extracts and isolated compounds was measured with the SRB assay [14]. In brief, cells in growth medium were seeded into 96- well plates at 1 × 106 cells/well, different concentrations of extracts and pure compounds (diluted in fresh medium) were added, and cells were incubated at 37 °C for 48 h. The cells were fixed with 50%, *v*/*v*, trichloroacetic acid (TCA) for 1 h at 4 °C. After being washed 5 times with distilled water, cells were stained for 30 min with 100 μL of 0.4%, *w*/*v*, SRB in 1%, *v*/*v*, acetic acid, after which the excess dye was removed by washing repeatedly with 1%, *v*/*v*, acetic acid. The protein-bound dye was dissolved in 10 mM Tris base solution for absorbance determination at 492 nm using a microplate reader.

### 4.6. Cell Cycle Analysis

Cell cycle analysis by quantitation of DNA content was performed by flow cytometry. Cells were seeded (1 × 106 cells/well) in 6-well plates and treated with two concentrations of 2, its IC_50_ and twice the IC_50_, using the SRB assay. After 24 h at 37 °C, cells were harvested by trypsinization and centrifuged at 1500 rpm for 5 min at 4 °C. Cell pellets were suspended in 70%, *v*/*v*, ethanol (1 mL), lightly mixed, and kept at 0 °C overnight. Ethanol was removed, RNase (1:100 volumes of 20 mg mL^−1^) was added, and the cells were incubated at 4 °C for 48 h. DNA was stained by adding propidium iodide (50 µg mL^−1^) and keeping the cells for 20–30 min at room temperature. The stained cells were analyzed for DNA histograms and cell cycle phase distribution by flow cytometer (Cytomics FC500-MPL, Beckman Coulter Inc., Indianapolis, IN, USA). Colchicine (0.2 μg mL^−1^) was used as a positive control.

### 4.7. Wound Migration Assay

HT-29 cells were seeded into 6-well plates (1 × 10^6^ cells/mL) and grown to 80–90% confluence for the experiment. After aspirating the medium, cells were scraped with a sterile micropipette tip to create a wound. They were washed twice with PBS to remove cellular debris and then replaced with complete McCoy’s 5A. HT-29 cells were treated with 2 at its IC_50_, and with two times its IC_50_, providing the total variability of the cells under these experimental conditions and incubating for 24 h at 37 °C. Cell migration into the wound area was photographed at the 0 and 24 h stages, respectively, for image analysis of each treatment. The level of cell migration was determined using a Hewlett–Packard scanner and NIH Image software (Image J), and then it was expressed as a percentage of each control for the mean of wound closure area, as described [15].

### 4.8. Statistical Analysis

All in vitro experiments were made in triplicate. Results are expressed as mean ± standard error of the mean (S.E.M). The results were calculated by one-way analysis of variance (ANOVA) followed by Bonferroni’s post hoc test for multiple comparisons and assessed with two-way ANOVA for repeated measures and followed by Student’s *t*-test. A P value of less than 0.05 was considered statistically significant.

## 5. Conclusions

In conclusion, three phyllocadan-type diterpenoids were isolated from the roots of *Acacia schafneri*, from which phyllocladan-16α, 19-diol (**1**) had a selective cytotoxic effect against human colon (HT-29), lung (A-549), and skin (UACC-62) cancer cell lines, compared to their non-malignant counterparts CCD-841 CoN, MRC-5, and VH-10. Furthermore, this compound induces the arrest of the cell cycle in the G0/G1 phase. Although this activity is not as powerful, it can be offset by the selectivity mentioned above.

## Figures and Tables

**Table 1:** ^13^C NMR (100 MHz, CDCl_3_) data of phyllocladanes **1**–**3**.

Position	δ_C_, mult.		
	1	2	3
1	41.9, CH_2_	39.3, CH_2_	33.6, CH_2_
2	20.3, CH_2_	20.3, CH_2_	24.7, CH_2_
3	35.4, CH_2_	42.0, CH_2_	75.9, CH
4	37.7, C	33.1, C	56.2, C
5	57.0, CH	56.9, CH	56.2, CH
6	19.1, CH_2_	18.9, CH_2_	19.6, CH_2_
7	41.9, CH_2_	41.6, CH_2_	40.5, CH_2_
8	44.4, C	44.5, C	43.3, C
9	56.9, CH	56.2, CH	49.1, CH
10	38.4, C	37.8, C	37.3, C
11	18.0, CH_2_	18.3, CH_2_	18.6, CH_2_
12	27.4, CH_2_	27.6, CH_2_	32.0, CH_2_
13	47.5, CH	47.5, CH	42.3, CH
14	48.9, CH_2_	49.0, CH_2_	49.9, CH_2_
15	49.4, CH_2_	49.6, CH_2_	41.1, CH_2_
16	82.1, C	82.2, C	157.3, C
17	23.9, CH_3_	23.9, CH_3_	102.1, CH_2_
18	27.1, CH_3_	21.9, CH_3_	28.2, CH_3_
19	69.6, CH_2_	33.7, CH_3_	22.0, CH_3_
20	15.4, CH_3_	14.8, CH_3_	14.6, CH_3_

qC = quaternary carbon; CH = tertiary carbon; CH_2_ = secondary carbon; CH_3_ = primary carbon.

**Table 2:** Cytotoxic activity (IC_50_) of extracts and phyllocladanes **1** and **2** against several human cell lines. Compounds were tested from 0.1 to 100.0 µg mL^−1^.

Sample	Colon Cells		Lung Cells		Skin Cells	
	HT-29	CCD-841 CoN	A-549	MRC-5	UACC-62	VH-10
Hexane	10.1 ± 0.5	– ^a^	10.9 ± 0.1	– ^a^	10.8 ± 0.8	– ^a^
CHCl_3_	9.9 ± 0.2	– ^a^	13.8 ± 1.7	– ^a^	16.8 ± 0.6	– ^a^
MeOH	>100	– ^a^	>100	– ^a^	>100	– ^a^
**1**	33.5 ± 1.6	41.1 ± 1.9	24.1 ± 1.3	32.5 ± 2.0	28.3 ± 5.5	70.2 ± 16.9
**2**	>100	>100	>100	>100	>100	>100
**5-FU ^b^**	0.8 ± 0.9	– ^a^	3.6 ± 0.3	>130	0.7 ± 0.2	>130

^a^ Not tested; ^b^ 5-FU was tested from 0.013 to 130 μg mL^−1^.

**Table 3:** Flow cytometry of phyllocladan-16α, 19-diol (**1**) against colon and lung cancer cells.

Sample	HT29				A-549			
	Apop.	G1/G0	S	G2/M	Apop.	G1/G0	S	G2/M
**Control**	0.8 ± 0.1	48.9 ± 3.5	12.3 ± 0.8	30.5 ± 3.3	0.2 ± 0.1	58.4 ± 1.0	7.0 ± 1.1	22.6 ± 0.5
**Colchicine**	0.2 ± 0.2	9.0 ± 3.0	4.6 ± 1.2	82.2 ± 1.3	0.4 ± 0.1	5.9 ± 0.9	2.2 ± 0.2	88.0 ± 0.7
**1** (30 µg)	1.8 ± 0.2	59.1 ± 8.0	9.2 ± 2.3	19.8 ± 3.9	0.8 ± 0.1	73.6 ± 2.9	5.5 ± 1.4	14.7 ± 1.8
**1** (60 µg)	3.2 ± 0.3	76.6 ± 3.3	6.1 ± 1.5	9.7 ± 2.2	2.8 ± 0.5	67.8 ± 0.4	4.5 ± 0.9	19.0 ± 2.7
**1** (100 µg)	1.4 ± 0.2	64.2 ± 6.2	5.6 ± 1.1	11.4 ± 2.0	6.7 ± 0.1	53.2 ± 4.7	6.8 ± 0.5	21.5 ± 2.3

## Supplementary Materials

The following are available online, Figure S1: ^1^H-NMR spectrum of phyllocladan-16α,19-diol (**1**); Figure S2: ^13^C-NMR spectrum of phyllocladan-16α,19-diol (**1**); Figure S3: APT experiment spectrum of phyllocladan-16α,19-diol (**1**); Figure S4: ^1^H-^1^H COSY spectrum of phyllocladan-16α,19-diol (**1**); Figure S5: HSQC spectrum of phyllocladan-16α,19-diol (**1**); Figure S6: HSQC spectrum of phyllocladan-16α,19-diol (**1**); Figure S7: ^1^H-NMR spectrum of phyllocladan-16α-ol (**2**); Figure S8: APT experiment spectrum of phyllocladan-16α-ol (**2**); Figure S9: ^1^H-^1^H COSY spectrum of phyllocladan-16α-ol (**2**); Figure S10: ^1^H-NMR spectrum of phylloclad-16-en-3-ol (**3**); Figure S11: APT experiment spectrum of phylloclad-16-en-3-ol (**3**); Figure S12: ^1^H-^1^H COSY spectrum of phylloclad-16-en-3-ol (**3**); Figure S14: HSQC spectrum of phylloclad-16-en-3-ol (**3**).
