# Supplementary material for: Isolation and Cytotoxic Activity of Phyllocladanes from the Roots of Acacia schaffneri (Leguminosae)
Source: Molecules. 2020 Aug 28;25(17):3944. doi: 10.3390/molecules25173944 (PMC7504352; doi:10.3390/molecules25173944)
Supplement: Supplementary file 1 [file molecules-25-03944-s001.pdf]

Isolation and Cytotoxic Activity of Phyllocladanes from the Roots of *Acacia schaffneri* (Leguminosae)

José de Jesús Manríquez-Torres [1,*], Marco Antonio Hernández-Lepe [1], José Román Chávez-Méndez [1], Susana González-Reyes [1], Idanya Rubí Serafín-Higuera [1], Genaro Rodríguez-Uribe [1] and Jesús Martín Torres-Valencia [2]

[1] Medical and Psychology School, Autonomous University of Baja California, Universidad 14418, Parque Internacional Industrial Tijuana, 22390 Tijuana, B.C. México; marco.antonio.hernandez.lepe@uabc.edu.mx (M.A.H.-L.); roman.chavez@uabc.edu.mx (J.R.C.-M.); susana.gonzalez.reyes@uabc.edu.mx (S.G.-R.); serafin.idanya@uabc.edu.mx (I.R.S.-H.); genaro.rodriguez@uabc.edu.mx (G.R.-U.)

[2] Department of Chemistry, Autonomous University of Hidalgo, Km 4.5 Carretera Pachuca-Tulancingo, 42184 Mineral de la Reforma, Hidalgo, México; jmartin@uaeh.edu.mx

* Correspondence: jose.de.jesus.manriquez.torres@uabc.edu.mx; Tel.: +52-664-682-1233

Supplementary data

Figure S1: ^{1}H-NMR spectrum of phyllocladan-16α,19-diol (**1**)

Figure S2: ^{13}C-NMR spectrum of phyllocladan-16α,19-diol (**1**)

Figure S3: APT experiment spectrum of phyllocladan-16α,19-diol (**1**)

Figure S4: ^{1}H-^{1}H COSY spectrum of phyllocladan-16α,19-diol (**1**)

Figure S5: HSQC spectrum of phyllocladan-16α,19-diol (**1**)

Figure S6: HSQC spectrum of phyllocladan-16α,19-diol (**1**)

Figure S7: ^{1}H-NMR spectrum of phyllocladan-16α-ol (**2**)

Figure S8: APT experiment spectrum of phyllocladan-16α-ol (**2**)

Figure S9: ^{1}H-^{1}H COSY spectrum of phyllocladan-16α-ol (**2**)

Figure S10: ^{1}H-NMR spectrum of phylloclad-16-en-3-ol (**3**)

Figure S11: APT experiment spectrum of phylloclad-16-en-3-ol (**3**)

Figure S12: ^{1}H-^{1}H COSY spectrum of phylloclad-16-en-3-ol (**3**)

Figure S14: HSQC spectrum of phylloclad-16-en-3-ol (**3**)

Compound **1** NMR

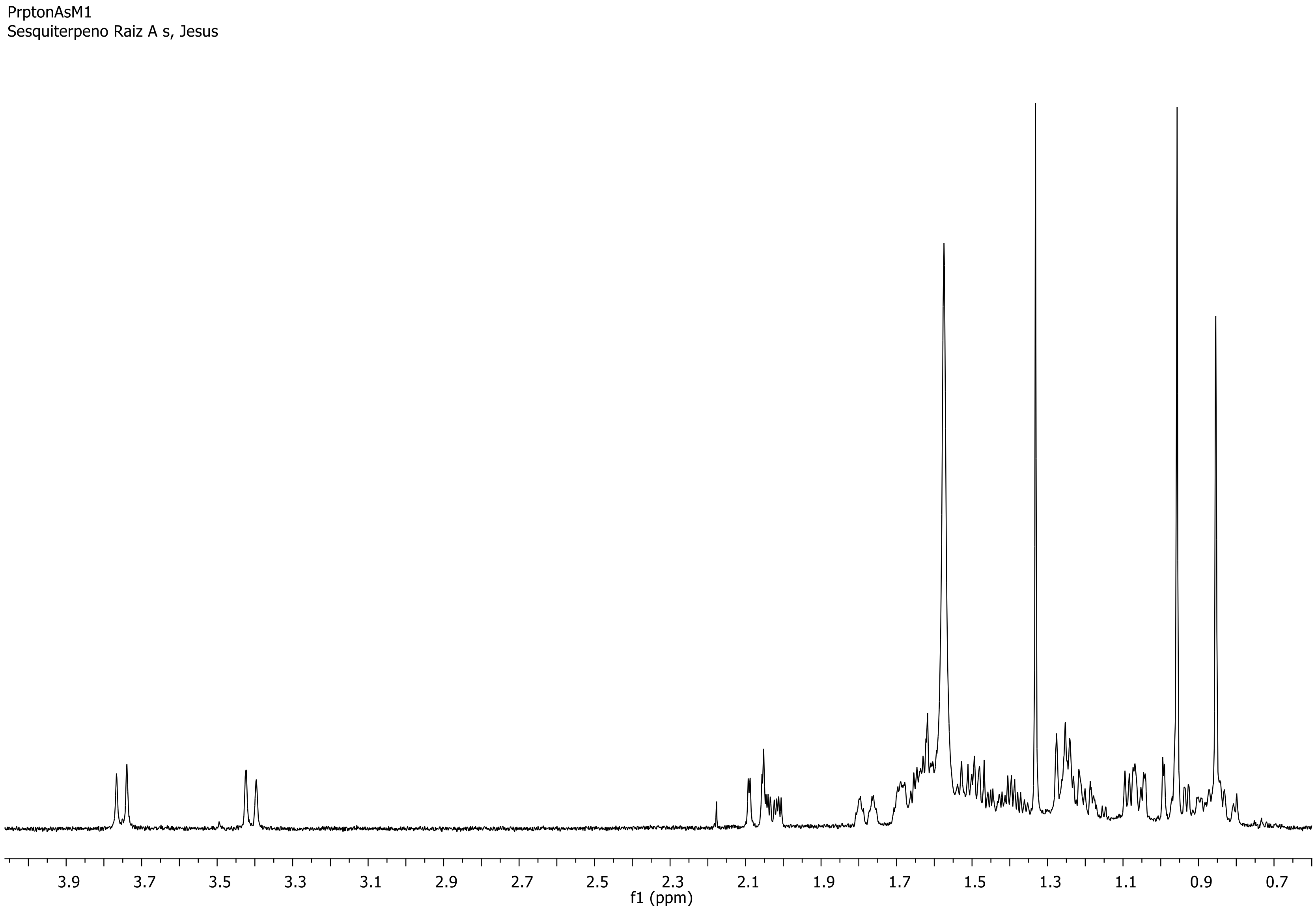

Figure S1: ^{1}H-NMR spectrum of phyllocladan-16α,19-diol (**1**)

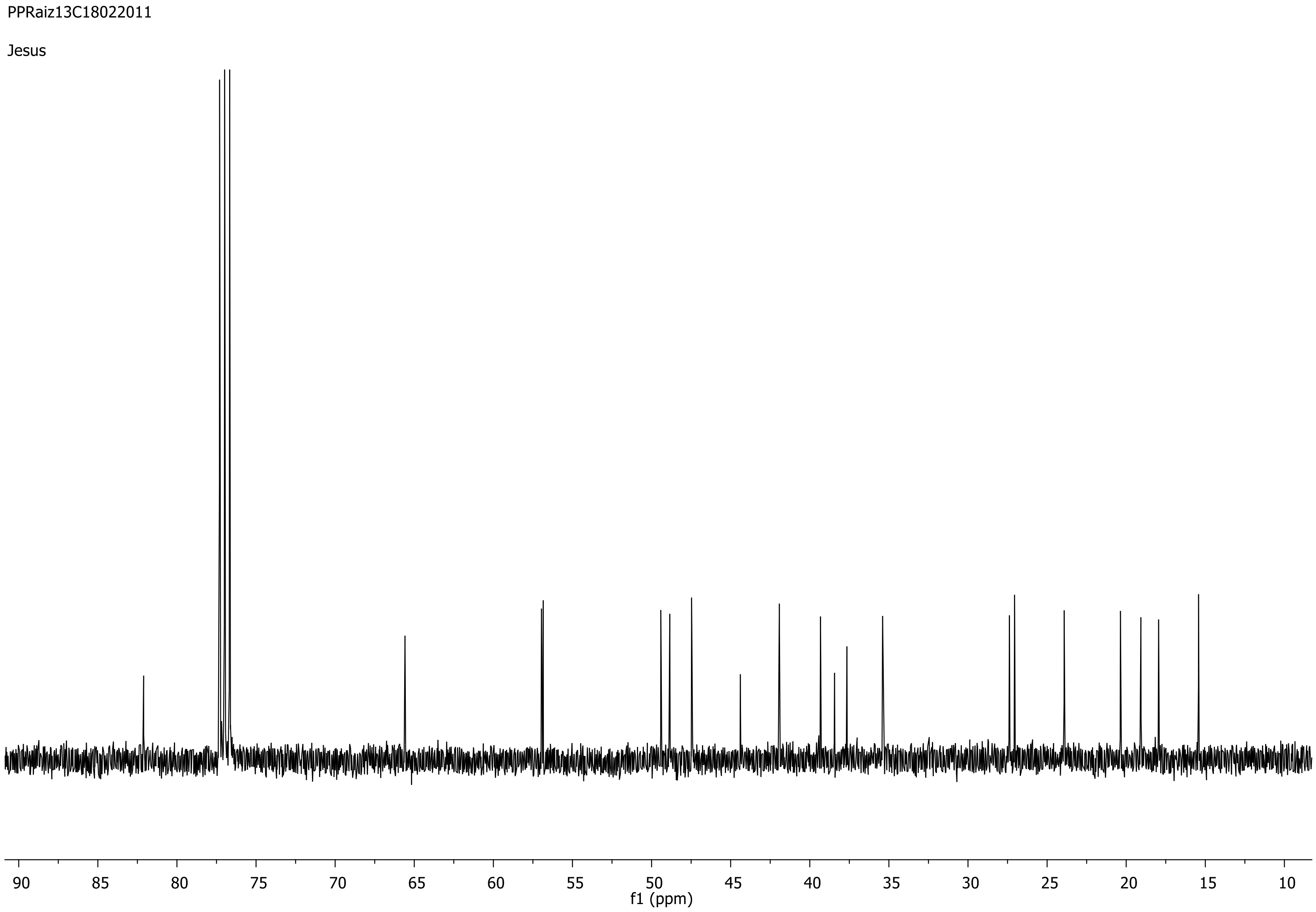

Figure S2: ^{13}C-NMR spectrum of phyllocladan-16α,19-diol (**1**)

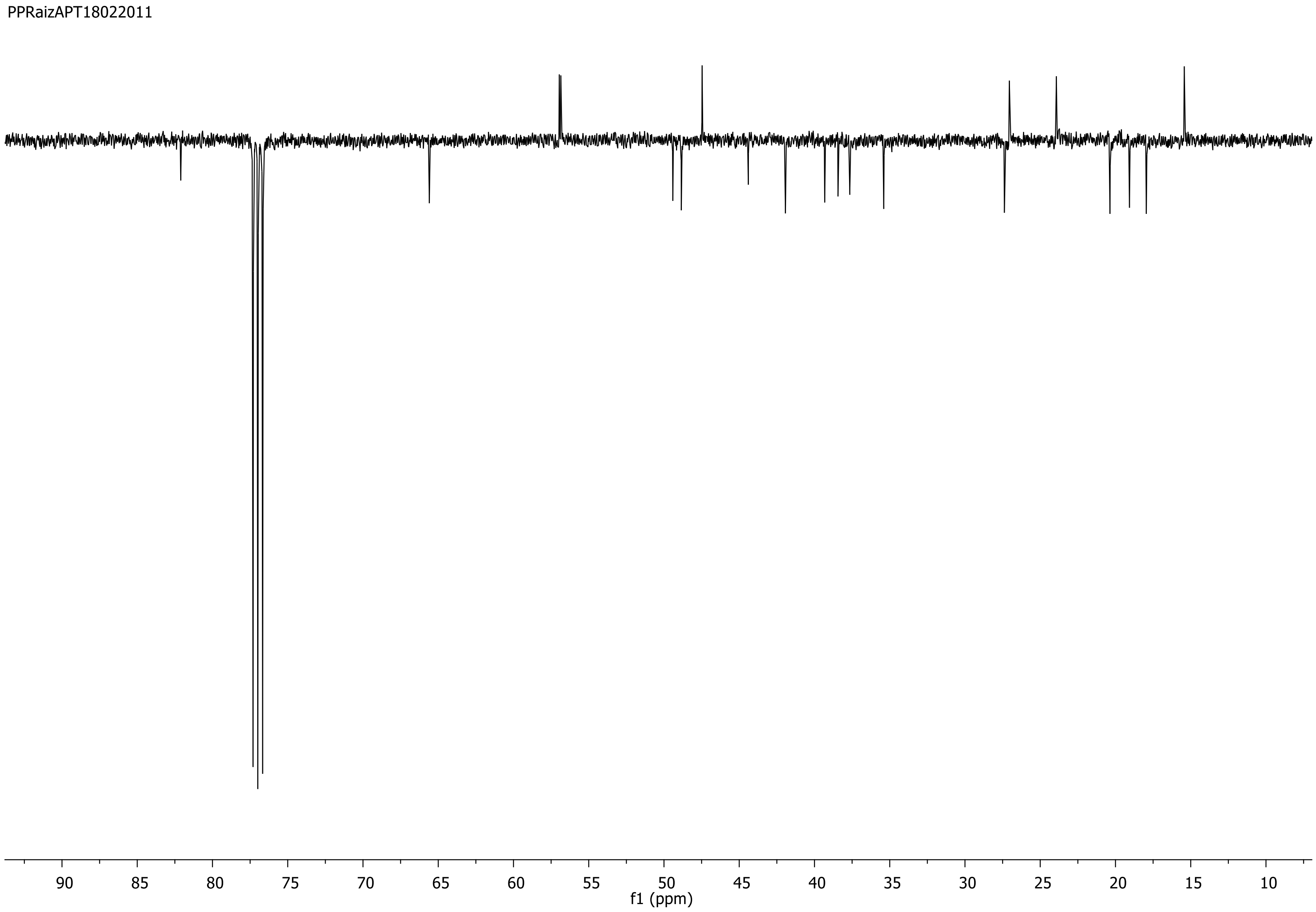

Figure S3: APT experiment spectrum of phyllocladan-16α,19-diol (**1**)

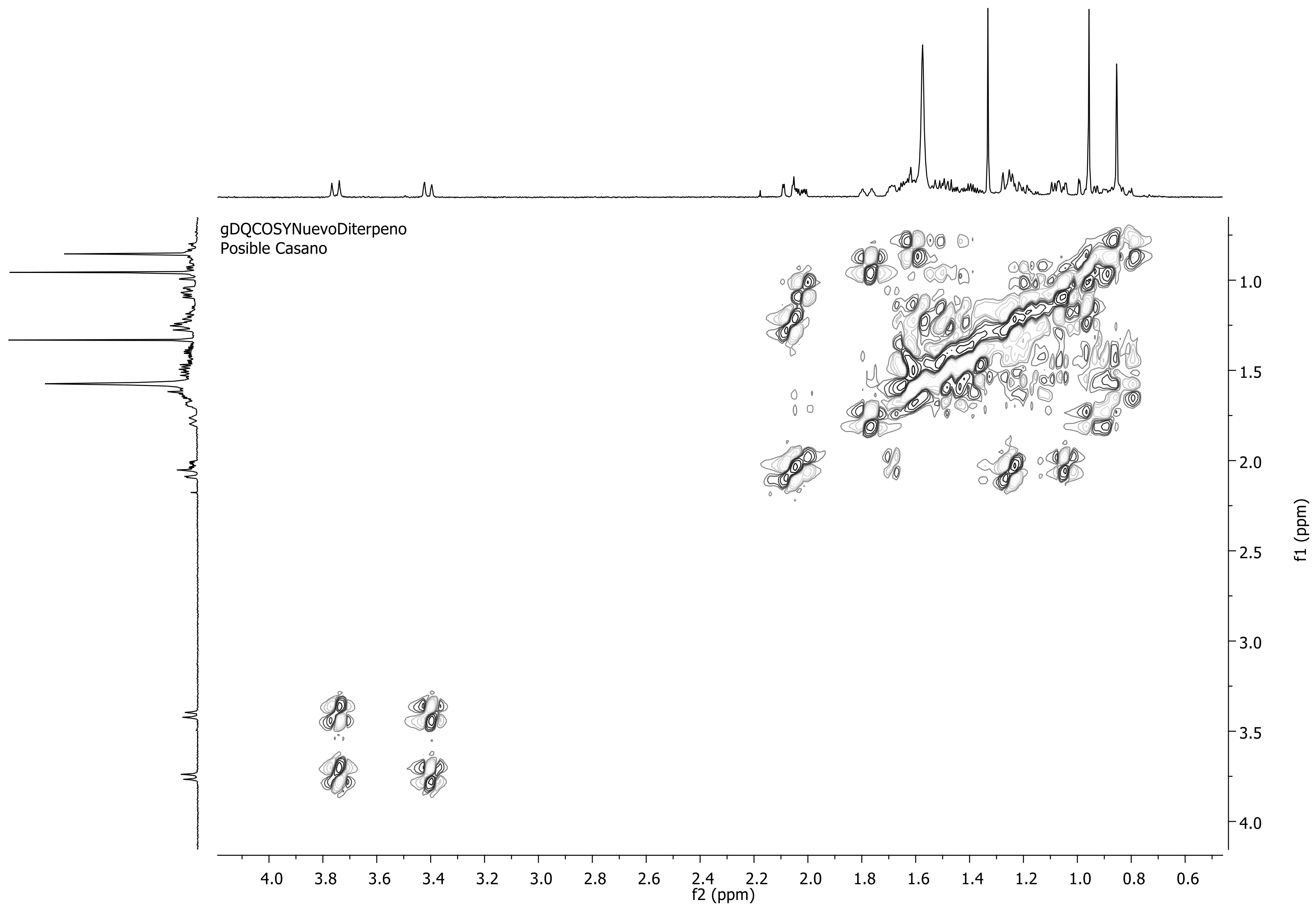

Figure S4: ^{1}H-^{1}H COSY spectrum of phyllocladan-16α,19-diol (**1**)

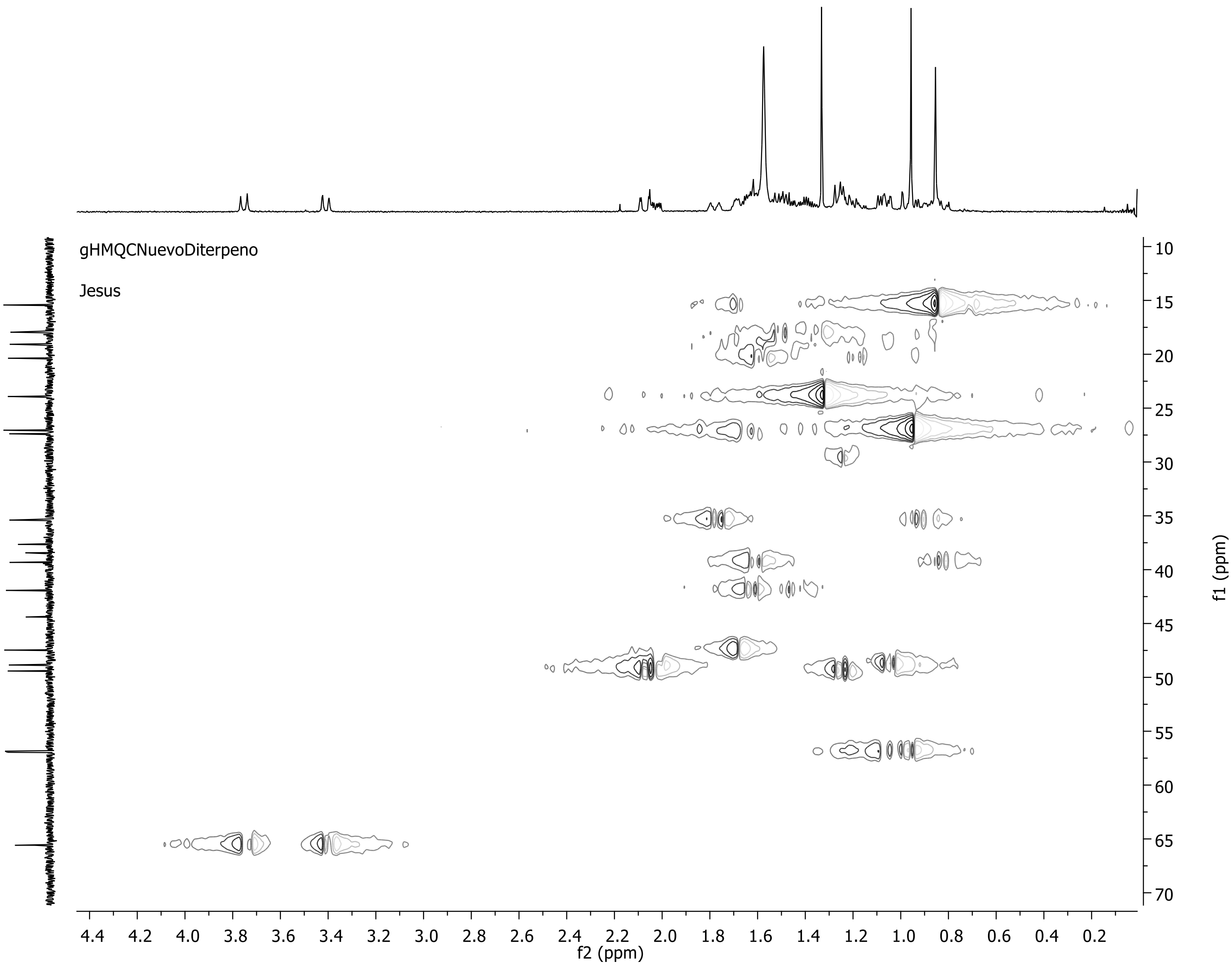

Figure S5: HSQC spectrum of phyllocladan-16α,19-diol (**1**)

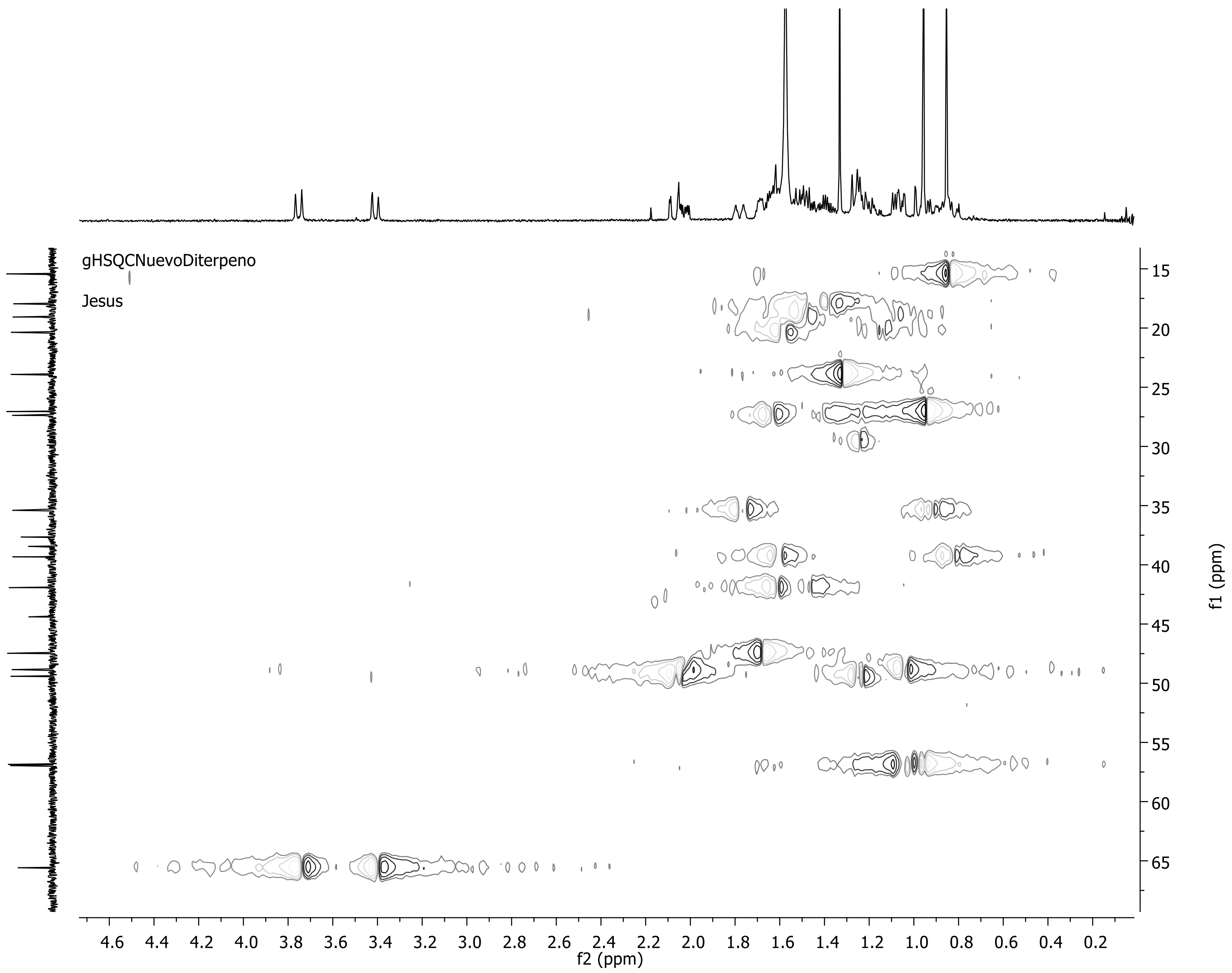

Figure S6: HSQC spectrum of phyllocladan-16α,19-diol (**1**)

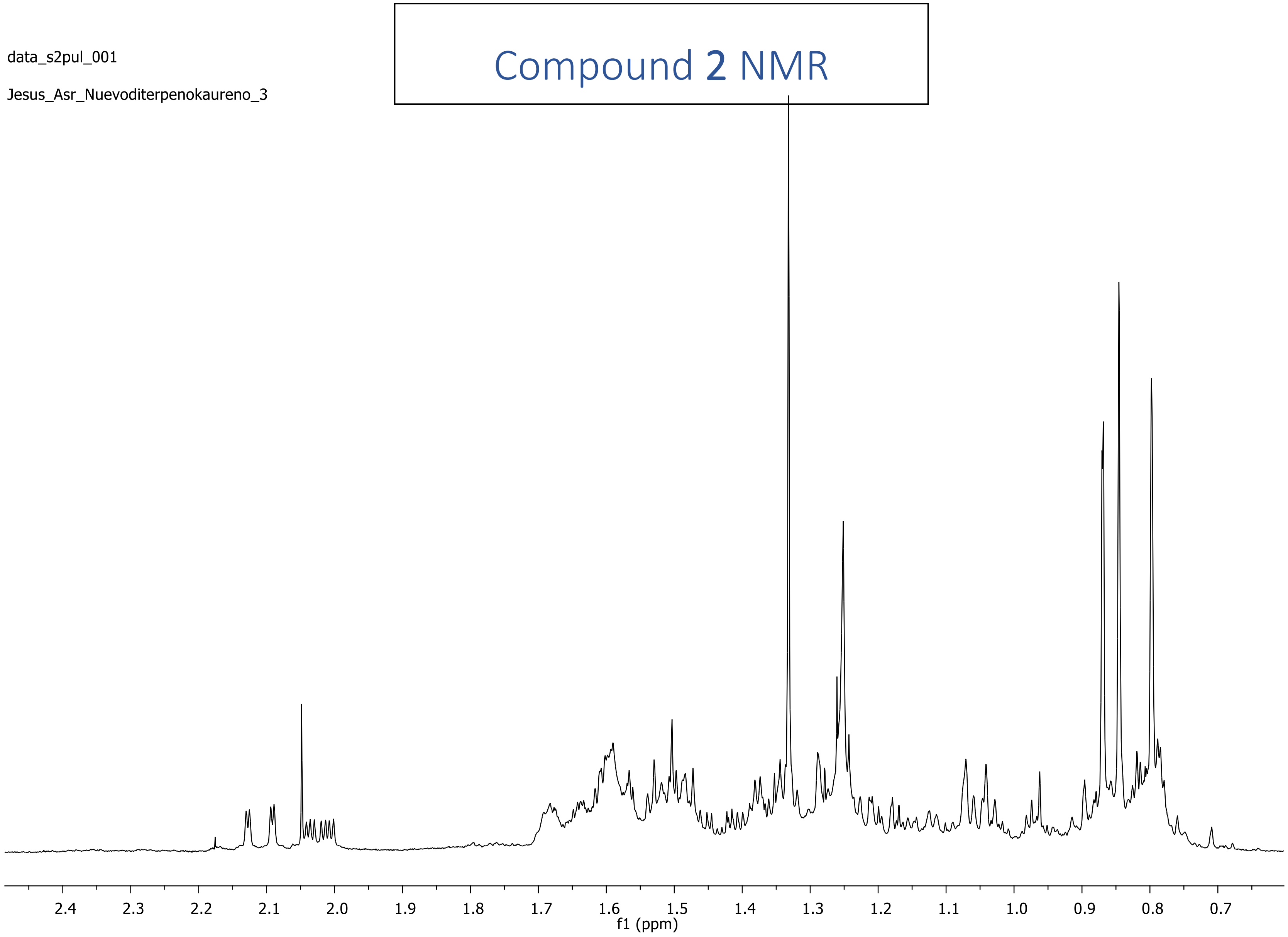

Figure S7: ^{1}H-NMR spectrum of phyllocladan-16α-ol (**2**)

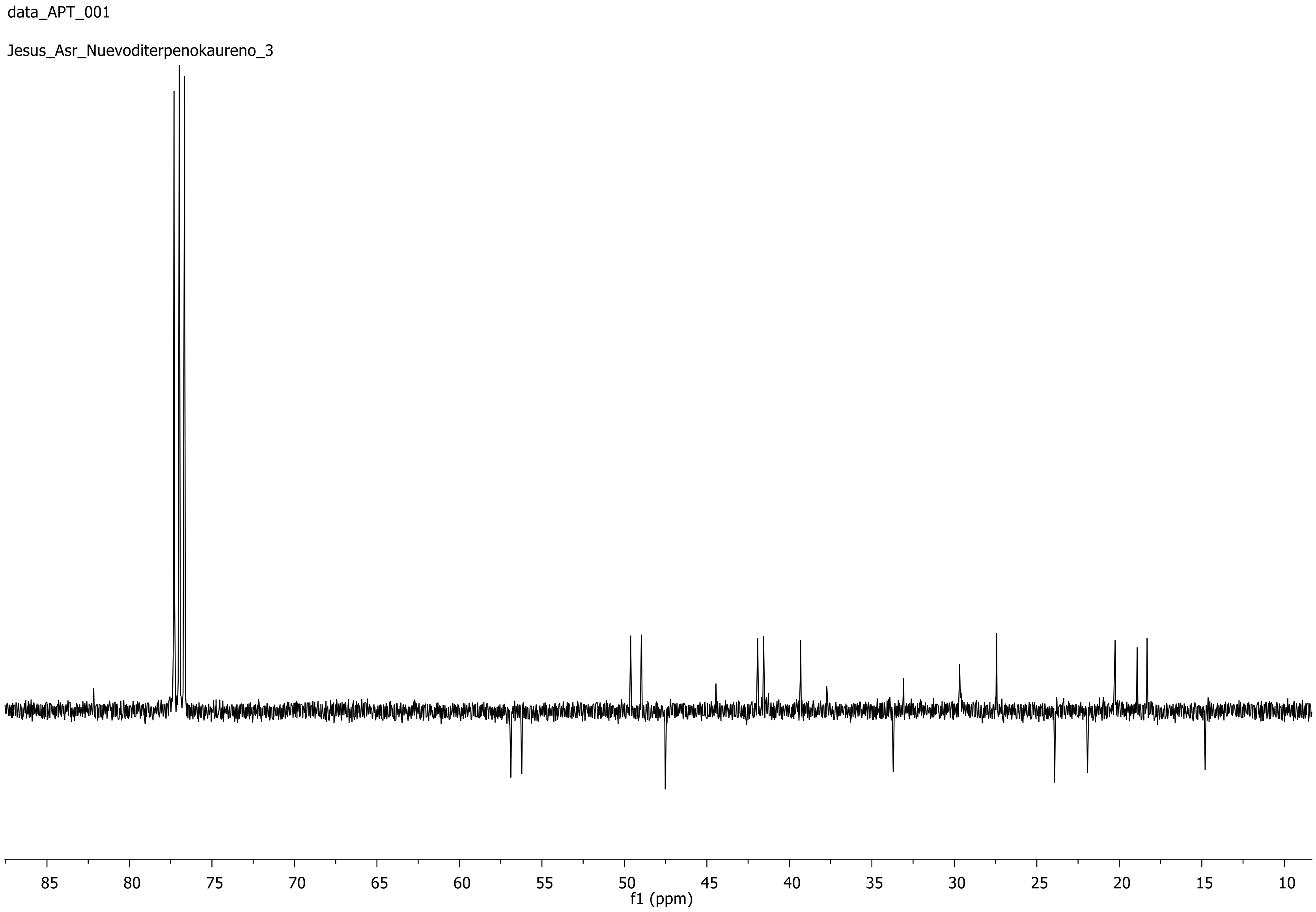

Figure S8: APT experiment spectrum of phyllocladan-16α-ol (**2**)

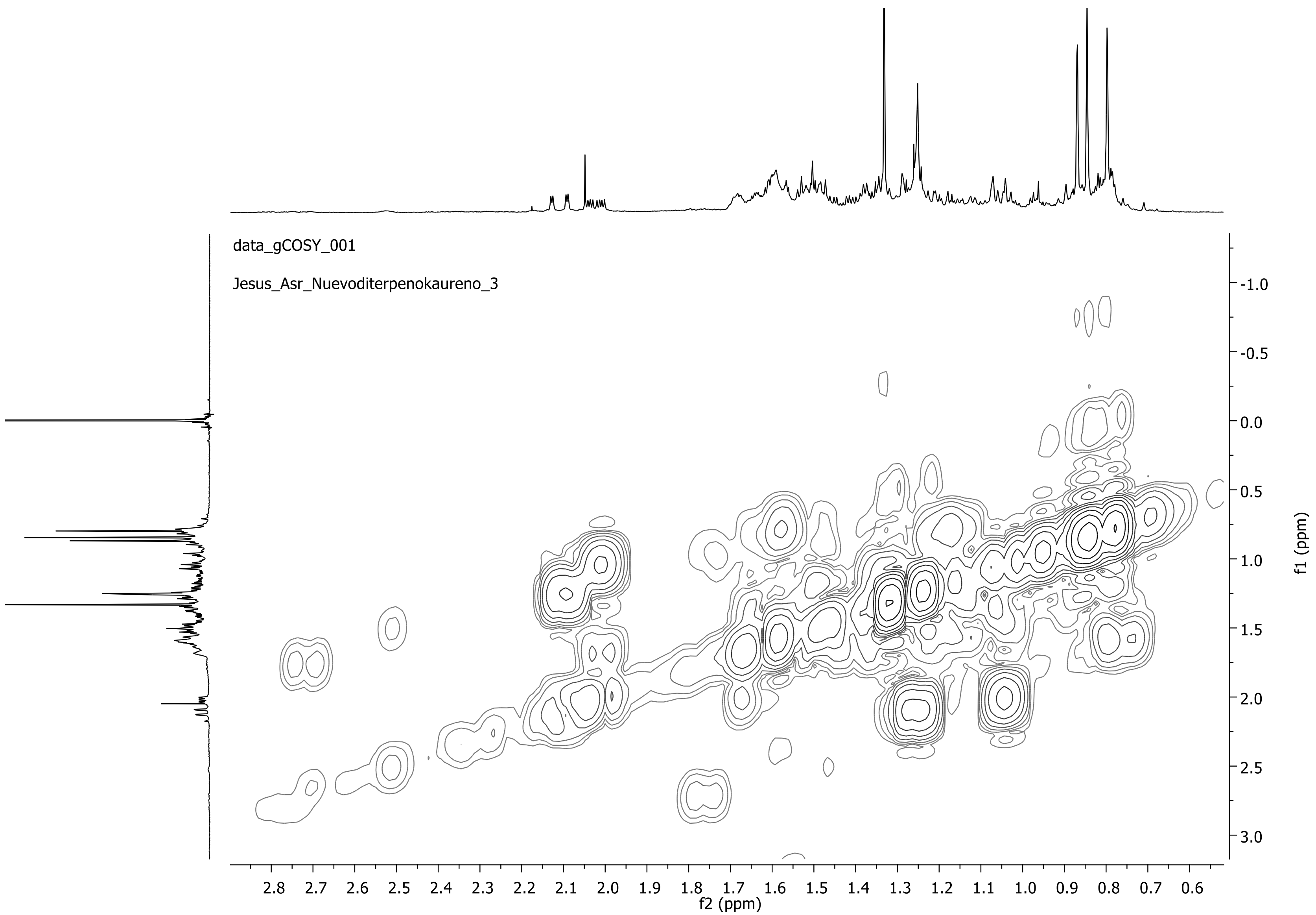

Figure S9: ^{1}H-^{1}H COSY spectrum of phyllocladan-16α-ol (**2**)

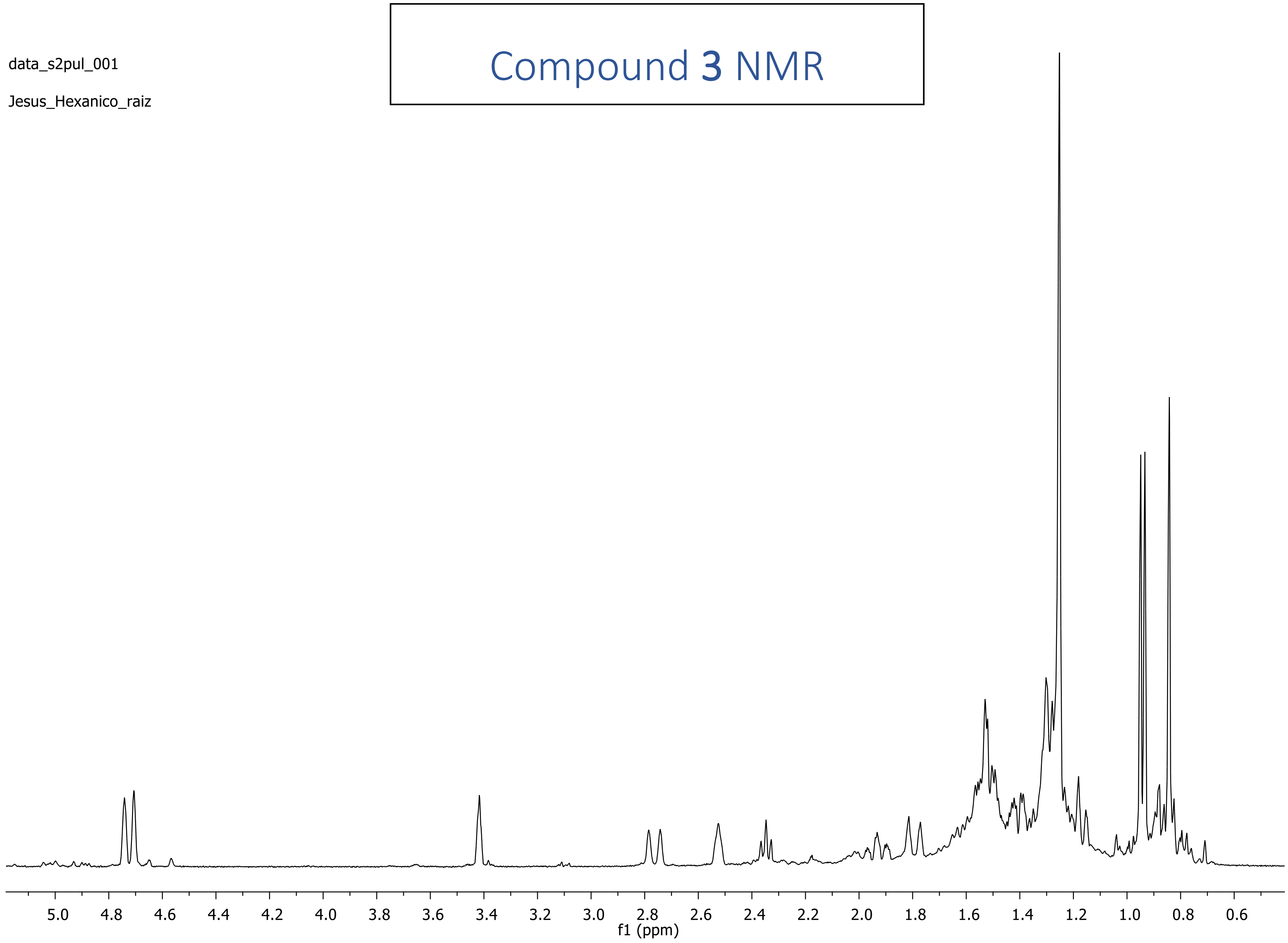

Figure S10: ^{1}H-NMR spectrum of phyllolad-16-en-3-ol (**3**)

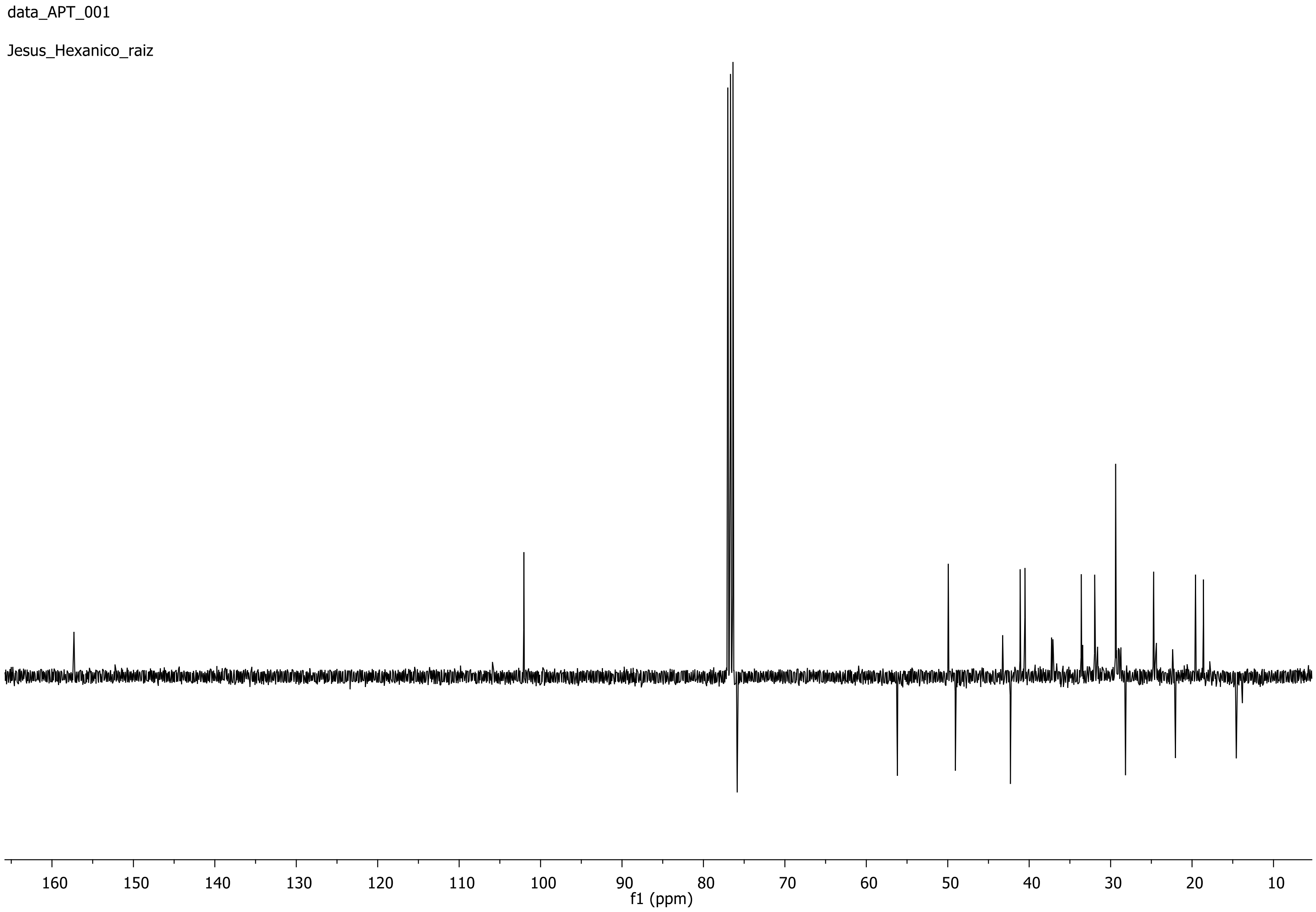

Figure S11: APT experiment spectrum of phylloclad-16-en-3-ol (**3**)

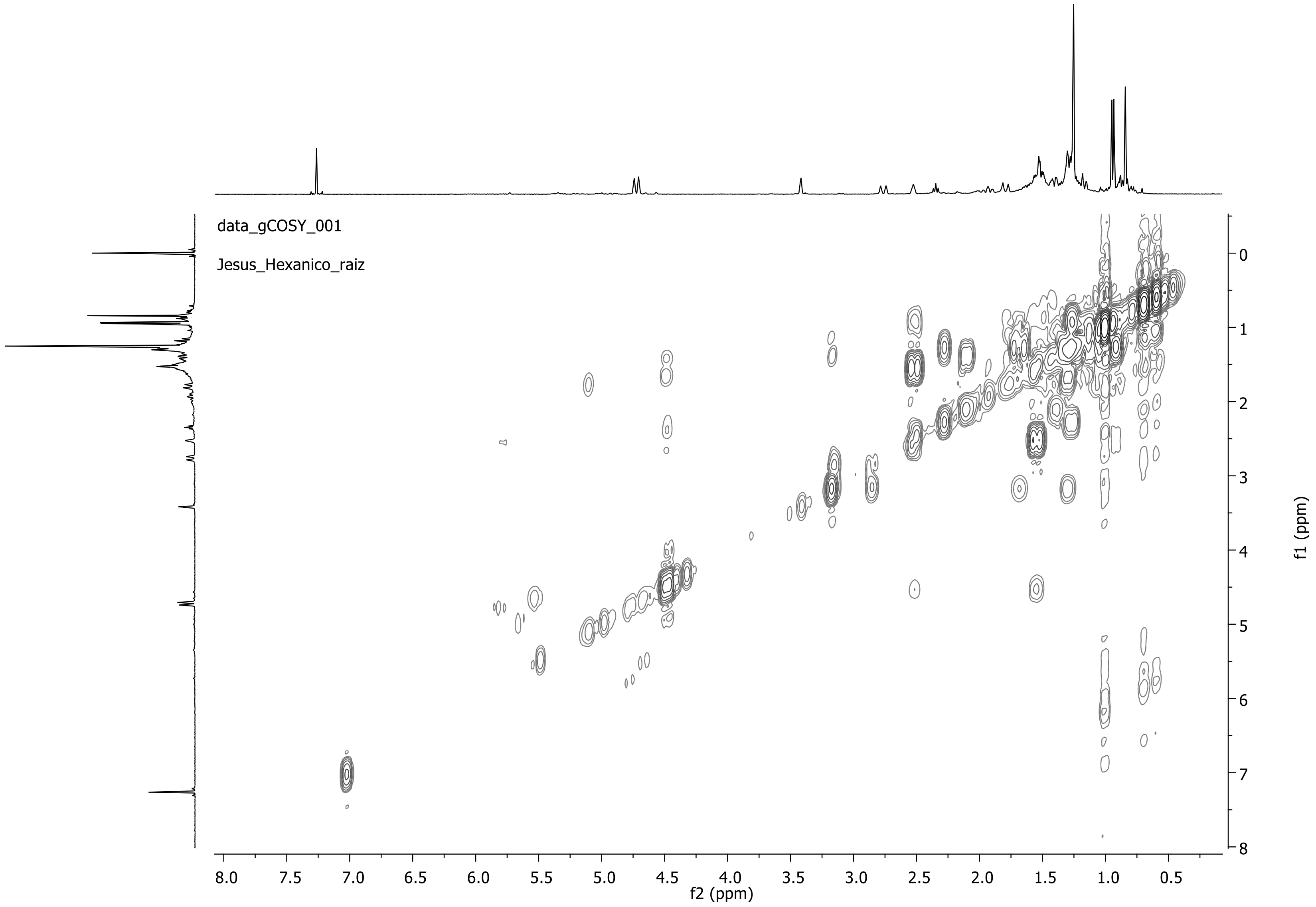

Figure S12: ^{1}H-^{1}H COSY spectrum of phyllocladd-16-en-3-ol (**3**)

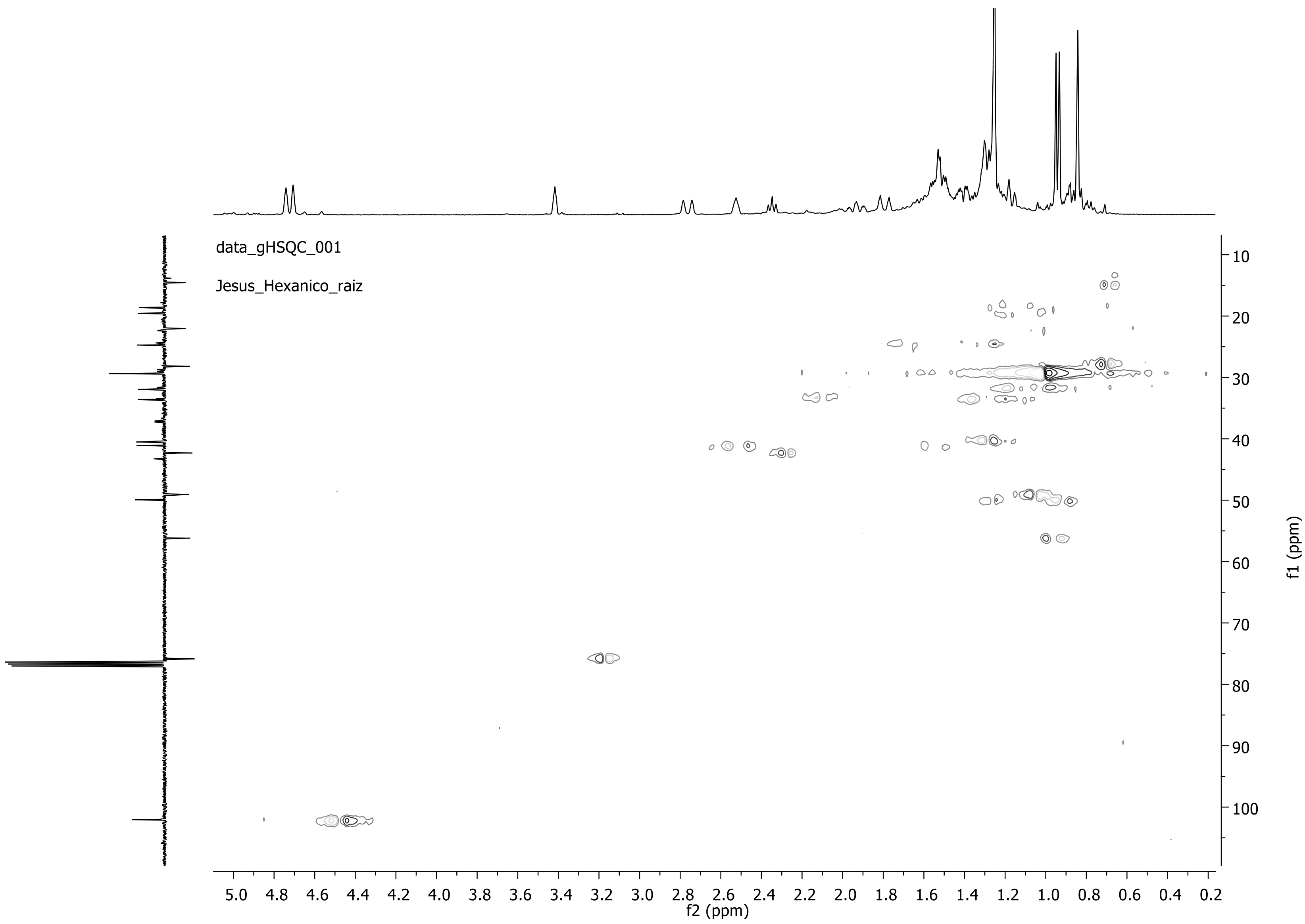

Figure S14: HSQC spectrum of phyllocladan-16-en-3-ol (**3**)

Compound **1** X-ray data diffraction

Table 1. Crystal data and structure refinement for C20H34O2.

Identification code	mtv03	
Empirical formula	C20 H34 O2	
Formula weight	306.47	
Temperature	293(2) K	
Wavelength	1.54184 A	
Crystal system, space group	Orthorhombic, P 21 21 21	
Unit cell dimensions	a = 7.0248(5) A	alpha = 90 deg.
	b = 14.6654(17) A	beta = 90 deg.
	c = 17.5721(16) A	gamma = 90 deg.
Volume	1810.3(3) A^3	
Z, Calculated density	4, 1.124 Mg/m^3	
Absorption coefficient	0.537 mm^-1	

F(000)	680
Crystal size	0.29 x 0.09 x 0.08 mm
Theta range for data collection	3.93 to 66.09 deg.
Limiting indices	-6<=h<=8, -17<=k<=15, -20<=l<=19
Reflections collected / unique	7379 / 3126 [R(int) = 0.0443]
Completeness to theta = 66.09	99.9 %
Absorption correction	Semi-empirical from equivalents
Max. and min. transmission	0.9583 and 0.8599
Refinement method	Full-matrix least-squares on F^2
Data / restraints / parameters	3126 / 0 / 210
Goodness-of-fit on F^2	1.036
Final R indices [I>2sigma(I)]	R1 = 0.0633, wR2 = 0.1573
R indices (all data)	R1 = 0.0881, wR2 = 0.1791
Absolute structure parameter	-0.2(5)
Largest diff. peak and hole	0.124 and -0.172 e.A^-3

Table 2. Atomic coordinates (x 10^4) and equivalent isotropic displacement parameters (A^2 x 10^3) for C20H34O2.
U(eq) is defined as one third of the trace of the orthogonalized Uij tensor.

	x	y	z	U(eq)
O(1)	1421(4)	2412(2)	5938(2)	79(1)
O(2)	794(4)	-1610(2)	10079(2)	74(1)
C(1)	409(7)	2833(2)	8709(2)	81(1)
C(2)	262(7)	3416(3)	7999(2)	89(1)
C(3)	2000(6)	3323(3)	7502(2)	81(1)
C(4)	2501(5)	2346(3)	7274(2)	70(1)
C(5)	2487(5)	1733(2)	8002(2)	64(1)
C(6)	2977(6)	733(3)	7861(2)	74(1)
C(7)	3532(5)	272(3)	8605(2)	70(1)
C(8)	2053(4)	341(2)	9233(2)	62(1)
C(9)	1327(5)	1331(2)	9308(2)	64(1)
C(10)	751(5)	1816(2)	8545(2)	64(1)
C(11)	-195(7)	1395(3)	9931(2)	82(1)
C(12)	290(6)	853(3)	10648(2)	82(1)
C(13)	1179(5)	-79(2)	10493(2)	69(1)
C(14)	2937(5)	58(3)	10000(2)	68(1)
C(15)	393(5)	-365(2)	9179(2)	68(1)
C(16)	-3(5)	-708(3)	9990(2)	67(1)

C(17)	-2115(6)	-760(3)	10189(3)	96(1)
C(18)	4531(5)	2366(3)	6940(2)	84(1)
C(19)	1114(5)	1997(3)	6658(2)	74(1)
C(20)	-1088(5)	1416(3)	8188(2)	81(1)

Table 3. Bond lengths [A] and angles [deg] for C20H34O2.

O(1)-C(19)	1.421(5)
O(1)-H(1O)	0.77(4)
O(2)-C(16)	1.444(4)
O(2)-H(2O)	0.86(4)
C(1)-C(2)	1.515(5)
C(1)-C(10)	1.539(5)
C(1)-H(1A)	0.9700
C(1)-H(1B)	0.9700
C(2)-C(3)	1.508(6)
C(2)-H(2A)	0.9700
C(2)-H(2B)	0.9700
C(3)-C(4)	1.529(6)
C(3)-H(3A)	0.9700
C(3)-H(3B)	0.9700
C(4)-C(18)	1.543(5)
C(4)-C(19)	1.544(5)
C(4)-C(5)	1.562(5)
C(5)-C(6)	1.527(5)
C(5)-C(10)	1.554(5)
C(5)-H(5)	0.9800
C(6)-C(7)	1.522(5)
C(6)-H(6A)	0.9700
C(6)-H(6B)	0.9700
C(7)-C(8)	1.519(4)
C(7)-H(7A)	0.9700

C(7)-H(7B)	0.9700
C(8)-C(14)	1.541(4)
C(8)-C(9)	1.545(5)
C(8)-C(15)	1.562(5)
C(9)-C(11)	1.534(5)
C(9)-C(10)	1.569(5)
C(9)-H(9)	0.9800
C(10)-C(20)	1.551(5)
C(11)-C(12)	1.528(5)
C(11)-H(11A)	0.9700
C(11)-H(11B)	0.9700
C(12)-C(13)	1.528(5)
C(12)-H(12A)	0.9700
C(12)-H(12B)	0.9700
C(13)-C(14)	1.522(5)
C(13)-C(16)	1.524(5)
C(13)-H(13)	0.9800
C(14)-H(14A)	0.9700
C(14)-H(14B)	0.9700
C(15)-C(16)	1.537(5)
C(15)-H(15A)	0.9700
C(15)-H(15B)	0.9700
C(16)-C(17)	1.526(5)
C(17)-H(17A)	0.9600
C(17)-H(17B)	0.9600
C(17)-H(17C)	0.9600
C(18)-H(18A)	0.9600
C(18)-H(18B)	0.9600
C(18)-H(18C)	0.9600

C(19)-H(19A)	0.9700
C(19)-H(19B)	0.9700
C(20)-H(20A)	0.9600
C(20)-H(20B)	0.9600
C(20)-H(20C)	0.9600

C(19)-O(1)-H(1O)	110(3)
C(16)-O(2)-H(2O)	110(3)
C(2)-C(1)-C(10)	113.8(3)
C(2)-C(1)-H(1A)	108.8
C(10)-C(1)-H(1A)	108.8
C(2)-C(1)-H(1B)	108.8
C(10)-C(1)-H(1B)	108.8
H(1A)-C(1)-H(1B)	107.7
C(3)-C(2)-C(1)	111.8(3)
C(3)-C(2)-H(2A)	109.3
C(1)-C(2)-H(2A)	109.3
C(3)-C(2)-H(2B)	109.3
C(1)-C(2)-H(2B)	109.3
H(2A)-C(2)-H(2B)	107.9
C(2)-C(3)-C(4)	115.0(3)
C(2)-C(3)-H(3A)	108.5
C(4)-C(3)-H(3A)	108.5
C(2)-C(3)-H(3B)	108.5
C(4)-C(3)-H(3B)	108.5
H(3A)-C(3)-H(3B)	107.5
C(3)-C(4)-C(18)	107.1(3)
C(3)-C(4)-C(19)	110.4(3)
C(18)-C(4)-C(19)	108.8(3)

C(3)-C(4)-C(5)	108.9(3)
C(18)-C(4)-C(5)	109.2(3)
C(19)-C(4)-C(5)	112.3(3)
C(6)-C(5)-C(10)	110.5(3)
C(6)-C(5)-C(4)	114.8(3)
C(10)-C(5)-C(4)	117.6(3)
C(6)-C(5)-H(5)	104.0
C(10)-C(5)-H(5)	104.0
C(4)-C(5)-H(5)	104.0
C(7)-C(6)-C(5)	110.2(3)
C(7)-C(6)-H(6A)	109.6
C(5)-C(6)-H(6A)	109.6
C(7)-C(6)-H(6B)	109.6
C(5)-C(6)-H(6B)	109.6
H(6A)-C(6)-H(6B)	108.1
C(8)-C(7)-C(6)	114.8(3)
C(8)-C(7)-H(7A)	108.6
C(6)-C(7)-H(7A)	108.6
C(8)-C(7)-H(7B)	108.6
C(6)-C(7)-H(7B)	108.6
H(7A)-C(7)-H(7B)	107.6
C(7)-C(8)-C(14)	109.9(2)
C(7)-C(8)-C(9)	110.5(3)
C(14)-C(8)-C(9)	108.2(3)
C(7)-C(8)-C(15)	115.0(3)
C(14)-C(8)-C(15)	100.1(3)
C(9)-C(8)-C(15)	112.5(3)
C(11)-C(9)-C(8)	110.3(3)
C(11)-C(9)-C(10)	113.8(3)

C(8)-C(9)-C(10)	116.0(3)
C(11)-C(9)-H(9)	105.2
C(8)-C(9)-H(9)	105.2
C(10)-C(9)-H(9)	105.2
C(1)-C(10)-C(20)	108.2(3)
C(1)-C(10)-C(5)	108.2(3)
C(20)-C(10)-C(5)	112.1(3)
C(1)-C(10)-C(9)	108.7(3)
C(20)-C(10)-C(9)	112.9(3)
C(5)-C(10)-C(9)	106.7(3)
C(12)-C(11)-C(9)	113.7(3)
C(12)-C(11)-H(11A)	108.8
C(9)-C(11)-H(11A)	108.8
C(12)-C(11)-H(11B)	108.8
C(9)-C(11)-H(11B)	108.8
H(11A)-C(11)-H(11B)	107.7
C(13)-C(12)-C(11)	114.1(3)
C(13)-C(12)-H(12A)	108.7
C(11)-C(12)-H(12A)	108.7
C(13)-C(12)-H(12B)	108.7
C(11)-C(12)-H(12B)	108.7
H(12A)-C(12)-H(12B)	107.6
C(14)-C(13)-C(16)	101.1(3)
C(14)-C(13)-C(12)	108.4(3)
C(16)-C(13)-C(12)	115.0(3)
C(14)-C(13)-H(13)	110.7
C(16)-C(13)-H(13)	110.7
C(12)-C(13)-H(13)	110.7
C(13)-C(14)-C(8)	101.9(3)

C(13)-C(14)-H(14A)	111.4
C(8)-C(14)-H(14A)	111.4
C(13)-C(14)-H(14B)	111.4
C(8)-C(14)-H(14B)	111.4
H(14A)-C(14)-H(14B)	109.3
C(16)-C(15)-C(8)	107.2(3)
C(16)-C(15)-H(15A)	110.3
C(8)-C(15)-H(15A)	110.3
C(16)-C(15)-H(15B)	110.3
C(8)-C(15)-H(15B)	110.3
H(15A)-C(15)-H(15B)	108.5
O(2)-C(16)-C(13)	106.2(3)
O(2)-C(16)-C(17)	107.8(3)
C(13)-C(16)-C(17)	115.3(3)
O(2)-C(16)-C(15)	109.3(3)
C(13)-C(16)-C(15)	103.9(3)
C(17)-C(16)-C(15)	113.9(3)
C(16)-C(17)-H(17A)	109.5
C(16)-C(17)-H(17B)	109.5
H(17A)-C(17)-H(17B)	109.5
C(16)-C(17)-H(17C)	109.5
H(17A)-C(17)-H(17C)	109.5
H(17B)-C(17)-H(17C)	109.5
C(4)-C(18)-H(18A)	109.5
C(4)-C(18)-H(18B)	109.5
H(18A)-C(18)-H(18B)	109.5
C(4)-C(18)-H(18C)	109.5
H(18A)-C(18)-H(18C)	109.5
H(18B)-C(18)-H(18C)	109.5

O(1)-C(19)-C(4)	112.8(3)
O(1)-C(19)-H(19A)	109.0
C(4)-C(19)-H(19A)	109.0
O(1)-C(19)-H(19B)	109.0
C(4)-C(19)-H(19B)	109.0
H(19A)-C(19)-H(19B)	107.8
C(10)-C(20)-H(20A)	109.5
C(10)-C(20)-H(20B)	109.5
H(20A)-C(20)-H(20B)	109.5
C(10)-C(20)-H(20C)	109.5
H(20A)-C(20)-H(20C)	109.5
H(20B)-C(20)-H(20C)	109.5

Symmetry transformations used to generate equivalent atoms:

Table 4. Anisotropic displacement parameters (A^2 x 10^3) for C20H34O2.

The anisotropic displacement factor exponent takes the form:

-2 pi^2 [h^2 a*^2 U11 + ... + 2 h k a* b* U12]

	U11	U22	U33	U23	U13	U12
O(1)	72(2)	96(2)	68(2)	2(2)	-7(1)	20(2)
O(2)	71(2)	70(2)	82(2)	2(1)	-15(1)	-3(1)
C(1)	90(3)	69(2)	84(2)	-4(2)	3(2)	15(2)
C(2)	100(3)	74(2)	91(3)	-3(2)	-7(2)	22(2)
C(3)	93(3)	73(2)	76(2)	6(2)	-15(2)	-1(2)
C(4)	57(2)	84(3)	70(2)	-2(2)	-11(2)	-1(2)
C(5)	53(2)	70(2)	69(2)	-4(2)	-8(2)	1(2)
C(6)	66(2)	84(2)	71(2)	-5(2)	1(2)	11(2)
C(7)	56(2)	70(2)	82(2)	3(2)	5(2)	9(2)
C(8)	49(2)	72(2)	65(2)	-8(2)	-8(2)	7(2)
C(9)	60(2)	64(2)	67(2)	-7(2)	-7(2)	3(2)
C(10)	55(2)	69(2)	67(2)	-5(2)	-2(2)	8(2)
C(11)	91(3)	75(2)	79(2)	-8(2)	11(2)	22(2)
C(12)	92(3)	83(3)	69(2)	-8(2)	2(2)	7(2)
C(13)	66(2)	78(2)	62(2)	-1(2)	-11(2)	0(2)
C(14)	55(2)	74(2)	76(2)	-5(2)	-15(2)	0(2)
C(15)	55(2)	74(2)	77(2)	-5(2)	-11(2)	1(2)
C(16)	51(2)	75(2)	74(2)	-5(2)	-7(2)	-2(2)
C(17)	55(2)	113(3)	120(4)	2(3)	4(2)	-1(2)

C(18)	60(2)	105(3)	88(2)	8(2)	-9(2)	-5(2)
C(19)	60(2)	92(3)	70(2)	-3(2)	-10(2)	-1(2)
C(20)	53(2)	107(3)	82(2)	8(2)	-7(2)	8(2)

Table 5. Hydrogen coordinates (x 10^4) and isotropic displacement parameters (A^2 x 10^3) for C20H34O2.

	x	y	z	U(eq)
H(1A)	-758	2896	8999	97
H(1B)	1444	3061	9021	97
H(2A)	-856	3236	7712	106
H(2B)	109	4049	8145	106
H(3A)	3079	3585	7768	97
H(3B)	1801	3677	7042	97
H(5)	3558	1962	8304	76
H(6A)	1887	423	7642	88
H(6B)	4024	692	7503	88
H(7A)	3778	-367	8505	84
H(7B)	4709	542	8786	84
H(9)	2414	1680	9502	76
H(11A)	-367	2030	10068	98
H(11B)	-1393	1174	9729	98
H(12A)	1166	1209	10955	98
H(12B)	-864	768	10943	98
H(13)	1515	-384	10971	82
H(14A)	3664	-502	9952	82
H(14B)	3751	534	10203	82
H(15A)	-735	-79	8969	82

H(15B)	754	-869	8852	82
H(17A)	-2744	-1167	9843	144
H(17B)	-2671	-164	10150	144
H(17C)	-2258	-983	10700	144
H(18A)	4852	1772	6748	126
H(18B)	5419	2536	7330	126
H(18C)	4585	2802	6533	126
H(19A)	1258	1342	6608	89
H(19B)	-182	2118	6820	89
H(20A)	-2055	1368	8572	121
H(20B)	-824	822	7984	121
H(20C)	-1522	1810	7788	121
H(2O)	350(70)	-1970(30)	9740(20)	97(15)
H(1O)	2200(60)	2150(30)	5720(30)	87(15)

Table 6. Torsion angles [deg] for C20H34O2.

C(10)-C(1)-C(2)-C(3)	-56.2(5)
C(1)-C(2)-C(3)-C(4)	54.7(5)
C(2)-C(3)-C(4)-C(18)	-166.8(3)
C(2)-C(3)-C(4)-C(19)	74.9(4)
C(2)-C(3)-C(4)-C(5)	-48.8(4)
C(3)-C(4)-C(5)-C(6)	-179.7(3)
C(18)-C(4)-C(5)-C(6)	-63.0(4)
C(19)-C(4)-C(5)-C(6)	57.8(4)
C(3)-C(4)-C(5)-C(10)	47.7(4)
C(18)-C(4)-C(5)-C(10)	164.4(3)
C(19)-C(4)-C(5)-C(10)	-74.8(4)
C(10)-C(5)-C(6)-C(7)	-61.9(4)
C(4)-C(5)-C(6)-C(7)	162.2(3)
C(5)-C(6)-C(7)-C(8)	55.7(4)
C(6)-C(7)-C(8)-C(14)	-166.8(3)
C(6)-C(7)-C(8)-C(9)	-47.4(4)
C(6)-C(7)-C(8)-C(15)	81.2(4)
C(7)-C(8)-C(9)-C(11)	178.9(3)
C(14)-C(8)-C(9)-C(11)	-60.7(4)
C(15)-C(8)-C(9)-C(11)	48.9(4)
C(7)-C(8)-C(9)-C(10)	47.7(4)
C(14)-C(8)-C(9)-C(10)	168.1(3)
C(15)-C(8)-C(9)-C(10)	-82.3(4)
C(2)-C(1)-C(10)-C(20)	-69.7(4)
C(2)-C(1)-C(10)-C(5)	52.0(4)

C(2)-C(1)-C(10)-C(9)	167.5(4)
C(6)-C(5)-C(10)-C(1)	176.2(3)
C(4)-C(5)-C(10)-C(1)	-49.3(4)
C(6)-C(5)-C(10)-C(20)	-64.6(4)
C(4)-C(5)-C(10)-C(20)	69.9(4)
C(6)-C(5)-C(10)-C(9)	59.4(3)
C(4)-C(5)-C(10)-C(9)	-166.1(3)
C(11)-C(9)-C(10)-C(1)	60.3(4)
C(8)-C(9)-C(10)-C(1)	-170.1(3)
C(11)-C(9)-C(10)-C(20)	-59.7(4)
C(8)-C(9)-C(10)-C(20)	69.9(4)
C(11)-C(9)-C(10)-C(5)	176.8(3)
C(8)-C(9)-C(10)-C(5)	-53.7(4)
C(8)-C(9)-C(11)-C(12)	42.6(4)
C(10)-C(9)-C(11)-C(12)	174.9(3)
C(9)-C(11)-C(12)-C(13)	-40.9(5)
C(11)-C(12)-C(13)-C(14)	55.9(4)
C(11)-C(12)-C(13)-C(16)	-56.4(5)
C(16)-C(13)-C(14)-C(8)	51.1(3)
C(12)-C(13)-C(14)-C(8)	-70.2(3)
C(7)-C(8)-C(14)-C(13)	-165.2(3)
C(9)-C(8)-C(14)-C(13)	74.0(3)
C(15)-C(8)-C(14)-C(13)	-43.8(3)
C(7)-C(8)-C(15)-C(16)	138.7(3)
C(14)-C(8)-C(15)-C(16)	21.0(3)
C(9)-C(8)-C(15)-C(16)	-93.7(3)
C(14)-C(13)-C(16)-O(2)	78.6(3)
C(12)-C(13)-C(16)-O(2)	-164.9(3)
C(14)-C(13)-C(16)-C(17)	-162.0(4)

C(12)-C(13)-C(16)-C(17)	-45.5(5)
C(14)-C(13)-C(16)-C(15)	-36.6(3)
C(12)-C(13)-C(16)-C(15)	79.8(4)
C(8)-C(15)-C(16)-O(2)	-103.7(3)
C(8)-C(15)-C(16)-C(13)	9.4(4)
C(8)-C(15)-C(16)-C(17)	135.7(3)
C(3)-C(4)-C(19)-O(1)	71.9(4)
C(18)-C(4)-C(19)-O(1)	-45.3(5)
C(5)-C(4)-C(19)-O(1)	-166.4(3)

Symmetry transformations used to generate equivalent atoms:

Table 7. Hydrogen bonds for C20H34O2 [A and deg.].

D-H...A	d(D-H)	d(H...A)	d(D...A)	<(DHA)
O(1)-H(1O)...O(2)#1	0.77(4)	1.97(4)	2.736(4)	173(4)
O(2)-H(2O)...O(1)#2	0.86(4)	1.94(5)	2.770(4)	161(4)

Symmetry transformations used to generate equivalent atoms:

#1 -x+1/2,-y,z-1/2 #2 -x,y-1/2,-z+3/2

Table 1. Crystal data and structure refinement for C20H34O2.

Identification code	mtv03
Empirical formula	C20 H34 O2
Formula weight	306.47
Temperature	293(2) K
Wavelength	1.54184 A
Crystal system, space group	Orthorhombic, P 21 21 21
Unit cell dimensions	a = 7.0248(5) A alpha = 90 deg.
	b = 14.6654(17) A beta = 90 deg.
	c = 17.5721(16) A gamma = 90 deg.
Volume	1810.3(3) A^3
Z, Calculated density	4, 1.124 Mg/m^3
Absorption coefficient	0.537 mm^-1
F(000)	680
Crystal size	0.29 x 0.09 x 0.08 mm

Theta range for data collection   3.93 to 66.09 deg.

Limiting indices   -6<=h<=8, -17<=k<=15, -20<=l<=19

Reflections collected / unique   7379 / 3126 [R(int) = 0.0443]

Completeness to theta = 66.09   99.9 %

Absorption correction   Semi-empirical from equivalents

Max. and min. transmission   0.9583 and 0.8599

Refinement method   Full-matrix least-squares on F^2

Data / restraints / parameters   3126 / 0 / 210

Goodness-of-fit on F^2   1.036

Final R indices [I>2sigma(I)]   R1 = 0.0633, wR2 = 0.1573

R indices (all data)   R1 = 0.0881, wR2 = 0.1791

Absolute structure parameter   -0.2(5)

Largest diff. peak and hole   0.124 and -0.172 e.A^-3

Table 2. Atomic coordinates (x 10^4) and equivalent isotropic displacement parameters (A^2 x 10^3) for C20H34O2.

U(eq) is defined as one third of the trace of the orthogonalized Uij tensor.

	x	y	z	U(eq)
O(1)	1421(4)	2412(2)	5938(2)	79(1)
O(2)	794(4)	-1610(2)	10079(2)	74(1)
C(1)	409(7)	2833(2)	8709(2)	81(1)
C(2)	262(7)	3416(3)	7999(2)	89(1)
C(3)	2000(6)	3323(3)	7502(2)	81(1)
C(4)	2501(5)	2346(3)	7274(2)	70(1)
C(5)	2487(5)	1733(2)	8002(2)	64(1)
C(6)	2977(6)	733(3)	7861(2)	74(1)
C(7)	3532(5)	272(3)	8605(2)	70(1)
C(8)	2053(4)	341(2)	9233(2)	62(1)
C(9)	1327(5)	1331(2)	9308(2)	64(1)
C(10)	751(5)	1816(2)	8545(2)	64(1)
C(11)	-195(7)	1395(3)	9931(2)	82(1)
C(12)	290(6)	853(3)	10648(2)	82(1)
C(13)	1179(5)	-79(2)	10493(2)	69(1)
C(14)	2937(5)	58(3)	10000(2)	68(1)
C(15)	393(5)	-365(2)	9179(2)	68(1)
C(16)	-3(5)	-708(3)	9990(2)	67(1)

C(17)	-2115(6)	-760(3)	10189(3)	96(1)
C(18)	4531(5)	2366(3)	6940(2)	84(1)
C(19)	1114(5)	1997(3)	6658(2)	74(1)
C(20)	-1088(5)	1416(3)	8188(2)	81(1)

Table 3. Bond lengths [A] and angles [deg] for C20H34O2.

O(1)-C(19)	1.421(5)
O(1)-H(1O)	0.77(4)
O(2)-C(16)	1.444(4)
O(2)-H(2O)	0.86(4)
C(1)-C(2)	1.515(5)
C(1)-C(10)	1.539(5)
C(1)-H(1A)	0.9700
C(1)-H(1B)	0.9700
C(2)-C(3)	1.508(6)
C(2)-H(2A)	0.9700
C(2)-H(2B)	0.9700
C(3)-C(4)	1.529(6)
C(3)-H(3A)	0.9700
C(3)-H(3B)	0.9700
C(4)-C(18)	1.543(5)
C(4)-C(19)	1.544(5)
C(4)-C(5)	1.562(5)
C(5)-C(6)	1.527(5)
C(5)-C(10)	1.554(5)
C(5)-H(5)	0.9800
C(6)-C(7)	1.522(5)
C(6)-H(6A)	0.9700
C(6)-H(6B)	0.9700
C(7)-C(8)	1.519(4)
C(7)-H(7A)	0.9700

C(7)-H(7B)	0.9700
C(8)-C(14)	1.541(4)
C(8)-C(9)	1.545(5)
C(8)-C(15)	1.562(5)
C(9)-C(11)	1.534(5)
C(9)-C(10)	1.569(5)
C(9)-H(9)	0.9800
C(10)-C(20)	1.551(5)
C(11)-C(12)	1.528(5)
C(11)-H(11A)	0.9700
C(11)-H(11B)	0.9700
C(12)-C(13)	1.528(5)
C(12)-H(12A)	0.9700
C(12)-H(12B)	0.9700
C(13)-C(14)	1.522(5)
C(13)-C(16)	1.524(5)
C(13)-H(13)	0.9800
C(14)-H(14A)	0.9700
C(14)-H(14B)	0.9700
C(15)-C(16)	1.537(5)
C(15)-H(15A)	0.9700
C(15)-H(15B)	0.9700
C(16)-C(17)	1.526(5)
C(17)-H(17A)	0.9600
C(17)-H(17B)	0.9600
C(17)-H(17C)	0.9600
C(18)-H(18A)	0.9600
C(18)-H(18B)	0.9600
C(18)-H(18C)	0.9600

C(19)-H(19A)	0.9700
C(19)-H(19B)	0.9700
C(20)-H(20A)	0.9600
C(20)-H(20B)	0.9600
C(20)-H(20C)	0.9600

C(19)-O(1)-H(1O)	110(3)
C(16)-O(2)-H(2O)	110(3)
C(2)-C(1)-C(10)	113.8(3)
C(2)-C(1)-H(1A)	108.8
C(10)-C(1)-H(1A)	108.8
C(2)-C(1)-H(1B)	108.8
C(10)-C(1)-H(1B)	108.8
H(1A)-C(1)-H(1B)	107.7
C(3)-C(2)-C(1)	111.8(3)
C(3)-C(2)-H(2A)	109.3
C(1)-C(2)-H(2A)	109.3
C(3)-C(2)-H(2B)	109.3
C(1)-C(2)-H(2B)	109.3
H(2A)-C(2)-H(2B)	107.9
C(2)-C(3)-C(4)	115.0(3)
C(2)-C(3)-H(3A)	108.5
C(4)-C(3)-H(3A)	108.5
C(2)-C(3)-H(3B)	108.5
C(4)-C(3)-H(3B)	108.5
H(3A)-C(3)-H(3B)	107.5
C(3)-C(4)-C(18)	107.1(3)
C(3)-C(4)-C(19)	110.4(3)
C(18)-C(4)-C(19)	108.8(3)

C(3)-C(4)-C(5)	108.9(3)
C(18)-C(4)-C(5)	109.2(3)
C(19)-C(4)-C(5)	112.3(3)
C(6)-C(5)-C(10)	110.5(3)
C(6)-C(5)-C(4)	114.8(3)
C(10)-C(5)-C(4)	117.6(3)
C(6)-C(5)-H(5)	104.0
C(10)-C(5)-H(5)	104.0
C(4)-C(5)-H(5)	104.0
C(7)-C(6)-C(5)	110.2(3)
C(7)-C(6)-H(6A)	109.6
C(5)-C(6)-H(6A)	109.6
C(7)-C(6)-H(6B)	109.6
C(5)-C(6)-H(6B)	109.6
H(6A)-C(6)-H(6B)	108.1
C(8)-C(7)-C(6)	114.8(3)
C(8)-C(7)-H(7A)	108.6
C(6)-C(7)-H(7A)	108.6
C(8)-C(7)-H(7B)	108.6
C(6)-C(7)-H(7B)	108.6
H(7A)-C(7)-H(7B)	107.6
C(7)-C(8)-C(14)	109.9(2)
C(7)-C(8)-C(9)	110.5(3)
C(14)-C(8)-C(9)	108.2(3)
C(7)-C(8)-C(15)	115.0(3)
C(14)-C(8)-C(15)	100.1(3)
C(9)-C(8)-C(15)	112.5(3)
C(11)-C(9)-C(8)	110.3(3)
C(11)-C(9)-C(10)	113.8(3)

C(8)-C(9)-C(10)	116.0(3)
C(11)-C(9)-H(9)	105.2
C(8)-C(9)-H(9)	105.2
C(10)-C(9)-H(9)	105.2
C(1)-C(10)-C(20)	108.2(3)
C(1)-C(10)-C(5)	108.2(3)
C(20)-C(10)-C(5)	112.1(3)
C(1)-C(10)-C(9)	108.7(3)
C(20)-C(10)-C(9)	112.9(3)
C(5)-C(10)-C(9)	106.7(3)
C(12)-C(11)-C(9)	113.7(3)
C(12)-C(11)-H(11A)	108.8
C(9)-C(11)-H(11A)	108.8
C(12)-C(11)-H(11B)	108.8
C(9)-C(11)-H(11B)	108.8
H(11A)-C(11)-H(11B)	107.7
C(13)-C(12)-C(11)	114.1(3)
C(13)-C(12)-H(12A)	108.7
C(11)-C(12)-H(12A)	108.7
C(13)-C(12)-H(12B)	108.7
C(11)-C(12)-H(12B)	108.7
H(12A)-C(12)-H(12B)	107.6
C(14)-C(13)-C(16)	101.1(3)
C(14)-C(13)-C(12)	108.4(3)
C(16)-C(13)-C(12)	115.0(3)
C(14)-C(13)-H(13)	110.7
C(16)-C(13)-H(13)	110.7
C(12)-C(13)-H(13)	110.7
C(13)-C(14)-C(8)	101.9(3)

C(13)-C(14)-H(14A)	111.4
C(8)-C(14)-H(14A)	111.4
C(13)-C(14)-H(14B)	111.4
C(8)-C(14)-H(14B)	111.4
H(14A)-C(14)-H(14B)	109.3
C(16)-C(15)-C(8)	107.2(3)
C(16)-C(15)-H(15A)	110.3
C(8)-C(15)-H(15A)	110.3
C(16)-C(15)-H(15B)	110.3
C(8)-C(15)-H(15B)	110.3
H(15A)-C(15)-H(15B)	108.5
O(2)-C(16)-C(13)	106.2(3)
O(2)-C(16)-C(17)	107.8(3)
C(13)-C(16)-C(17)	115.3(3)
O(2)-C(16)-C(15)	109.3(3)
C(13)-C(16)-C(15)	103.9(3)
C(17)-C(16)-C(15)	113.9(3)
C(16)-C(17)-H(17A)	109.5
C(16)-C(17)-H(17B)	109.5
H(17A)-C(17)-H(17B)	109.5
C(16)-C(17)-H(17C)	109.5
H(17A)-C(17)-H(17C)	109.5
H(17B)-C(17)-H(17C)	109.5
C(4)-C(18)-H(18A)	109.5
C(4)-C(18)-H(18B)	109.5
H(18A)-C(18)-H(18B)	109.5
C(4)-C(18)-H(18C)	109.5
H(18A)-C(18)-H(18C)	109.5
H(18B)-C(18)-H(18C)	109.5

O(1)-C(19)-C(4)	112.8(3)
O(1)-C(19)-H(19A)	109.0
C(4)-C(19)-H(19A)	109.0
O(1)-C(19)-H(19B)	109.0
C(4)-C(19)-H(19B)	109.0
H(19A)-C(19)-H(19B)	107.8
C(10)-C(20)-H(20A)	109.5
C(10)-C(20)-H(20B)	109.5
H(20A)-C(20)-H(20B)	109.5
C(10)-C(20)-H(20C)	109.5
H(20A)-C(20)-H(20C)	109.5
H(20B)-C(20)-H(20C)	109.5

Symmetry transformations used to generate equivalent atoms:

Table 4. Anisotropic displacement parameters (A^2 x 10^3) for C20H34O2.

The anisotropic displacement factor exponent takes the form:

-2 pi^2 [h^2 a*^2 U11 + ... + 2 h k a* b* U12]

	U11	U22	U33	U23	U13	U12
O(1)	72(2)	96(2)	68(2)	2(2)	-7(1)	20(2)
O(2)	71(2)	70(2)	82(2)	2(1)	-15(1)	-3(1)
C(1)	90(3)	69(2)	84(2)	-4(2)	3(2)	15(2)
C(2)	100(3)	74(2)	91(3)	-3(2)	-7(2)	22(2)
C(3)	93(3)	73(2)	76(2)	6(2)	-15(2)	-1(2)
C(4)	57(2)	84(3)	70(2)	-2(2)	-11(2)	-1(2)
C(5)	53(2)	70(2)	69(2)	-4(2)	-8(2)	1(2)
C(6)	66(2)	84(2)	71(2)	-5(2)	1(2)	11(2)
C(7)	56(2)	70(2)	82(2)	3(2)	5(2)	9(2)
C(8)	49(2)	72(2)	65(2)	-8(2)	-8(2)	7(2)
C(9)	60(2)	64(2)	67(2)	-7(2)	-7(2)	3(2)
C(10)	55(2)	69(2)	67(2)	-5(2)	-2(2)	8(2)
C(11)	91(3)	75(2)	79(2)	-8(2)	11(2)	22(2)
C(12)	92(3)	83(3)	69(2)	-8(2)	2(2)	7(2)
C(13)	66(2)	78(2)	62(2)	-1(2)	-11(2)	0(2)
C(14)	55(2)	74(2)	76(2)	-5(2)	-15(2)	0(2)
C(15)	55(2)	74(2)	77(2)	-5(2)	-11(2)	1(2)
C(16)	51(2)	75(2)	74(2)	-5(2)	-7(2)	-2(2)
C(17)	55(2)	113(3)	120(4)	2(3)	4(2)	-1(2)

C(18)	60(2)	105(3)	88(2)	8(2)	-9(2)	-5(2)
C(19)	60(2)	92(3)	70(2)	-3(2)	-10(2)	-1(2)
C(20)	53(2)	107(3)	82(2)	8(2)	-7(2)	8(2)

Table 5. Hydrogen coordinates (x 10^4) and isotropic displacement parameters (A^2 x 10^3) for C20H34O2.

	x	y	z	U(eq)
H(1A)	-758	2896	8999	97
H(1B)	1444	3061	9021	97
H(2A)	-856	3236	7712	106
H(2B)	109	4049	8145	106
H(3A)	3079	3585	7768	97
H(3B)	1801	3677	7042	97
H(5)	3558	1962	8304	76
H(6A)	1887	423	7642	88
H(6B)	4024	692	7503	88
H(7A)	3778	-367	8505	84
H(7B)	4709	542	8786	84
H(9)	2414	1680	9502	76
H(11A)	-367	2030	10068	98
H(11B)	-1393	1174	9729	98
H(12A)	1166	1209	10955	98
H(12B)	-864	768	10943	98
H(13)	1515	-384	10971	82
H(14A)	3664	-502	9952	82
H(14B)	3751	534	10203	82
H(15A)	-735	-79	8969	82

H(15B)	754	-869	8852	82
H(17A)	-2744	-1167	9843	144
H(17B)	-2671	-164	10150	144
H(17C)	-2258	-983	10700	144
H(18A)	4852	1772	6748	126
H(18B)	5419	2536	7330	126
H(18C)	4585	2802	6533	126
H(19A)	1258	1342	6608	89
H(19B)	-182	2118	6820	89
H(20A)	-2055	1368	8572	121
H(20B)	-824	822	7984	121
H(20C)	-1522	1810	7788	121
H(2O)	350(70)	-1970(30)	9740(20)	97(15)
H(1O)	2200(60)	2150(30)	5720(30)	87(15)

Table 6. Torsion angles [deg] for C20H34O2.

C(10)-C(1)-C(2)-C(3)	-56.2(5)
C(1)-C(2)-C(3)-C(4)	54.7(5)
C(2)-C(3)-C(4)-C(18)	-166.8(3)
C(2)-C(3)-C(4)-C(19)	74.9(4)
C(2)-C(3)-C(4)-C(5)	-48.8(4)
C(3)-C(4)-C(5)-C(6)	-179.7(3)
C(18)-C(4)-C(5)-C(6)	-63.0(4)
C(19)-C(4)-C(5)-C(6)	57.8(4)
C(3)-C(4)-C(5)-C(10)	47.7(4)
C(18)-C(4)-C(5)-C(10)	164.4(3)
C(19)-C(4)-C(5)-C(10)	-74.8(4)
C(10)-C(5)-C(6)-C(7)	-61.9(4)
C(4)-C(5)-C(6)-C(7)	162.2(3)
C(5)-C(6)-C(7)-C(8)	55.7(4)
C(6)-C(7)-C(8)-C(14)	-166.8(3)
C(6)-C(7)-C(8)-C(9)	-47.4(4)
C(6)-C(7)-C(8)-C(15)	81.2(4)
C(7)-C(8)-C(9)-C(11)	178.9(3)
C(14)-C(8)-C(9)-C(11)	-60.7(4)
C(15)-C(8)-C(9)-C(11)	48.9(4)
C(7)-C(8)-C(9)-C(10)	47.7(4)
C(14)-C(8)-C(9)-C(10)	168.1(3)
C(15)-C(8)-C(9)-C(10)	-82.3(4)
C(2)-C(1)-C(10)-C(20)	-69.7(4)
C(2)-C(1)-C(10)-C(5)	52.0(4)

C(2)-C(1)-C(10)-C(9)	167.5(4)
C(6)-C(5)-C(10)-C(1)	176.2(3)
C(4)-C(5)-C(10)-C(1)	-49.3(4)
C(6)-C(5)-C(10)-C(20)	-64.6(4)
C(4)-C(5)-C(10)-C(20)	69.9(4)
C(6)-C(5)-C(10)-C(9)	59.4(3)
C(4)-C(5)-C(10)-C(9)	-166.1(3)
C(11)-C(9)-C(10)-C(1)	60.3(4)
C(8)-C(9)-C(10)-C(1)	-170.1(3)
C(11)-C(9)-C(10)-C(20)	-59.7(4)
C(8)-C(9)-C(10)-C(20)	69.9(4)
C(11)-C(9)-C(10)-C(5)	176.8(3)
C(8)-C(9)-C(10)-C(5)	-53.7(4)
C(8)-C(9)-C(11)-C(12)	42.6(4)
C(10)-C(9)-C(11)-C(12)	174.9(3)
C(9)-C(11)-C(12)-C(13)	-40.9(5)
C(11)-C(12)-C(13)-C(14)	55.9(4)
C(11)-C(12)-C(13)-C(16)	-56.4(5)
C(16)-C(13)-C(14)-C(8)	51.1(3)
C(12)-C(13)-C(14)-C(8)	-70.2(3)
C(7)-C(8)-C(14)-C(13)	-165.2(3)
C(9)-C(8)-C(14)-C(13)	74.0(3)
C(15)-C(8)-C(14)-C(13)	-43.8(3)
C(7)-C(8)-C(15)-C(16)	138.7(3)
C(14)-C(8)-C(15)-C(16)	21.0(3)
C(9)-C(8)-C(15)-C(16)	-93.7(3)
C(14)-C(13)-C(16)-O(2)	78.6(3)
C(12)-C(13)-C(16)-O(2)	-164.9(3)
C(14)-C(13)-C(16)-C(17)	-162.0(4)

C(12)-C(13)-C(16)-C(17)	-45.5(5)
C(14)-C(13)-C(16)-C(15)	-36.6(3)
C(12)-C(13)-C(16)-C(15)	79.8(4)
C(8)-C(15)-C(16)-O(2)	-103.7(3)
C(8)-C(15)-C(16)-C(13)	9.4(4)
C(8)-C(15)-C(16)-C(17)	135.7(3)
C(3)-C(4)-C(19)-O(1)	71.9(4)
C(18)-C(4)-C(19)-O(1)	-45.3(5)
C(5)-C(4)-C(19)-O(1)	-166.4(3)

Symmetry transformations used to generate equivalent atoms:

Table 7. Hydrogen bonds for C20H34O2 [A and deg.].

D-H...A	d(D-H)	d(H...A)	d(D...A)	<(DHA)
O(1)-H(1O)...O(2)#1	0.77(4)	1.97(4)	2.736(4)	173(4)
O(2)-H(2O)...O(1)#2	0.86(4)	1.94(5)	2.770(4)	161(4)

Symmetry transformations used to generate equivalent atoms:

#1 -x+1/2,-y,z-1/2 #2 -x,y-1/2,-z+3/2

Institution:
Protocol: 202_00010650 513.PRO
Listmode Replay: New Protocol
Analysis Date: 03-May-2012, 13:06:47
Settings File: 00Ciclo Celular de Javi.PRO, 03-May-2012, 10:53:20
Listmode File: 01_00010676 539.LMD

Run Date: 03-May-12, 11:51:48
Sample ID: Well 01
User ID: anajavi
Acquisition Time/Events: 58.8s / 10000 (UNKNOWN)

Instrument SN: AL01002 Software Version: CXP v2.2

(F1)[Ungated] 01_00010676 539.LMD : FS Lin/SS Lin - ADC

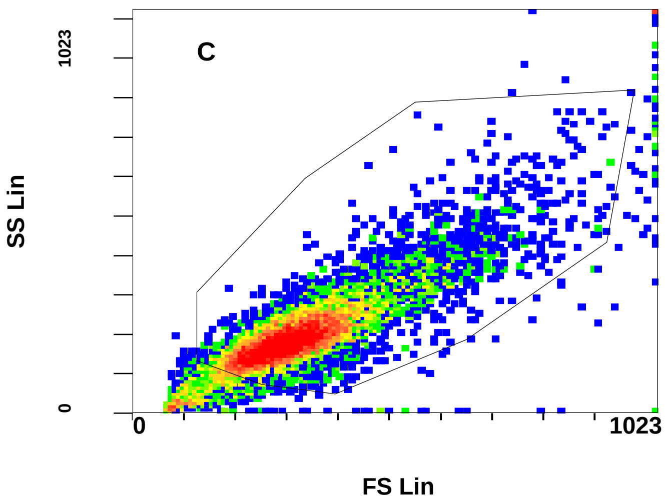

(F1)[Ungated] 01_00010676 539.LMD : FL3 Lin/AUX - ADC

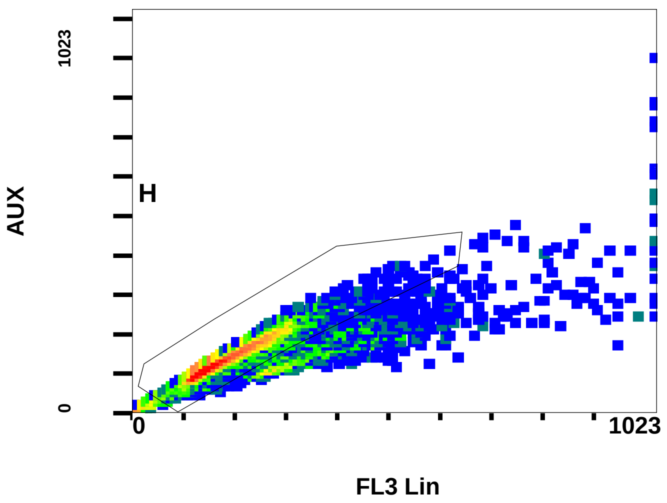

(F1)[H] 01_00010676 539.LMD : FL3 Lin - ADC

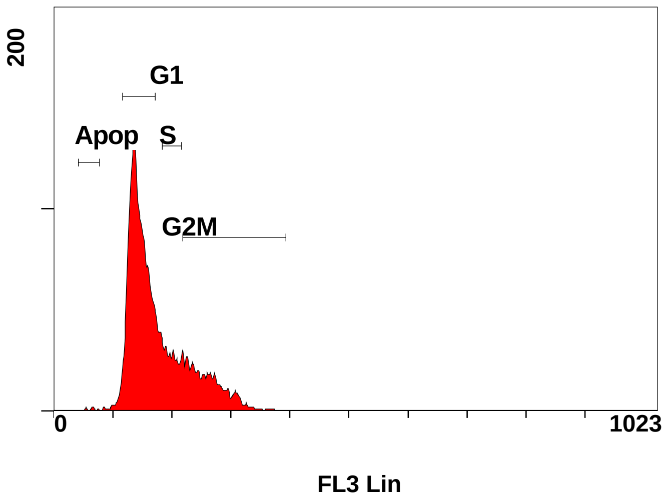

(F1)[H] 01_00010676 539.LMD : FL3 Lin/SS Lin - ADC

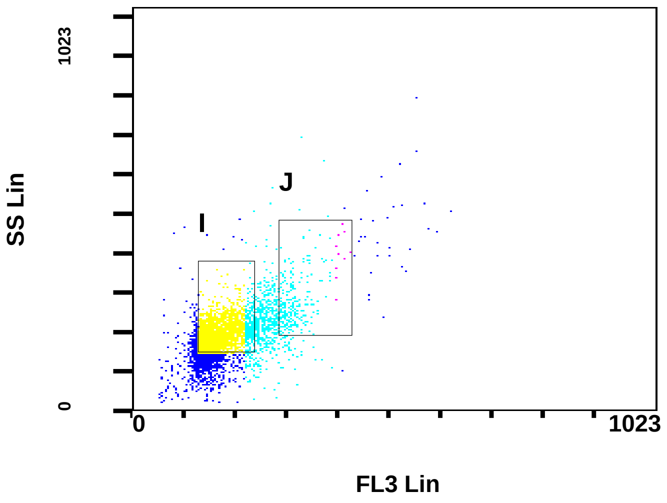

Statistical Analysis

PROGRAM INFORMATION

File:- 01_00010676 539.LMD						
Gate:- H [H]						
Compensation:- Advanced						
Filename:- 01_00010676 539.LMD						
Mean Calculation Method:-LOG-LOG						
Region	Number	%Total	%Gated	X-Mean	X-HPCV	Y-Mean
ALL	8499	84.99	100.00	181	8.13	###
ALL	8499	84.99	100.00	181	8.13	189
Apop	68	0.68	0.80	62.9	2.25	###
G1	4704	47.04	55.35	143	8.13	###
G2M	1943	19.43	22.86	266	0.56	###
I	4729	47.29	55.64	168	8.47	195
J	469	4.69	5.52	320	0.24	284
S	968	9.68	11.39	199	1.85	###

File:- 01_00010676 539.LMD						
Gate:- Ungated						
Compensation:- Advanced						
Filename:- 01_00010676 539.LMD						
Mean Calculation Method:-LOG-LOG						
Region	Number	%Total	%Gated	X-Mean	X-HPCV	Y-Mean
ALL	10000	100.00	100.00	195	8.13	132
ALL	10000	100.00	100.00	333	10.51	202
C	9083	90.83	90.83	344	10.53	210
H	8499	84.99	84.99	181	8.13	135

Institution:

Protocol: 202_00010650 513.PRO

Listmode Replay: New Protocol

Analysis Date: 03-May-2012, 13:06:47

Settings File: 00Ciclo Celular de Javi.PRO, 03-May-2012, 10:53:20

Listmode File: 01_00010676 539.LMD

Run Date: 03-May-12, 11:51:48

Sample ID: Well 01

User ID: anajavi

Acquisition Time/Events: 58.8s / 10000 (UNKNOWN)

Tube ID: NoRead

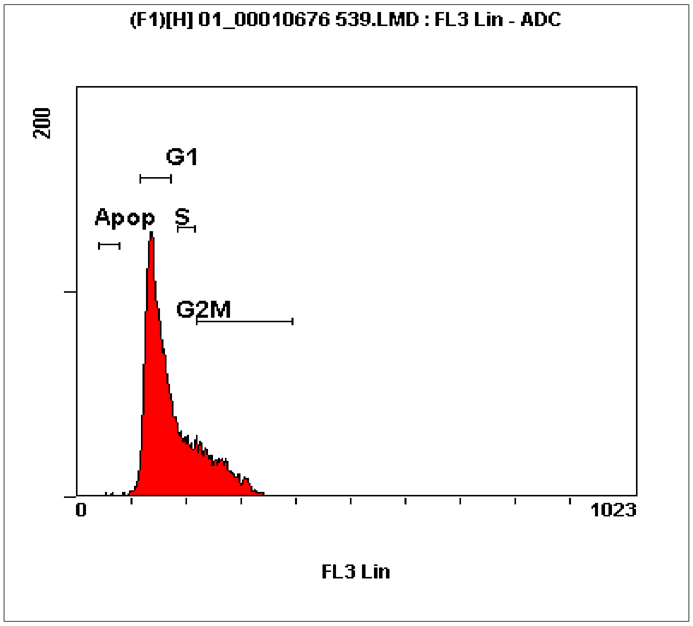

(F1)[H] 01_00010676 539.LMD : FL3 Lin

Region	Number	%Total	%Gated	X-Mean	HP X-CV	Y-Mea
ALL	8499	84.99	100.00	181	8.13	###
Apop	68	0.68	0.80	62.9	2.25	###
G1	4704	47.04	55.35	143	8.13	###
G2M	1943	19.43	22.86	266	0.56	###
S	968	9.68	11.39	199	1.85	###

Institution:
Protocol: 202_00010650 513.PRO
Listmode Replay: New Protocol
Analysis Date: 03-May-2012, 13:07:30
Settings File: 00Ciclo Celular de Javi.PRO, 03-May-2012, 10:53:20
Listmode File: 02_00010677 540.LMD

Run Date: 03-May-12, 11:53:22
Sample ID: Well 02
User ID: anajavi
Acquisition Time/Events: 78.1s / 10000 (UNKNOWN)

Instrument SN: AL01002 Software Version: CXP v2.2

(F1)[Ungated] 02_00010677 540.LMD : FS Lin/SS Lin - ADC

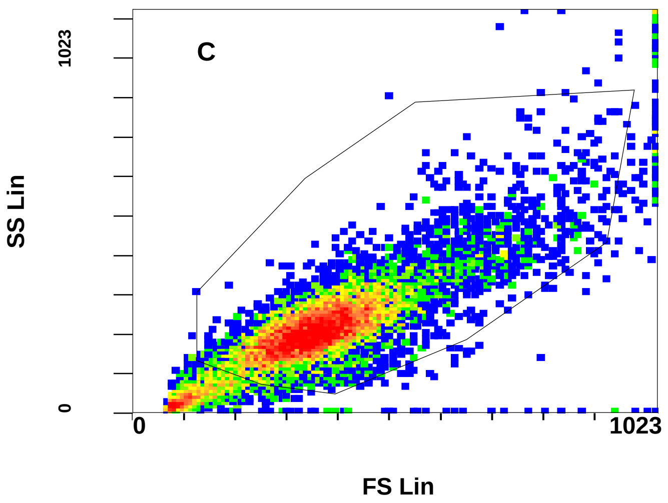

(F1)[Ungated] 02_00010677 540.LMD : FL3 Lin/AUX - ADC

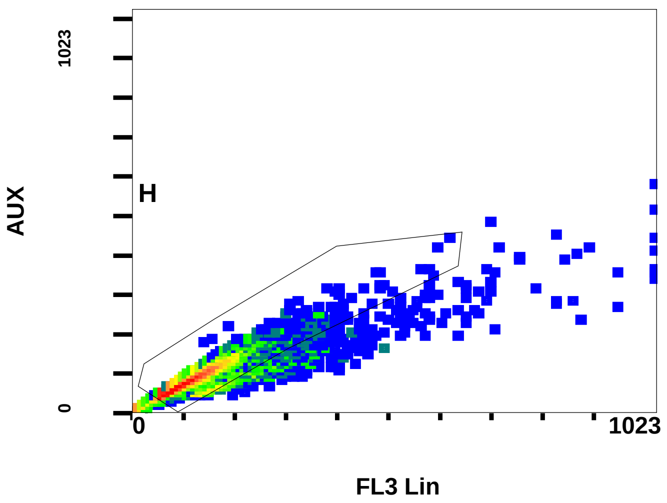

(F1)[H] 02_00010677 540.LMD : FL3 Lin - ADC

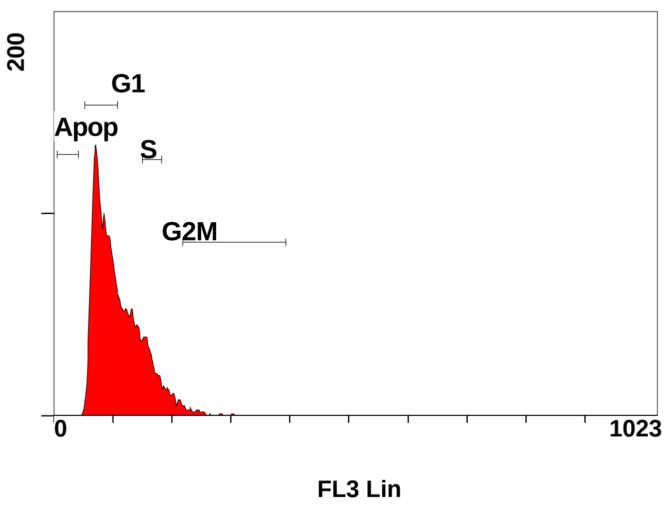

(F1)[H] 02_00010677 540.LMD : FL3 Lin/SS Lin - ADC

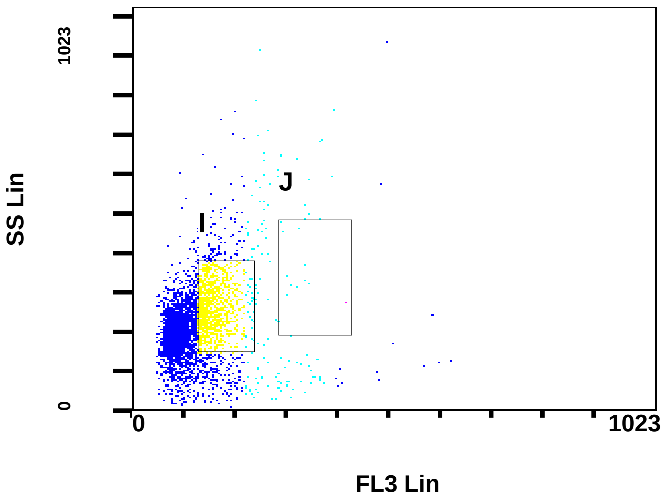

Statistical Analysis

PROGRAM INFORMATION

File:- 02_00010677 540.LMD						
Gate:- H [H]						
Compensation:- Advanced						
Filename:- 02_00010677 540.LMD						
Mean Calculation Method:-LOG-LOG						
Region	Number	%Total	%Gated	X-Mean	X-HPCV	Y-Mean
ALL	8766	87.66	100.00	115	20.83	###
ALL	8766	87.66	100.00	115	20.83	218
Apop	0	0.00	0.00	0	0.00	###
G1	4829	48.29	55.09	80.9	20.83	###
G2M	319	3.19	3.64	271	0.54	###
I	1959	19.59	22.35	159	6.18	261
J	20	0.20	0.23	331	0.14	337
S	1006	10.06	11.48	164	5.95	###

File:- 02_00010677 540.LMD						
Gate:- Ungated						
Compensation:- Advanced						
Filename:- 02_00010677 540.LMD						
Mean Calculation Method:-LOG-LOG						
Region	Number	%Total	%Gated	X-Mean	X-HPCV	Y-Mean
ALL	10000	100.00	100.00	370	0.00	215
ALL	10000	100.00	100.00	117	###	79.9
C	8769	87.69	87.69	390	9.87	230
H	8766	87.66	87.66	115	20.83	83.3

Institution:

Protocol: 202_00010650 513.PRO

Listmode Replay: New Protocol

Analysis Date: 03-May-2012, 13:07:31

Settings File: 00Ciclo Celular de Javi.PRO, 03-May-2012, 10:53:20

Listmode File: 02_00010677 540.LMD

Run Date: 03-May-12, 11:53:22

Sample ID: Well 02

User ID: anajavi

Acquisition Time/Events: 78.1s / 10000 (UNKNOWN)

Tube ID: NoRead

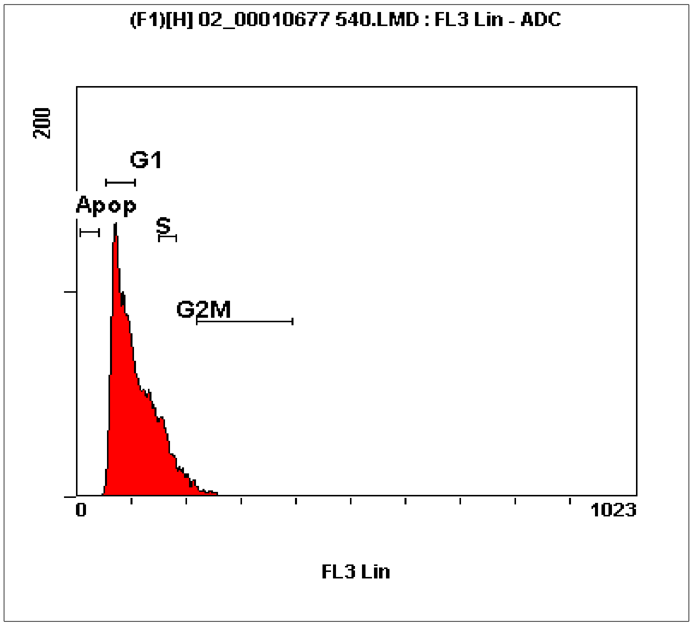

(F1)[H] 02_00010677 540.LMD : FL3 Lin						
Region	Number	%Total	%Gated	X-Mean	HP X-CV	Y-Mea
ALL	8766	87.66	100.00	115	20.83	###
Apop	0	0.00	0.00	0	0.00	###
G1	4829	48.29	55.09	80.9	20.83	###
G2M	319	3.19	3.64	271	0.54	###
S	1006	10.06	11.48	164	5.95	###

Institution:
Protocol: 202_00010650 513.PRO
Listmode Replay: New Protocol
Analysis Date: 03-May-2012, 13:20:43
Settings File: 00Ciclo Celular de Javi.PRO, 03-May-2012, 10:53:20
Listmode File: 21_00010670 533.LMD

Run Date: 03-May-12, 11:32:28
Sample ID: Well 21
User ID: anajavi
Acquisition Time/Events: 65.9s / 10000 (UNKNOWN)

Instrument SN: AL01002 Software Version: CXP v2.2

(F1)[Ungated] 21_00010670 533.LMD : FS Lin/SS Lin - ADC

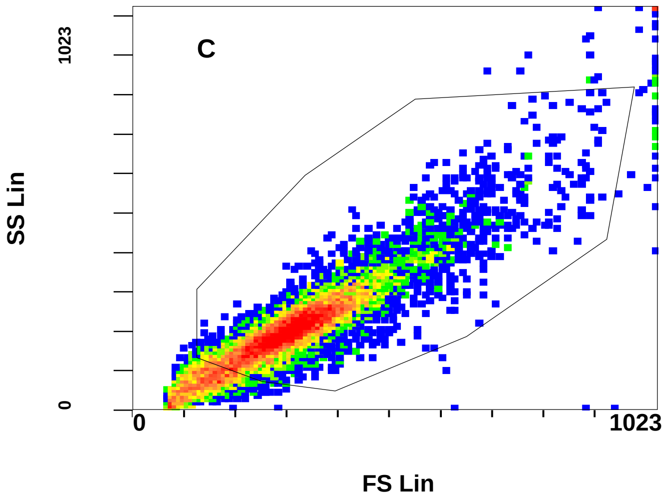

(F1)[Ungated] 21_00010670 533.LMD : FL3 Lin/AUX - ADC

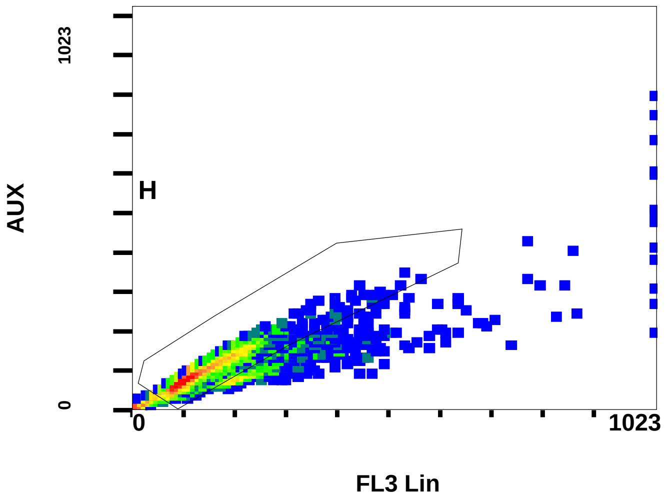

(F1)[H] 21_00010670 533.LMD : FL3 Lin - ADC

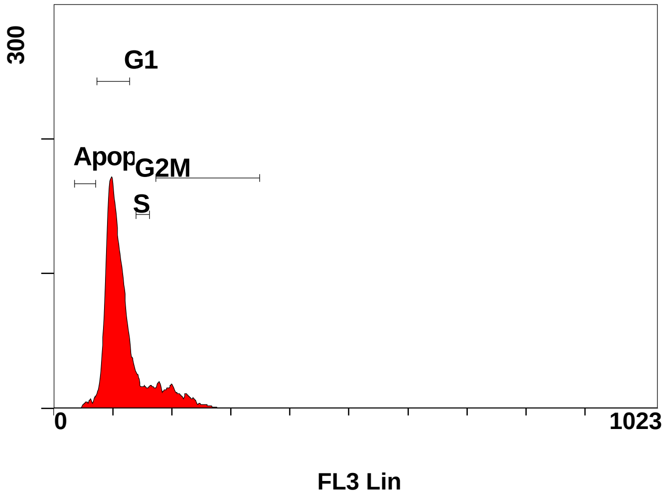

(F1)[H] 21_00010670 533.LMD : FL3 Lin/SS Lin - ADC

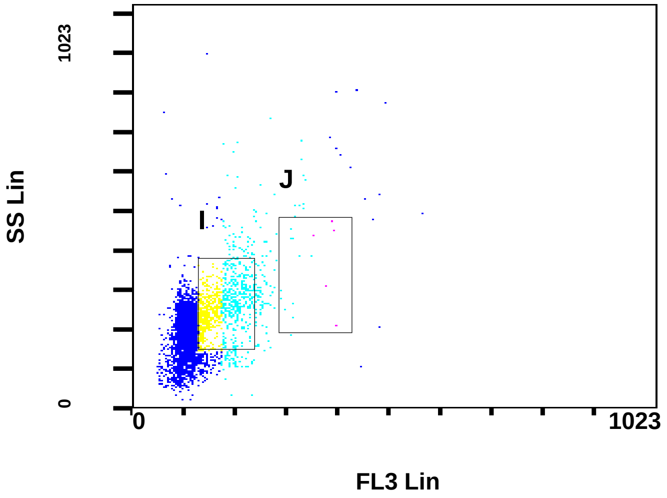

Statistical Analysis

PROGRAM INFORMATION

File:- 21_00010670 533.LMD

Gate:- H [H]

Compensation:- Advanced

Filename:- 21_00010670 533.LMD

Mean Calculation Method:-LOG-LOG

Region	Number	%Total	%Gated	X-Mean	X-HPCV	Y-Mean
ALL	8076	80.76	100.00	124	11.80	###
ALL	8076	80.76	100.00	124	11.80	207
Apop	171	1.71	2.12	60.9	3.08	###
G1	5688	56.88	70.43	103	11.80	###
G2M	1149	11.49	14.23	213	2.18	###
I	1653	16.53	20.47	171	0.65	256
J	34	0.34	0.42	331	0.14	346
S	478	4.78	5.92	149	2.15	###

File:- 21_00010670 533.LMD

Gate:- Ungated

Compensation:- Advanced

Filename:- 21_00010670 533.LMD

Mean Calculation Method:-LOG-LOG

Region	Number	%Total	%Gated	X-Mean	X-HPCV	Y-Mean
ALL	10000	100.00	100.00	310	5.53	204
ALL	10000	100.00	100.00	120	###	81.2
C	8284	82.84	82.84	338	5.51	226
H	8076	80.76	80.76	124	11.80	89.9

Institution:

Run Date: 03-May-12, 11:32:28

Protocol: 202_00010650 513.PRO

Sample ID: Well 21

Listmode Replay: New Protocol

User ID: anajavi

Analysis Date: 03-May-2012, 13:20:43

Acquisition Time/Events: 65.9s / 10000 (UNKNOWN)

Settings File: 00Ciclo Celular de Javi.PRO, 03-May-2012, 10:53:20

Tube ID: NoRead

Listmode File: 21_00010670 533.LMD

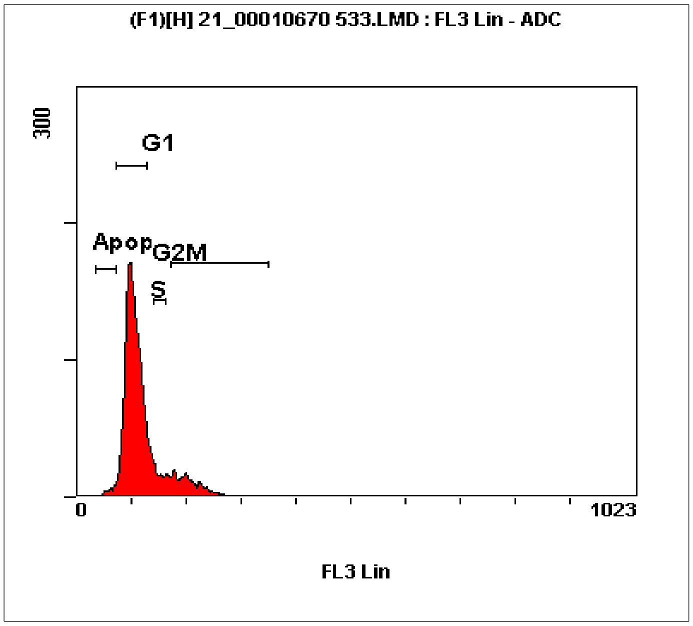

(F1)[H] 21_00010670 533.LMD : FL3 Lin

Region	Number	%Total	%Gated	X-Mean	HP X-CV	Y-Mea
ALL	8076	80.76	100.00	124	11.80	###
Apop	171	1.71	2.12	60.9	3.08	###
G1	5688	56.88	70.43	103	11.80	###
G2M	1149	11.49	14.23	213	2.18	###
S	478	4.78	5.92	149	2.15	###

Institution:

Protocol: 202_00010650 513.PRO

Listmode Replay: New Protocol

Analysis Date: 03-May-2012, 13:21:18

Settings File: 00Ciclo Celular de Javi.PRO, 03-May-2012, 10:53:20

Listmode File: 22_00010671 534.LMD

Run Date: 03-May-12, 11:34:10

Sample ID: Well 22

User ID: anajavi

Acquisition Time/Events: 78.1s / 10000 (UNKNOWN)

Instrument SN: AL01002 Software Version: CXP v2.2

(F1)[Ungated] 22_00010671 534.LMD : FS Lin/SS Lin - ADC

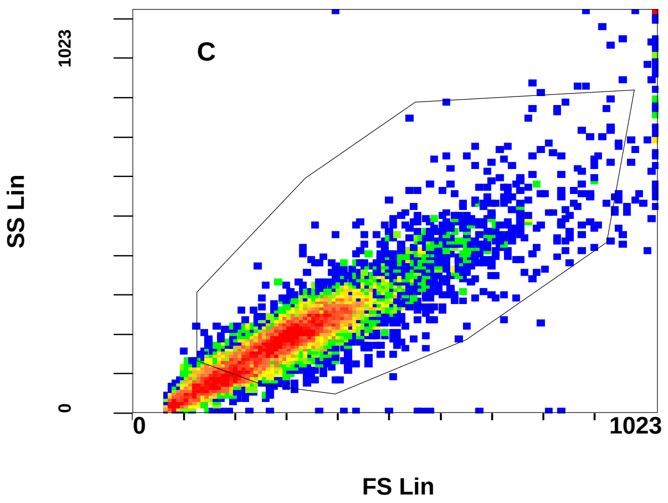

(F1)[Ungated] 22_00010671 534.LMD : FL3 Lin/AUX - ADC

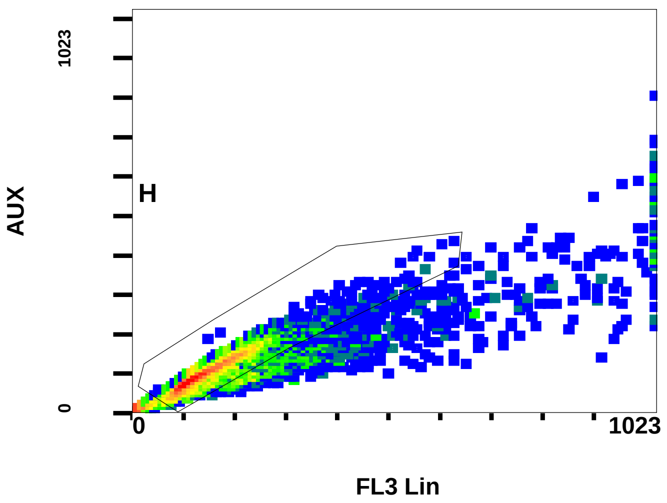

(F1)[H] 22_00010671 534.LMD : FL3 Lin - ADC

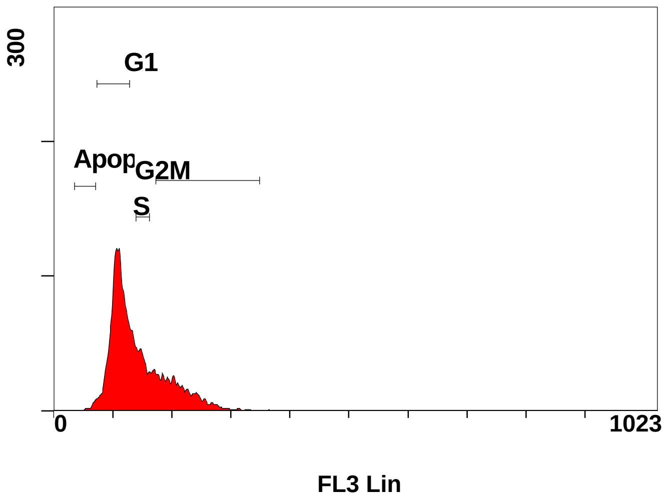

(F1)[H] 22_00010671 534.LMD : FL3 Lin/SS Lin - ADC

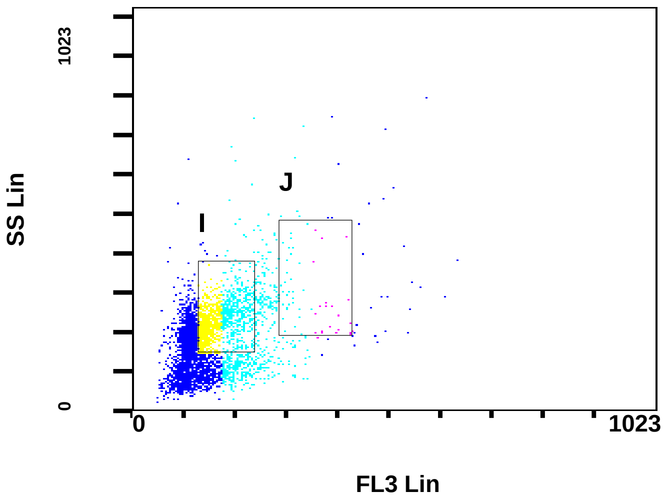

Statistical Analysis

PROGRAM INFORMATION

File:- 22_00010671 534.LMD

Gate:- H [H]

Compensation:- Advanced

Filename:- 22_00010671 534.LMD

Mean Calculation Method:-LOG-LOG

Region	Number	%Total	%Gated	X-Mean	X-HPCV	Y-Mean
ALL	7868	78.68	100.00	150	10.93	###
ALL	7868	78.68	100.00	150	10.93	183
Apop	115	1.15	1.46	62.7	4.82	###
G1	3758	37.58	47.76	107	10.93	###
G2M	1993	19.93	25.33	220	1.75	###
I	2240	22.40	28.47	171	4.50	233
J	122	1.22	1.55	339	0.44	294
S	980	9.80	12.46	149	4.61	###

File:- 22_00010671 534.LMD

Gate:- Ungated

Compensation:- Advanced

Filename:- 22_00010671 534.LMD

Mean Calculation Method:-LOG-LOG

Region	Number	%Total	%Gated	X-Mean	X-HPCV	Y-Mean
ALL	10000	100.00	100.00	301	0.00	187
ALL	10000	100.00	100.00	160	###	100
C	7513	75.13	75.13	342	5.81	218
H	7868	78.68	78.68	150	10.93	105

Institution:

Protocol: 202_00010650 513.PRO

Listmode Replay: New Protocol

Analysis Date: 03-May-2012, 13:21:18

Settings File: 00Ciclo Celular de Javi.PRO, 03-May-2012, 10:53:20

Listmode File: 22_00010671 534.LMD

Run Date: 03-May-12, 11:34:10

Sample ID: Well 22

User ID: anajavi

Acquisition Time/Events: 78.1s / 10000 (UNKNOWN)

Tube ID: NoRead

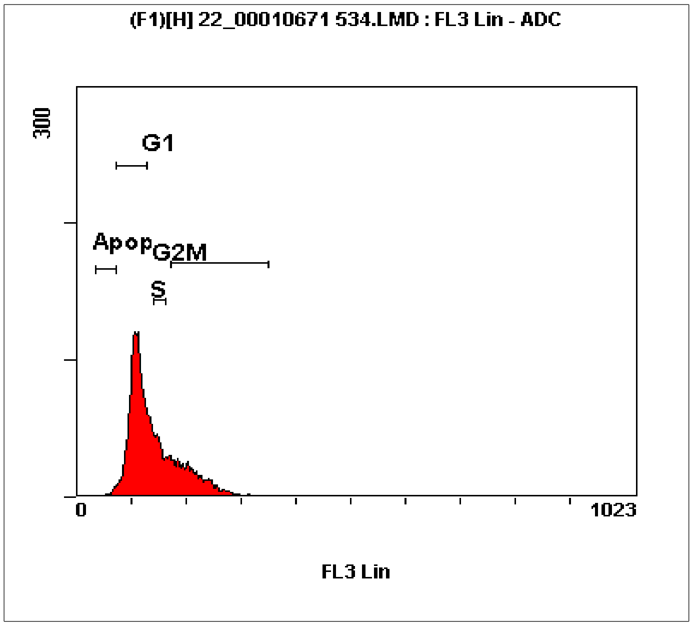

(F1)[H] 22_00010671 534.LMD : FL3 Lin

Region	Number	%Total	%Gated	X-Mean	HP X-CV	Y-Mea
ALL	7868	78.68	100.00	150	10.93	###
Apop	115	1.15	1.46	62.7	4.82	###
G1	3758	37.58	47.76	107	10.93	###
G2M	1993	19.93	25.33	220	1.75	###
S	980	9.80	12.46	149	4.61	###

Institution:

Protocol: 202_00010650 513.PRO

Listmode Replay: New Protocol

Analysis Date: 03-May-2012, 13:22:03

Settings File: 00Ciclo Celular de Javi.PRO, 03-May-2012, 10:53:20

Listmode File: 23_00010672 535.LMD

Run Date: 03-May-12, 11:36:04

Sample ID: Well 23

User ID: anajavi

Acquisition Time/Events: 110.2s / 10000 (UNKNOWN)

Instrument SN: AL01002 Software Version: CXP v2.2

(F1)[Ungated] 23_00010672 535.LMD : FS Lin/SS Lin - ADC

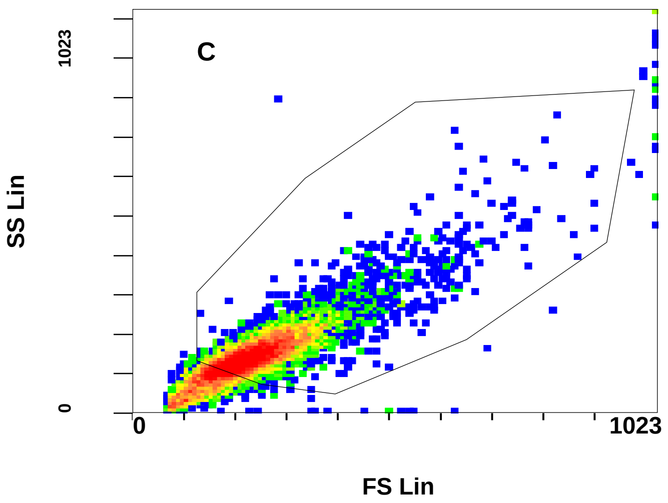

(F1)[Ungated] 23_00010672 535.LMD : FL3 Lin/AUX - ADC

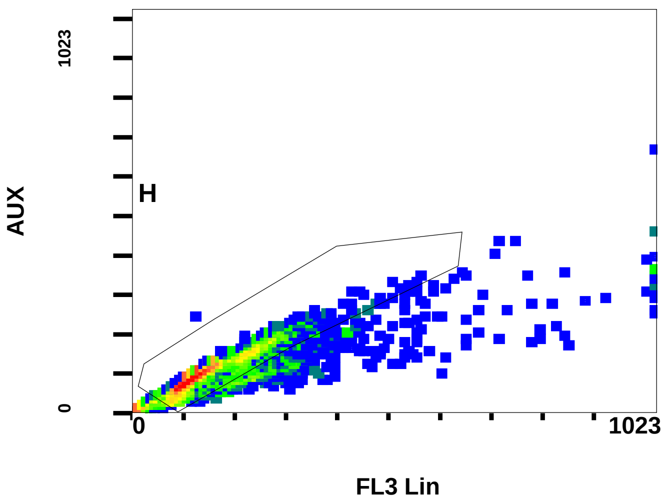

(F1)[H] 23_00010672 535.LMD : FL3 Lin - ADC

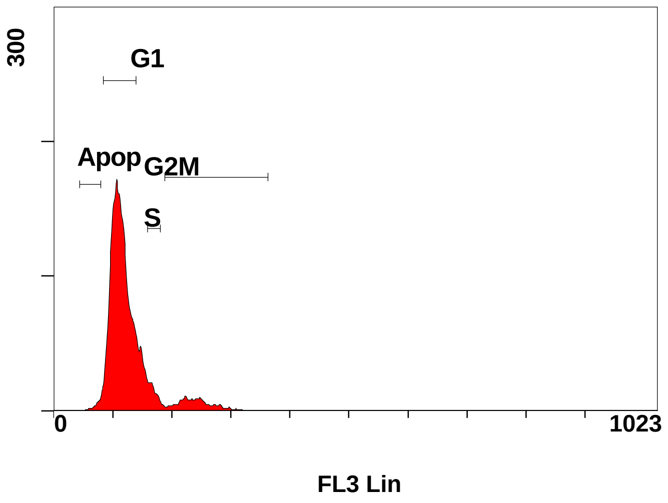

(F1)[H] 23_00010672 535.LMD : FL3 Lin/SS Lin - ADC

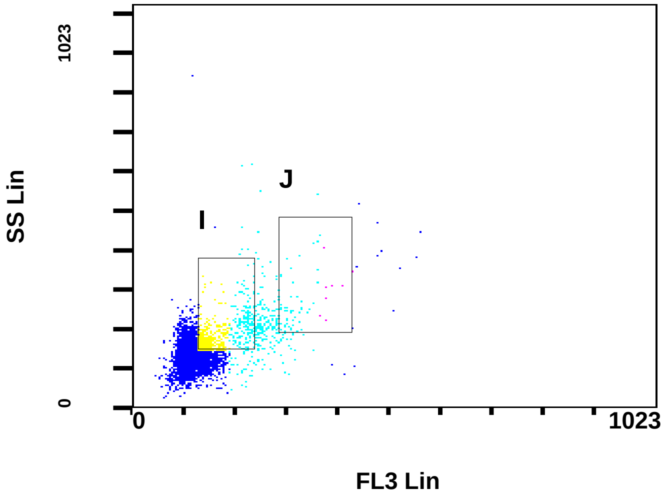

Statistical Analysis

PROGRAM INFORMATION

File:- 23_00010672 535.LMD						
Gate:- H [H]						
Compensation:- Advanced						
Filename:- 23_00010672 535.LMD						
Mean Calculation Method:-LOG-LOG						
Region	Number	%Total	%Gated	X-Mean	X-HPCV	Y-Mean
ALL	8357	83.57	100.00	133	11.04	###
ALL	8357	83.57	100.00	133	11.04	148
Apop	147	1.47	1.76	68.5	0.69	###
G1	5981	59.81	71.57	111	11.04	###
G2M	919	9.19	11.00	249	0.76	###
I	1175	11.75	14.06	168	4.91	189
J	131	1.31	1.57	319	0.25	255
S	412	4.12	4.93	167	2.94	###

File:- 23_00010672 535.LMD						
Gate:- Ungated						
Compensation:- Advanced						
Filename:- 23_00010672 535.LMD						
Mean Calculation Method:-LOG-LOG						
Region	Number	%Total	%Gated	X-Mean	X-HPCV	Y-Mean
ALL	10000	100.00	100.00	238	17.66	146
ALL	10000	100.00	100.00	129	156.26	87.4
C	7675	76.75	76.75	263	16.68	165
H	8357	83.57	83.57	133	11.04	95.7

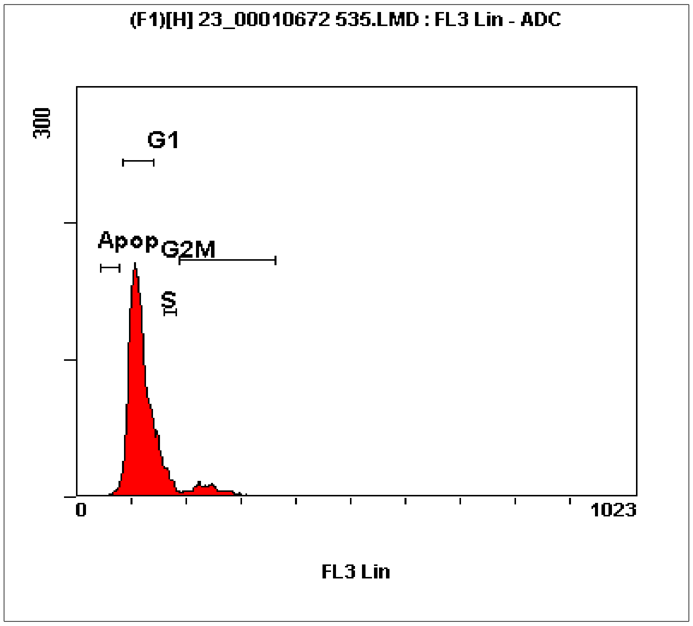

(F1)[H] 23_00010672 535.LMD : FL3 Lin						
Region	Number	%Total	%Gated	X-Mean	HP X-CV	Y-Mea
ALL	8357	83.57	100.00	133	11.04	###
Apop	147	1.47	1.76	68.5	0.69	###
G1	5981	59.81	71.57	111	11.04	###
G2M	919	9.19	11.00	249	0.76	###
S	412	4.12	4.93	167	2.94	###

Institution:
Protocol: 202_00010650 513.PRO
Listmode Replay: New Protocol
Analysis Date: 03-May-2012, 13:22:28
Settings File: 00Ciclo Celular de Javi.PRO, 03-May-2012, 10:53:20
Listmode File: 25_00010673 536.LMD

Run Date: 03-May-12, 11:38:30
Sample ID: Well 25
User ID: anajavi
Acquisition Time/Events: 145.2s / 10000 (UNKNOWN)

Instrument SN: AL01002 Software Version: CXP v2.2

(F1)[Ungated] 25_00010673 536.LMD : FS Lin/SS Lin - ADC

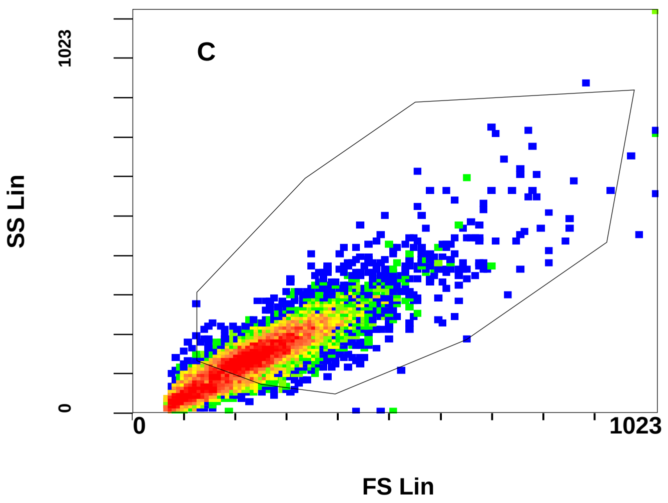

(F1)[Ungated] 25_00010673 536.LMD : FL3 Lin/AUX - ADC

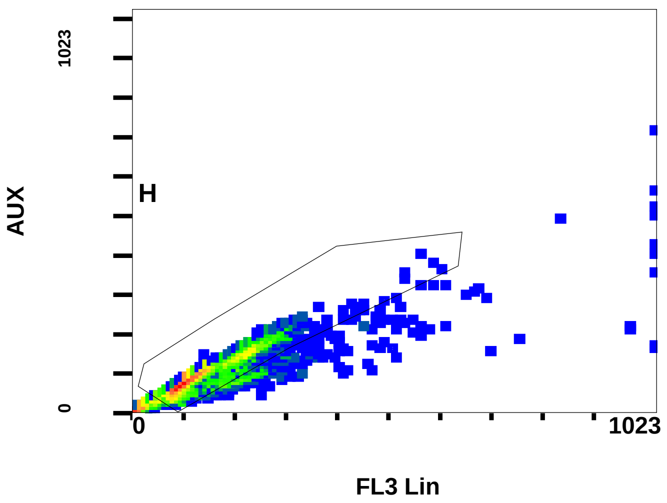

(F1)[H] 25_00010673 536.LMD : FL3 Lin - ADC

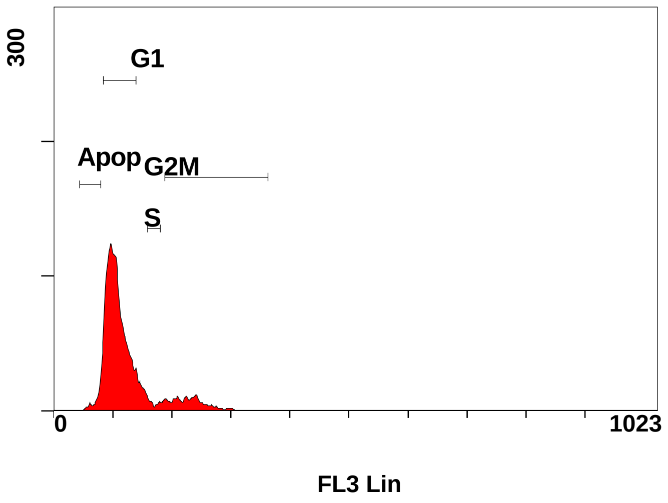

(F1)[H] 25_00010673 536.LMD : FL3 Lin/SS Lin - ADC

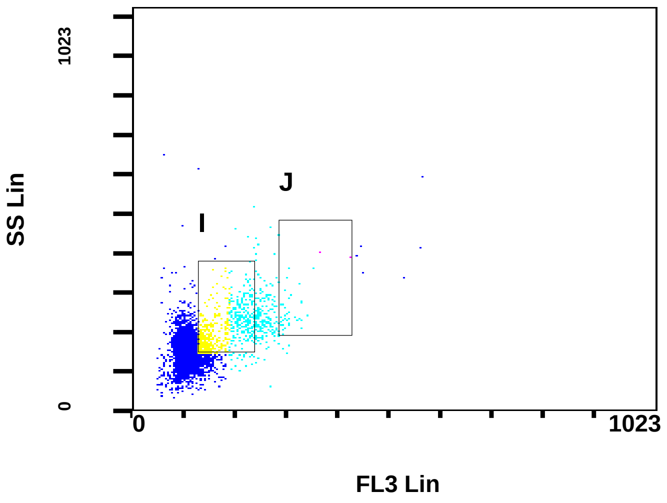

Statistical Analysis

PROGRAM INFORMATION

File:- 25_00010673 536.LMD						
Gate:- H [H]						
Compensation:- Advanced						
Filename:- 25_00010673 536.LMD						
Mean Calculation Method:-LOG-LOG						
Region	Number	%Total	%Gated	X-Mean	X-HPCV	Y-Mean
ALL	6394	63.94	100.00	129	11.59	###
ALL	6394	63.94	100.00	129	11.59	167
Apop	249	2.49	3.89	68.5	2.62	###
G1	4415	44.15	69.05	106	11.59	###
G2M	936	9.36	14.64	238	0.98	###
I	1150	11.50	17.99	177	4.88	213
J	96	0.96	1.50	310	0.25	262
S	183	1.83	2.86	168	1.94	###

File:- 25_00010673 536.LMD						
Gate:- Ungated						
Compensation:- Advanced						
Filename:- 25_00010673 536.LMD						
Mean Calculation Method:-LOG-LOG						
Region	Number	%Total	%Gated	X-Mean	X-HPCV	Y-Mean
ALL	10000	100.00	100.00	240	6.33	142
ALL	10000	100.00	100.00	94.7	139.60	64.8
C	6797	67.97	67.97	288	12.25	178
H	6394	63.94	63.94	129	11.59	92.3

Institution:

Protocol: 202_00010650 513.PRO

Listmode Replay: New Protocol

Analysis Date: 03-May-2012, 13:22:29

Settings File: 00Ciclo Celular de Javi.PRO, 03-May-2012, 10:53:20

Listmode File: 25_00010673 536.LMD

Run Date: 03-May-12, 11:38:30

Sample ID: Well 25

User ID: anajavi

Acquisition Time/Events: 145.2s / 10000 (UNKNOWN)

Tube ID: NoRead

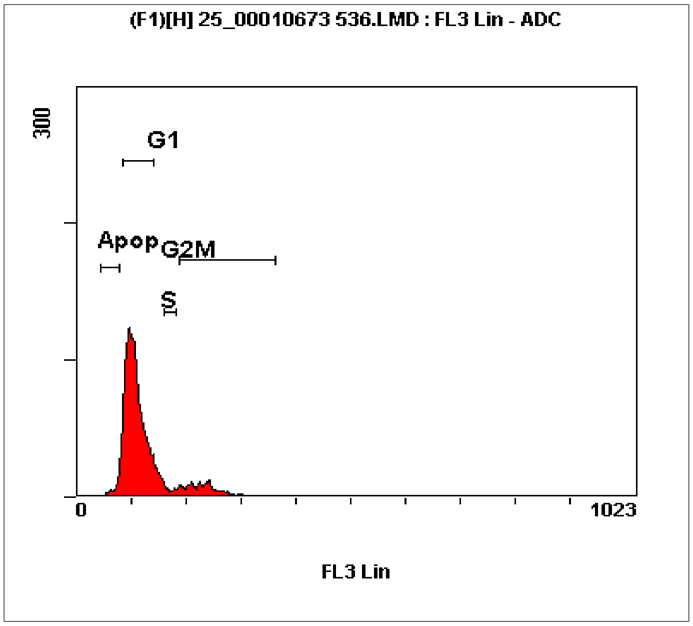

(F1)[H] 25_00010673 536.LMD : FL3 Lin

Region	Number	%Total	%Gated	X-Mean	HP X-CV	Y-Mea
ALL	6394	63.94	100.00	129	11.59	###
Apop	249	2.49	3.89	68.5	2.62	###
G1	4415	44.15	69.05	106	11.59	###
G2M	936	9.36	14.64	238	0.98	###
S	183	1.83	2.86	168	1.94	###

Institution:
Protocol: 202_00010650 513.PRO
Listmode Replay: New Protocol
Analysis Date: 03-May-2012, 13:23:03
Settings File: 00Ciclo Celular de Javi.PRO, 03-May-2012, 10:53:20
Listmode File: 26_00010674 537.LMD

Run Date: 03-May-12, 11:41:32
Sample ID: Well 26
User ID: anajavi
Acquisition Time/Events: 257.5s / 10000 (UNKNOWN)

Instrument SN: AL01002 Software Version: CXP v2.2

(F1)[Ungated] 26_00010674 537.LMD : FS Lin/SS Lin - ADC

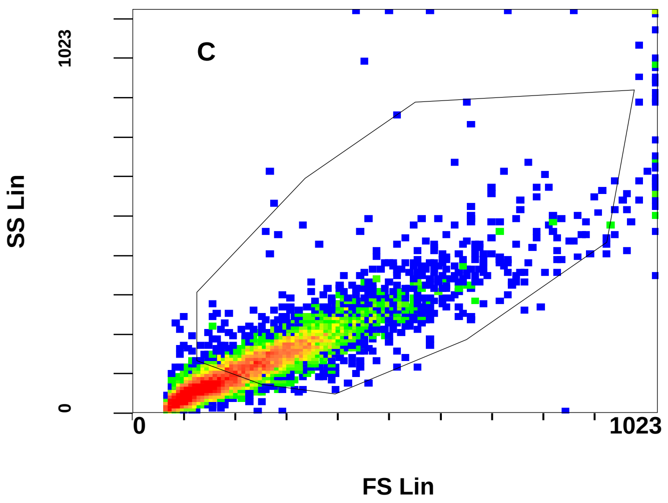

(F1)[Ungated] 26_00010674 537.LMD : FL3 Lin/AUX - ADC

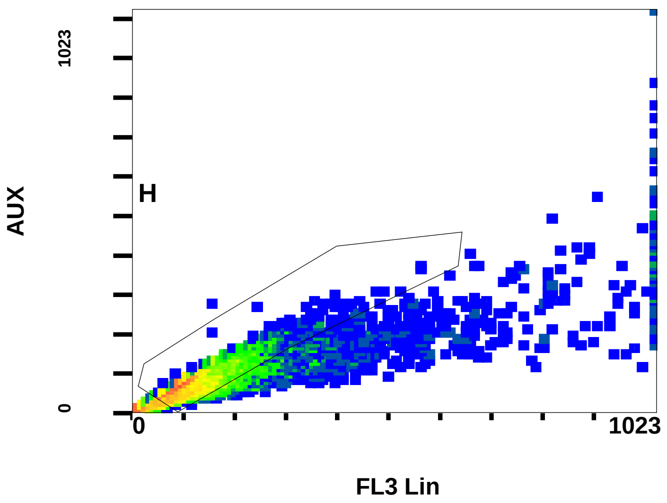

(F1)[H] 26_00010674 537.LMD : FL3 Lin - ADC

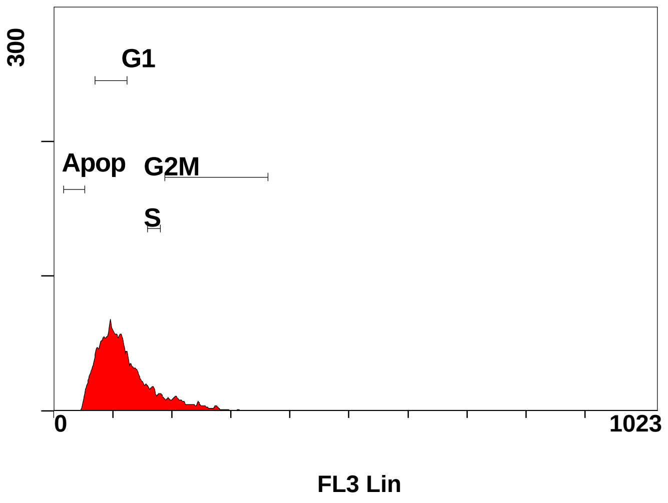

(F1)[H] 26_00010674 537.LMD : FL3 Lin/SS Lin - ADC

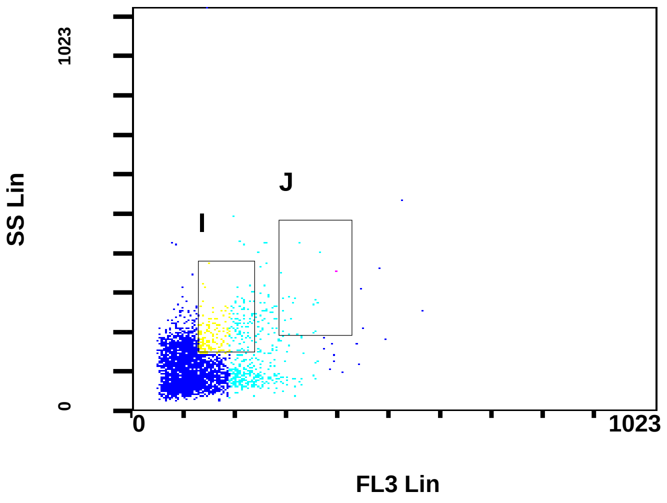

Statistical Analysis

PROGRAM INFORMATION

File:- 26_00010674 537.LMD						
Gate:- H [H]						
Compensation:- Advanced						
Filename:- 26_00010674 537.LMD						
Mean Calculation Method:-LOG-LOG						
Region	Number	%Total	%Gated	X-Mean	X-HPCV	Y-Mean
ALL	5899	58.99	100.00	127	19.81	###
ALL	5899	58.99	100.00	127	19.81	120
Apop	52	0.52	0.88	50	2.20	###
G1	3102	31.02	52.59	96.8	19.81	###
G2M	779	7.79	13.21	238	1.78	###
I	436	4.36	7.39	174	1.04	202
J	52	0.52	0.88	325	0.19	254
S	395	3.95	6.70	168	3.52	###

File:- 26_00010674 537.LMD						
Gate:- Ungated						
Compensation:- Advanced						
Filename:- 26_00010674 537.LMD						
Mean Calculation Method:-LOG-LOG						
Region	Number	%Total	%Gated	X-Mean	X-HPCV	Y-Mean
ALL	10000	100.00	100.00	219	32.63	113
ALL	10000	100.00	100.00	124	###	66
C	4529	45.29	45.29	313	0.86	170
H	5899	58.99	58.99	127	19.81	79.5

Institution:

Run Date: 03-May-12, 11:41:32

Protocol: 202_00010650 513.PRO

Sample ID: Well 26

Listmode Replay: New Protocol

User ID: anajavi

Analysis Date: 03-May-2012, 13:23:04

Acquisition Time/Events: 257.5s / 10000 (UNKNOWN)

Settings File: 00Ciclo Celular de Javi.PRO, 03-May-2012, 10:53:20

Tube ID: NoRead

Listmode File: 26_00010674 537.LMD

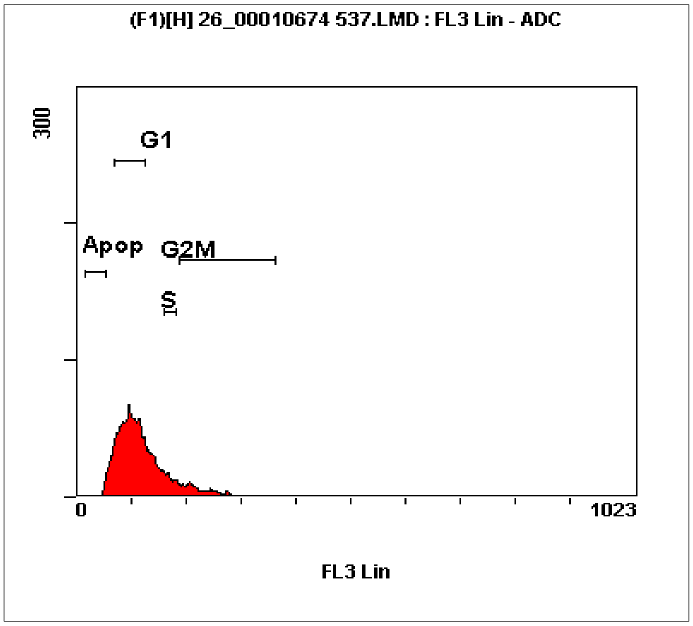

(F1)[H] 26_00010674 537.LMD : FL3 Lin

Region	Number	%Total	%Gated	X-Mean	HP X-CV	Y-Mea
ALL	5899	58.99	100.00	127	19.81	###
Apop	52	0.52	0.88	50	2.20	###
G1	3102	31.02	52.59	96.8	19.81	###
G2M	779	7.79	13.21	238	1.78	###
S	395	3.95	6.70	168	3.52	###

Institution:
Protocol: 202_00010650 513.PRO
Listmode Replay: New Protocol
Analysis Date: 03-May-2012, 13:23:36
Settings File: 00Ciclo Celular de Javi.PRO, 03-May-2012, 10:53:20
Listmode File: 27_00010675 538.LMD

Run Date: 03-May-12, 11:46:25
Sample ID: Well 27
User ID: anajavi
Acquisition Time/Events: 117.9s / 10000 (UNKNOWN)

Instrument SN: AL01002 Software Version: CXP v2.2

(F1)[Ungated] 27_00010675 538.LMD : FS Lin/SS Lin - ADC

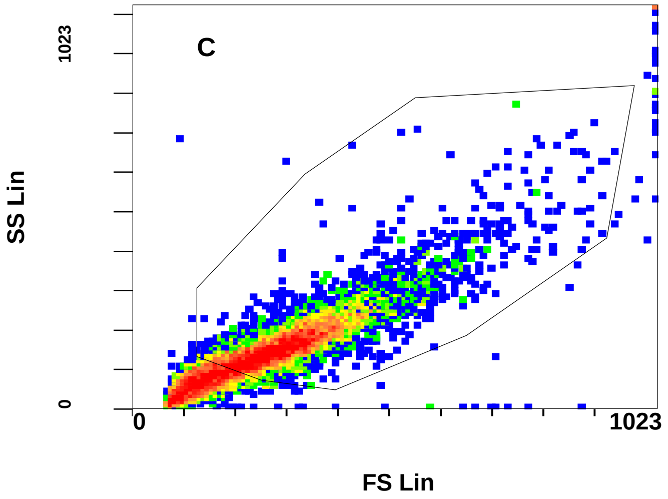

(F1)[Ungated] 27_00010675 538.LMD : FL3 Lin/AUX - ADC

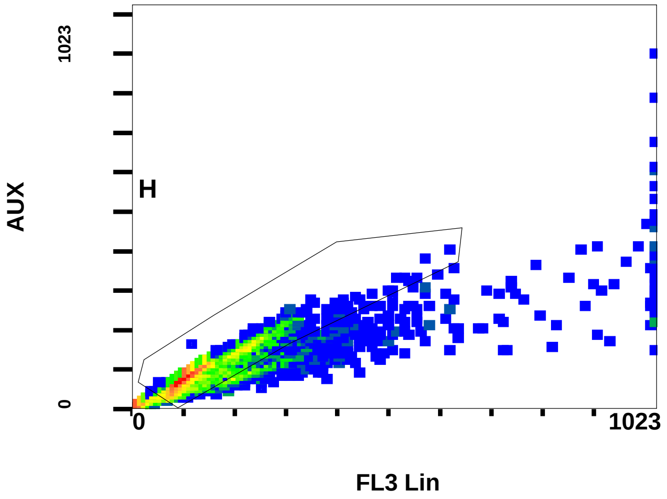

(F1)[H] 27_00010675 538.LMD : FL3 Lin - ADC

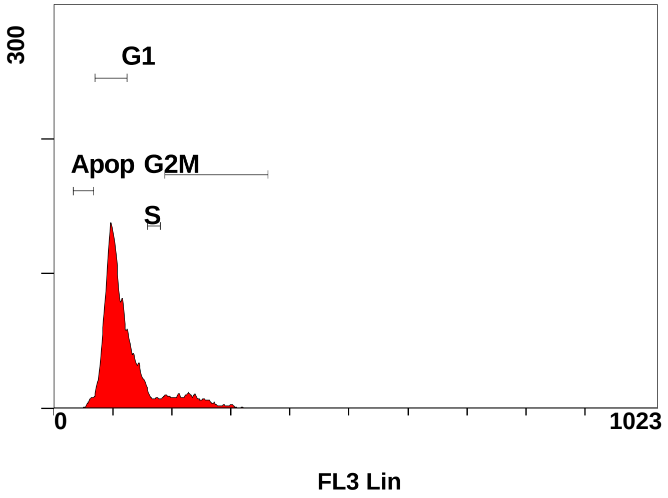

(F1)[H] 27_00010675 538.LMD : FL3 Lin/SS Lin - ADC

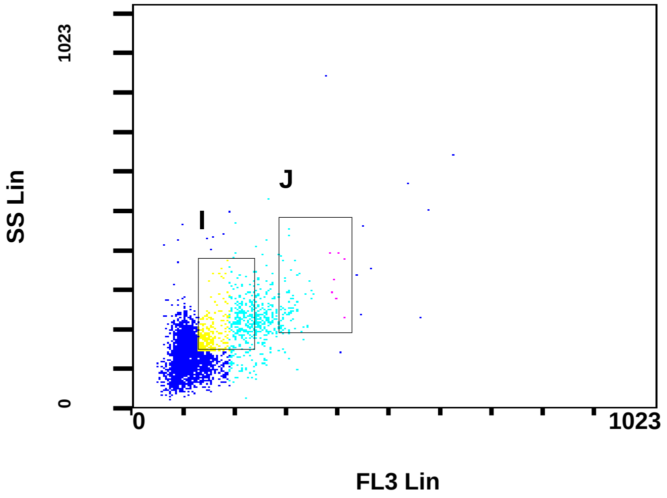

Statistical Analysis

PROGRAM INFORMATION

File:- 27_00010675 538.LMD
Gate:- H [H]
Compensation:- Advanced
Filename:- 27_00010675 538.LMD
Mean Calculation Method:-LOG-LOG

Region	Number	%Total	%Gated	X-Mean	X-HPCV	Y-Mean
ALL	7188	71.88	100.00	129	11.11	###
ALL	7188	71.88	100.00	129	11.11	155
Apop	124	1.24	1.73	60.2	2.02	###
G1	4530	45.30	63.02	100	11.11	###
G2M	1010	10.10	14.05	240	0.55	###
I	1072	10.72	14.91	178	1.44	206
J	137	1.37	1.91	313	0.86	266
S	225	2.25	3.13	168	1.33	###

File:- 27_00010675 538.LMD
Gate:- Ungated
Compensation:- Advanced
Filename:- 27_00010675 538.LMD
Mean Calculation Method:-LOG-LOG

Region	Number	%Total	%Gated	X-Mean	X-HPCV	Y-Mean
ALL	10000	100.00	100.00	266	6.65	146
ALL	10000	100.00	100.00	117	###	76.2
C	6937	69.37	69.37	318	6.44	177
H	7188	71.88	71.88	129	11.11	91.8

Institution:

Run Date: 03-May-12, 11:46:25

Protocol: 202_00010650 513.PRO

Sample ID: Well 27

Listmode Replay: New Protocol

User ID: anajavi

Analysis Date: 03-May-2012, 13:23:37

Acquisition Time/Events: 117.9s / 10000 (UNKNOWN)

Settings File: 00Ciclo Celular de Javi.PRO, 03-May-2012, 10:53:20

Tube ID: NoRead

Listmode File: 27_00010675 538.LMD

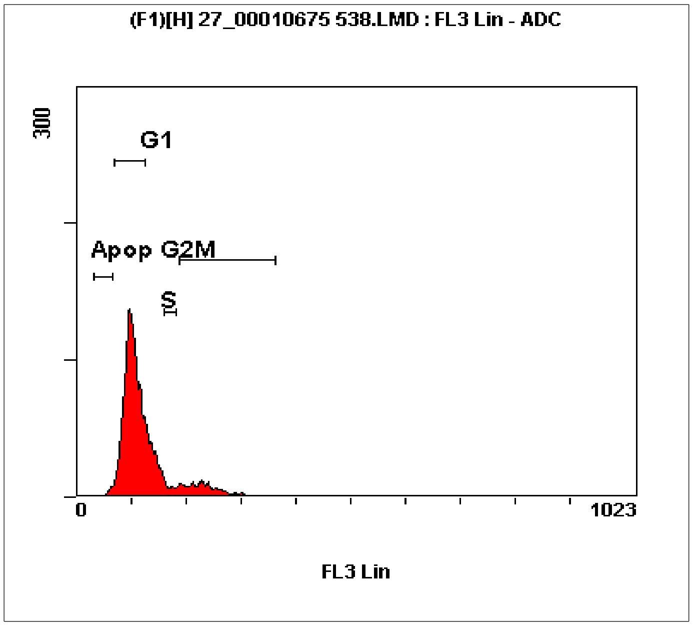

(F1)[H] 27_00010675 538.LMD : FL3 Lin

Region	Number	%Total	%Gated	X-Mean	HP X-CV	Y-Mea
ALL	7188	71.88	100.00	129	11.11	###
Apop	124	1.24	1.73	60.2	2.02	###
G1	4530	45.30	63.02	100	11.11	###
G2M	1010	10.10	14.05	240	0.55	###
S	225	2.25	3.13	168	1.33	###

Institution:
Protocol: 202_00010650 513.PRO
Listmode Replay: New Protocol
Analysis Date: 03-May-2012, 13:08:17
Settings File: 00Ciclo Celular de Javi.PRO, 03-May-2012, 10:53:20
Listmode File: 03_00010678 541.LMD

Run Date: 03-May-12, 11:55:16
Sample ID: Well 03
User ID: anajavi
Acquisition Time/Events: 76.9s / 10000 (UNKNOWN)

Instrument SN: AL01002 Software Version: CXP v2.2

(F1)[Ungated] 03_00010678 541.LMD : FS Lin/SS Lin - ADC

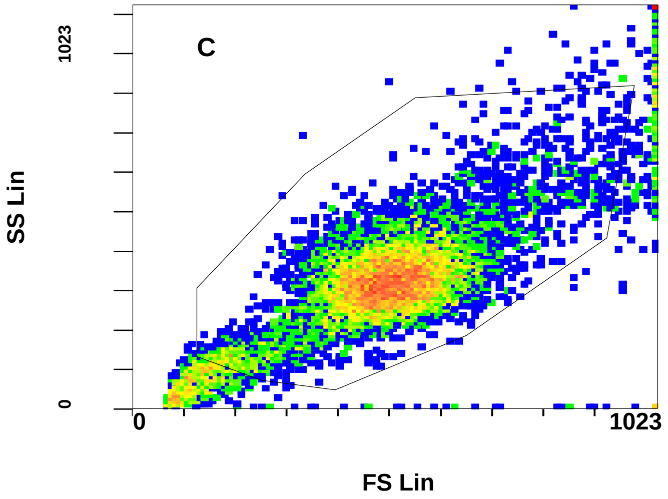

(F1)[Ungated] 03_00010678 541.LMD : FL3 Lin/AUX - ADC

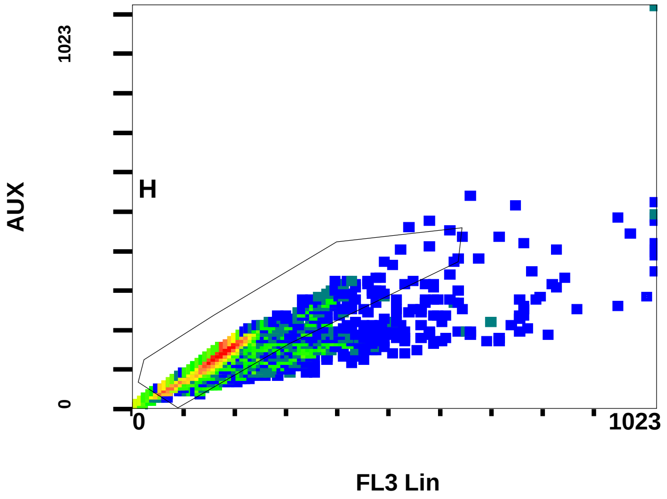

(F1)[H] 03_00010678 541.LMD : FL3 Lin - ADC

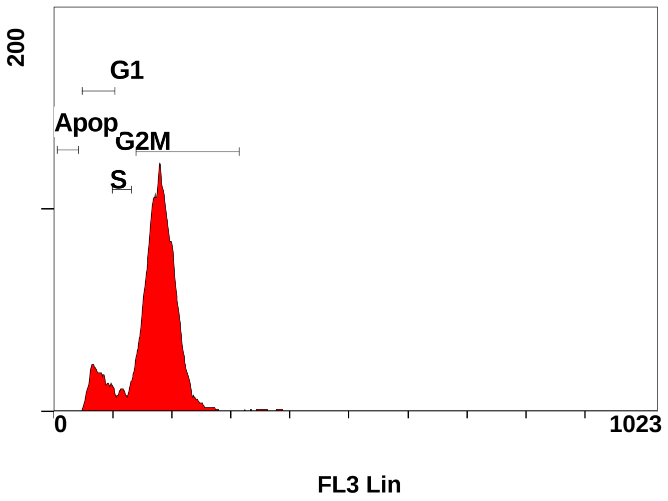

(F1)[H] 03_00010678 541.LMD : FL3 Lin/SS Lin - ADC

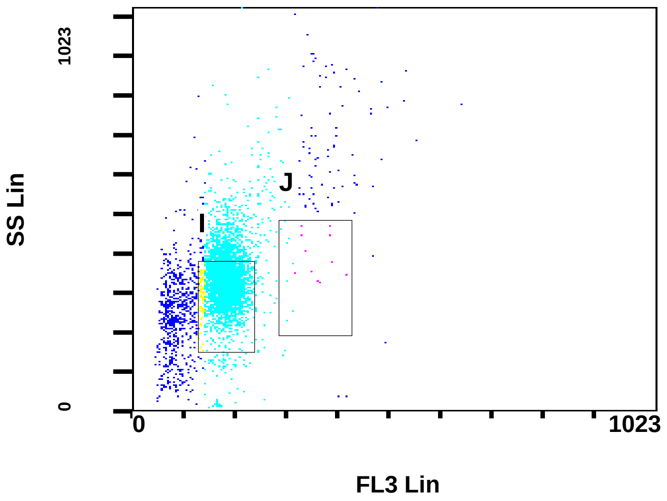

Statistical Analysis

PROGRAM INFORMATION

File:- 03_00010678 541.LMD
Gate:- H [H]
Compensation:- Advanced
Filename:- 03_00010678 541.LMD
Mean Calculation Method:-LOG-LOG

Region	Number	%Total	%Gated	X-Mean	X-HPCV	Y-Mean
ALL	8465	84.65	100.00	173	11.51	###
ALL	8465	84.65	100.00	173	11.51	334
Apop	0	0.00	0.00	0	0.00	###
G1	950	9.50	11.22	76.5	9.93	###
G2M	6872	68.72	81.18	185	11.51	###
I	5054	50.54	59.70	180	11.12	311
J	30	0.30	0.35	340	0.14	398
S	388	3.88	4.58	115	2.01	###

File:- 03_00010678 541.LMD
Gate:- Ungated
Compensation:- Advanced
Filename:- 03_00010678 541.LMD
Mean Calculation Method:-LOG-LOG

Region	Number	%Total	%Gated	X-Mean	X-HPCV	Y-Mean
ALL	10000	100.00	100.00	167	99.18	123
ALL	10000	100.00	100.00	491	0.00	325
C	8787	87.87	87.87	503	3.10	332
H	8465	84.65	84.65	173	11.51	135

Institution:

Run Date: 03-May-12, 11:55:16

Protocol: 202_00010650 513.PRO

Sample ID: Well 03

Listmode Replay: New Protocol

User ID: anajavi

Analysis Date: 03-May-2012, 13:08:17

Acquisition Time/Events: 76.9s / 10000 (UNKNOWN)

Settings File: 00Ciclo Celular de Javi.PRO, 03-May-2012, 10:53:20

Tube ID: NoRead

Listmode File: 03_00010678 541.LMD

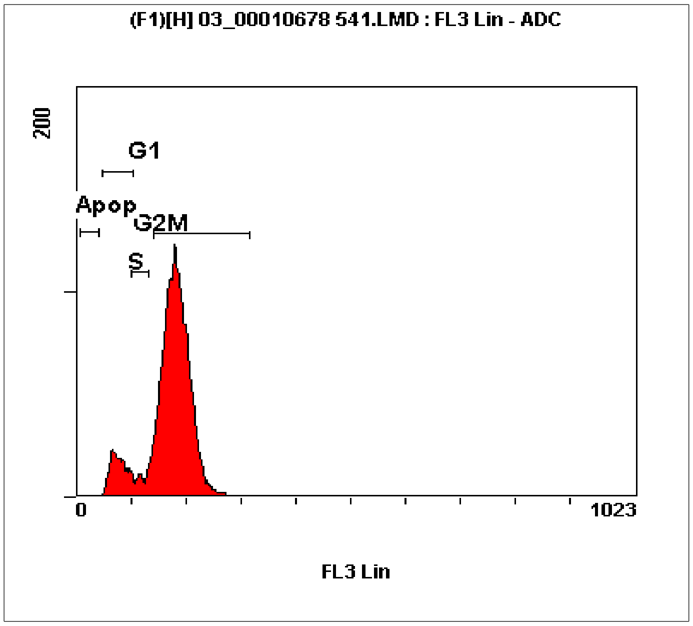

(F1)[H] 03_00010678 541.LMD : FL3 Lin						
Region	Number	%Total	%Gated	X-Mean	HP X-CV	Y-Mea
ALL	8465	84.65	100.00	173	11.51	###
Apop	0	0.00	0.00	0	0.00	###
G1	950	9.50	11.22	76.5	9.93	###
G2M	6872	68.72	81.18	185	11.51	###
S	388	3.88	4.58	115	2.01	###

Institution:
Protocol: 202_00010650 513.PRO
Listmode Replay: New Protocol
Analysis Date: 03-May-2012, 13:08:53
Settings File: 00Ciclo Celular de Javi.PRO, 03-May-2012, 10:53:20
Listmode File: 04_00010679 542.LMD

Run Date: 03-May-12, 11:57:09
Sample ID: Well 04
User ID: anajavi
Acquisition Time/Events: 119.1s / 10000 (UNKNOWN)

Instrument SN: AL01002 Software Version: CXP v2.2

(F1)[Ungated] 04_00010679 542.LMD : FS Lin/SS Lin - ADC

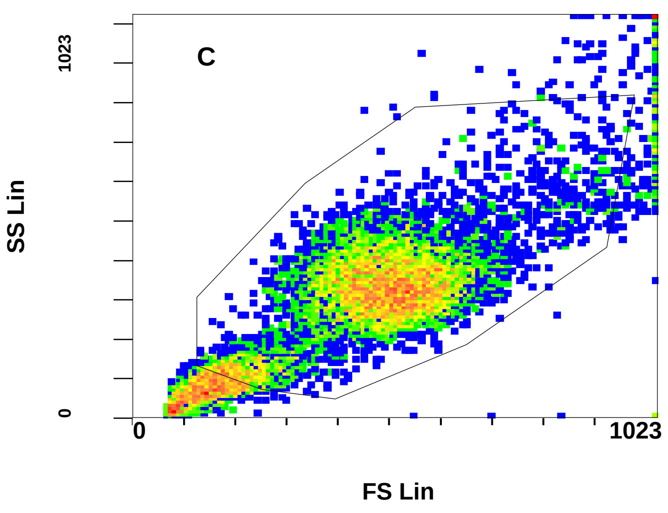

(F1)[Ungated] 04_00010679 542.LMD : FL3 Lin/AUX - ADC

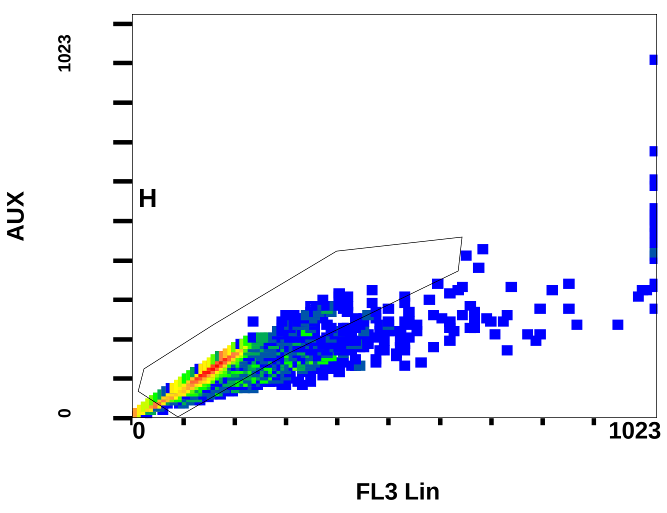

(F1)[H] 04_00010679 542.LMD : FL3 Lin - ADC

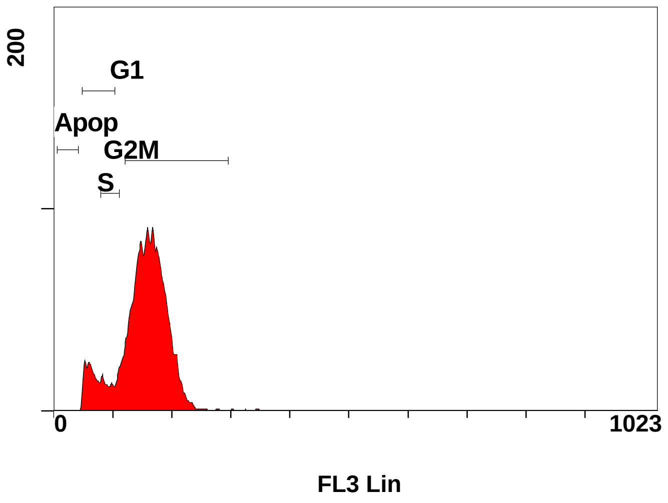

(F1)[H] 04_00010679 542.LMD : FL3 Lin/SS Lin - ADC

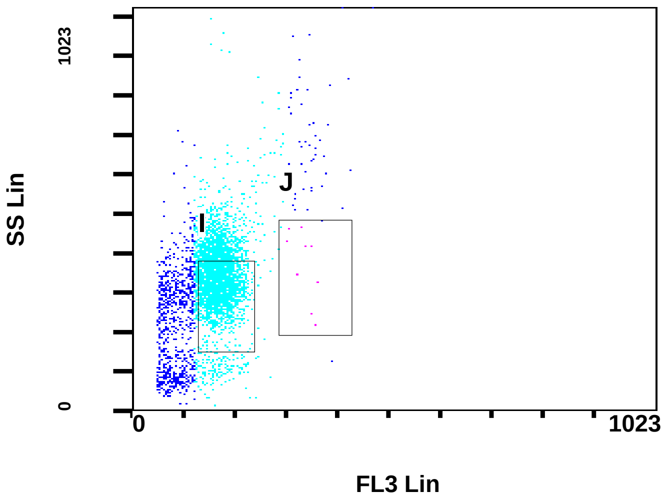

Statistical Analysis

PROGRAM INFORMATION

File:- 04_00010679 542.LMD

Gate:- H [H]

Compensation:- Advanced

Filename:- 04_00010679 542.LMD

Mean Calculation Method:-LOG-LOG

Region	Number	%Total	%Gated	X-Mean	X-HPCV	Y-Mean
ALL	7759	77.59	100.00	154	14.12	###
ALL	7759	77.59	100.00	154	14.12	328
Apop	0	0.00	0.00	0	0.00	###
G1	1038	10.38	13.38	72.6	1.18	###
G2M	6228	62.28	80.27	167	14.12	###
I	3705	37.05	47.75	168	9.36	312
J	27	0.27	0.35	326	0.14	377
S	547	5.47	7.05	95.3	1.67	###

File:- 04_00010679 542.LMD

Gate:- Ungated

Compensation:- Advanced

Filename:- 04_00010679 542.LMD

Mean Calculation Method:-LOG-LOG

Region	Number	%Total	%Gated	X-Mean	X-HPCV	Y-Mean
ALL	10000	100.00	100.00	138	76.40	102
ALL	10000	100.00	100.00	444	0.00	302
C	8159	81.59	81.59	480	2.68	327
H	7759	77.59	77.59	154	14.12	121

Institution:

Protocol: 202_00010650 513.PRO

Listmode Replay: New Protocol

Analysis Date: 03-May-2012, 13:08:53

Settings File: 00Ciclo Celular de Javi.PRO, 03-May-2012, 10:53:20

Listmode File: 04_00010679 542.LMD

Run Date: 03-May-12, 11:57:09

Sample ID: Well 04

User ID: anajavi

Acquisition Time/Events: 119.1s / 10000 (UNKNOWN)

Tube ID: NoRead

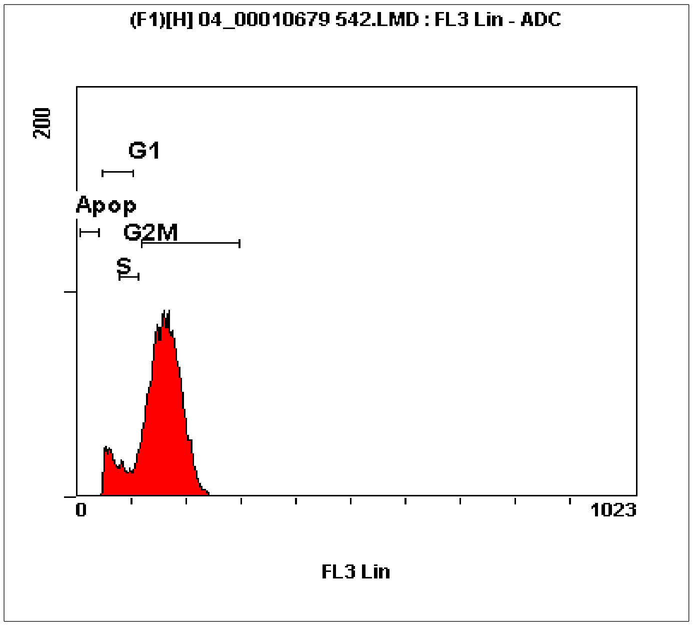

(F1)[H] 04_00010679 542.LMD : FL3 Lin

Region	Number	%Total	%Gated	X-Mean	HP X-CV	Y-Mea
ALL	7759	77.59	100.00	154	14.12	###
Apop	0	0.00	0.00	0	0.00	###
G1	1038	10.38	13.38	72.6	1.18	###
G2M	6228	62.28	80.27	167	14.12	###
S	547	5.47	7.05	95.3	1.67	###

Institution:
Protocol: 01_00010606 476.PRO
Listmode Replay: New Protocol
Analysis Date: 03-May-2012, 12:41:22
Settings File: 00Ciclo Celular de Javi.PRO, 18-Apr-2012, 11:34:20
Listmode File: 101_00010649 512.LMD

Run Date: 02-May-12, 14:50:47
Sample ID: Well 01
User ID: anajavi
Acquisition Time/Events: 36.7s / 10000 (UNKNOWN)

Instrument SN: AL01002 Software Version: CXP v2.2

(F1)[Ungated] 101_00010649 512.LMD : FS Lin/SS Lin - ADC

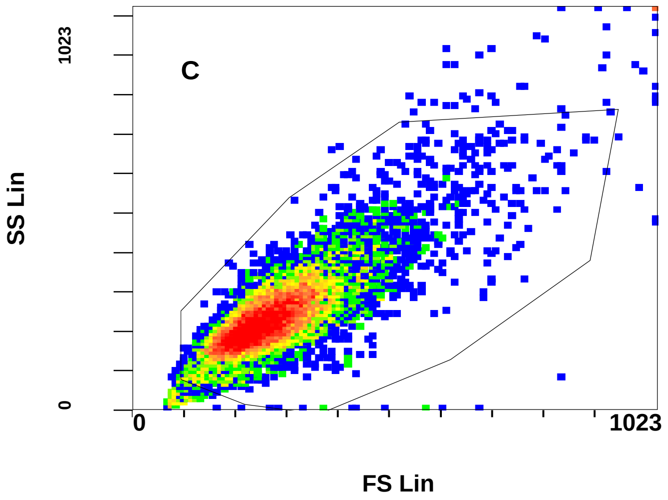

(F1)[Ungated] 101_00010649 512.LMD : FL3 Lin/AUX - ADC

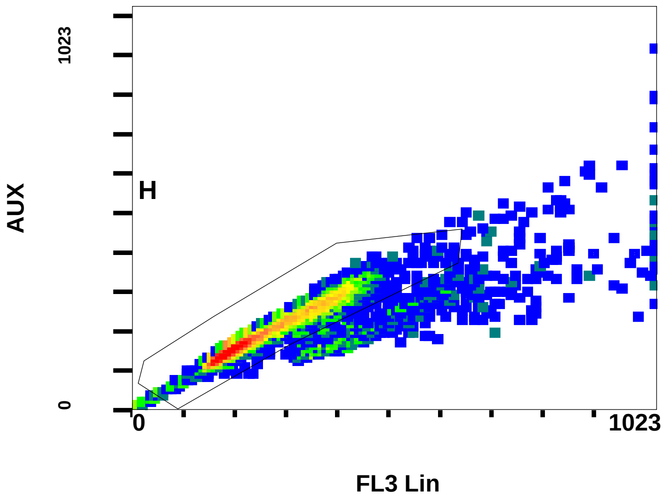

(F1)[H] 101_00010649 512.LMD : FL3 Lin - ADC

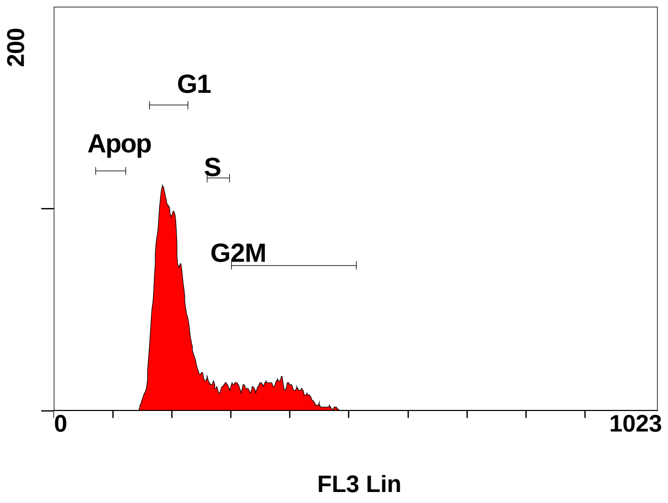

(F1)[H] 101_00010649 512.LMD : FL3 Lin/SS Lin - ADC

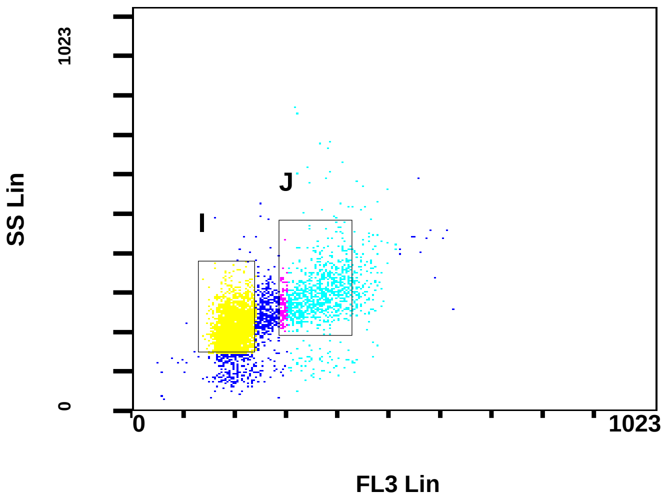

Statistical Analysis

PROGRAM INFORMATION

File:- 101_00010649 512.LMD						
Gate:- H [H]						
Compensation:- Advanced						
Filename:- 101_00010649 512.LMD						
Mean Calculation Method:-LOG-LOG						
Region	Number	%Total	%Gated	X-Mean	X-HPCV	Y-Mean
ALL	9260	92.60	100.00	244	11.05	###
ALL	9260	92.60	100.00	244	11.05	232
Apop	27	0.27	0.29	99.3	0.85	###
G1	5504	55.04	59.44	194	11.05	###
G2M	2066	20.66	22.31	376	1.66	###
I	5610	56.10	60.58	195	9.89	212
J	1844	18.44	19.91	356	1.96	298
S	555	5.55	5.99	277	0.73	###

File:- 101_00010649 512.LMD						
Gate:- Ungated						
Compensation:- Advanced						
Filename:- 101_00010649 512.LMD						
Mean Calculation Method:-LOG-LOG						
Region	Number	%Total	%Gated	X-Mean	X-HPCV	Y-Mean
ALL	10000	100.00	100.00	258	11.05	187
ALL	10000	100.00	100.00	285	18.05	243
C	9792	97.92	97.92	284	18.05	243
H	9260	92.60	92.60	244	11.05	186

Institution:

Protocol: 01_00010606 476.PRO

Listmode Replay: New Protocol

Analysis Date: 03-May-2012, 12:41:23

Settings File: 00Ciclo Celular de Javi.PRO, 18-Apr-2012, 11:34:20

Listmode File: 101_00010649 512.LMD

Run Date: 02-May-12, 14:50:47

Sample ID: Well 01

User ID: anajavi

Acquisition Time/Events: 36.7s / 10000 (UNKNOWN)

Tube ID: NoRead

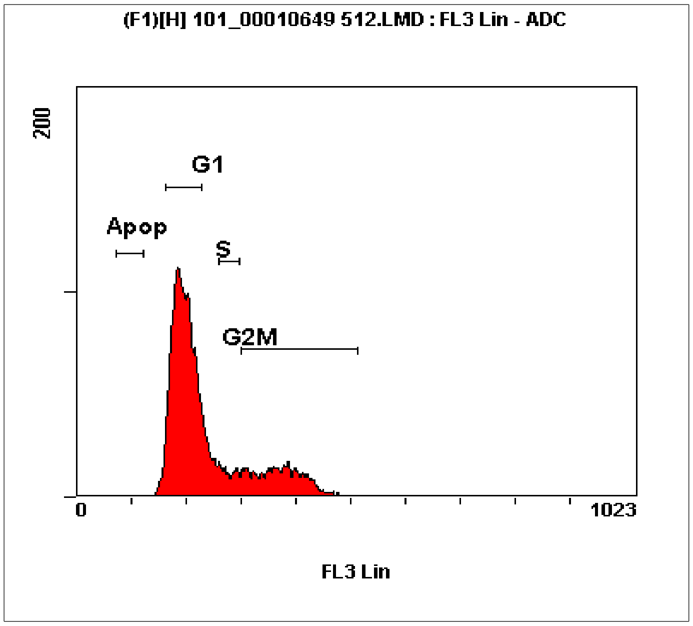

(F1)[H] 101_00010649 512.LMD : FL3 Lin						
Region	Number	%Total	%Gated	X-Mean	HP X-CV	Y-Mea
ALL	9260	92.60	100.00	244	11.05	###
Apop	27	0.27	0.29	99.3	0.85	###
G1	5504	55.04	59.44	194	11.05	###
G2M	2066	20.66	22.31	376	1.66	###
S	555	5.55	5.99	277	0.73	###

Institution:
Protocol: 202_00010650 513.PRO
Listmode Replay: New Protocol
Analysis Date: 03-May-2012, 12:52:21
Settings File: 00Ciclo Celular de Javi.PRO, 18-Apr-2012, 11:34:20
Listmode File: 202_00010650 513.LMD

Run Date: 02-May-12, 14:51:59
Sample ID: Well 02
User ID: anajavi
Acquisition Time/Events: 49.6s / 10000 (UNKNOWN)

Instrument SN: AL01002 Software Version: CXP v2.2

(F1)[Ungated] 202_00010650 513.LMD : FS Lin/SS Lin - ADC

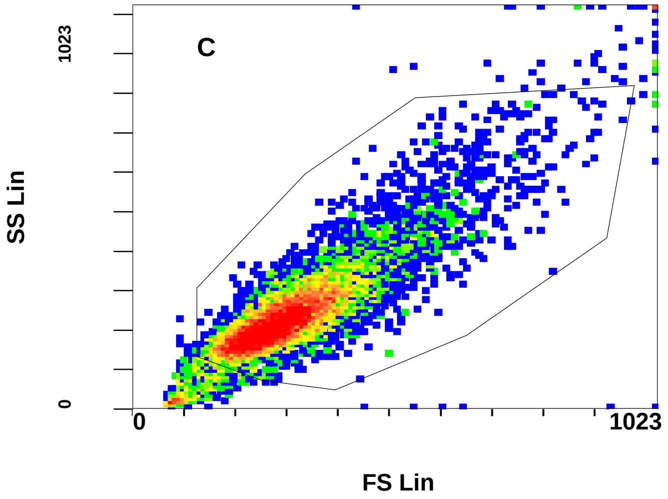

(F1)[Ungated] 202_00010650 513.LMD : FL3 Lin/AUX - ADC

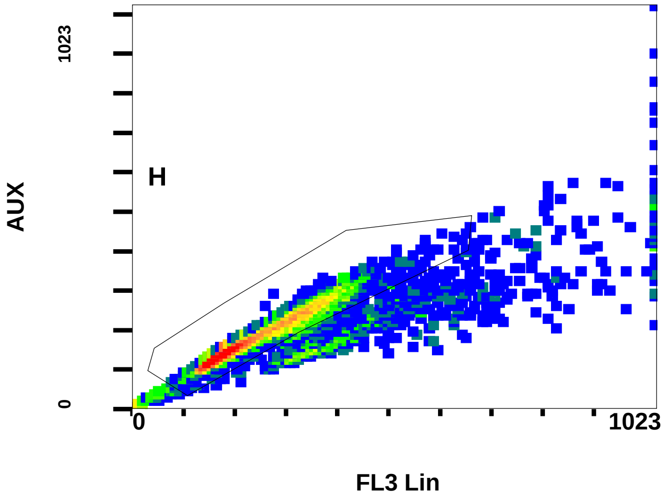

(F1)[H] 202_00010650 513.LMD : FL3 Lin - ADC

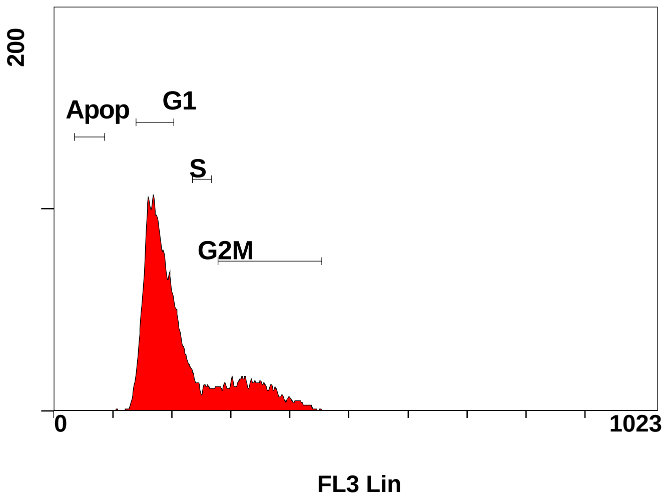

(F1)[H] 202_00010650 513.LMD : FL3 Lin/SS Lin - ADC

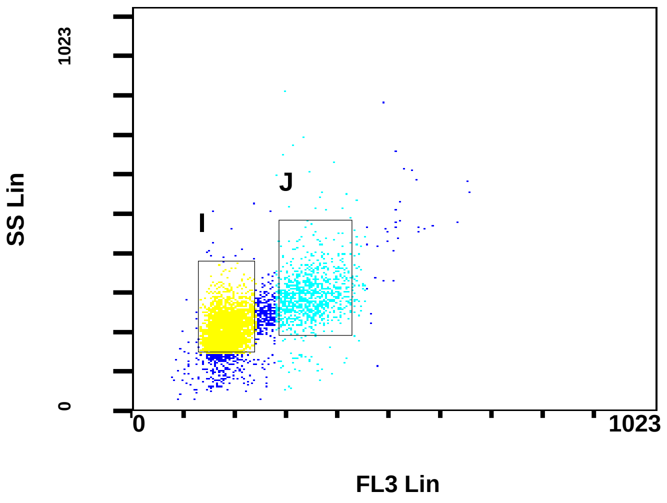

Statistical Analysis

PROGRAM INFORMATION

File:- 202_00010650 513.LMD						
Gate:- H [H]						
Compensation:- Advanced						
Filename:- 202_00010650 513.LMD						
Mean Calculation Method:-LOG-LOG						
Region	Number	%Total	%Gated	X-Mean	X-HPCV	Y-Mean
ALL	8868	88.68	100.00	223	10.72	###
ALL	8868	88.68	100.00	223	10.72	226
Apop	6	0.06	0.07	81.8	1.13	###
G1	4980	49.80	56.16	172	10.72	###
G2M	1941	19.41	21.89	345	0.37	###
I	5703	57.03	64.31	181	10.62	209
J	1669	16.69	18.82	345	0.37	297
S	482	4.82	5.44	250	1.09	###

File:- 202_00010650 513.LMD						
Gate:- Ungated						
Compensation:- Advanced						
Filename:- 202_00010650 513.LMD						
Mean Calculation Method:-LOG-LOG						
Region	Number	%Total	%Gated	X-Mean	X-HPCV	Y-Mean
ALL	10000	100.00	100.00	241	10.72	171
ALL	10000	100.00	100.00	311	12.64	241
C	9408	94.08	94.08	317	12.63	247
H	8868	88.68	88.68	223	10.72	170

Institution:

Run Date: 02-May-12, 14:51:59

Protocol: 202_00010650 513.PRO

Sample ID: Well 02

Listmode Replay: New Protocol

User ID: anajavi

Analysis Date: 03-May-2012, 12:52:21

Acquisition Time/Events: 49.6s / 10000 (UNKNOWN)

Settings File: 00Ciclo Celular de Javi.PRO, 18-Apr-2012, 11:34:20

Tube ID: NoRead

Listmode File: 202_00010650 513.LMD

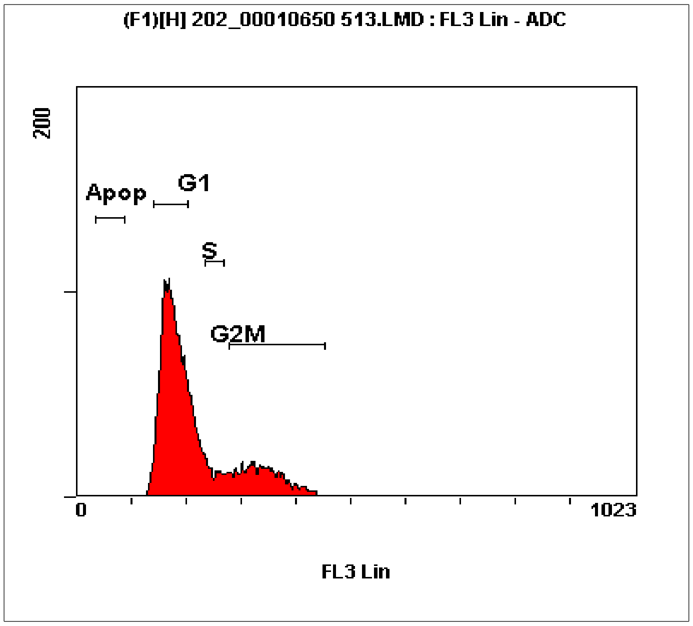

(F1)[H] 202_00010650 513.LMD : FL3 Lin

Region	Number	%Total	%Gated	X-Mean	HP X-CV	Y-Mea
ALL	8868	88.68	100.00	223	10.72	###
Apop	6	0.06	0.07	81.8	1.13	###
G1	4980	49.80	56.16	172	10.72	###
G2M	1941	19.41	21.89	345	0.37	###
S	482	4.82	5.44	250	1.09	###

202_00010650 513.PRO : CXP

Institution:
Protocol: 202_00010650 513.PRO
Listmode Replay: New Protocol
Analysis Date: 03-May-2012, 12:59:02
Settings File: 00Ciclo Celular de Javi.PRO, 18-Apr-2012, 11:34:20
Listmode File: 28_00010640 503.LMD

Run Date: 02-May-12, 14:21:21
Sample ID: Well 28
User ID: anajavi
Acquisition Time/Events: 184.4s / 10000 (UNKNOWN)

Instrument SN: AL01002 Software Version: CXP v2.2

(F1)[Ungated] 28_00010640 503.LMD : FS Lin/SS Lin - ADC

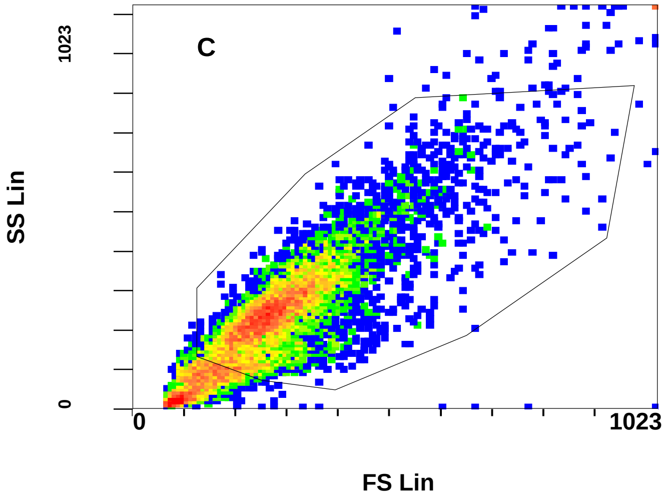

(F1)[Ungated] 28_00010640 503.LMD : FL3 Lin/AUX - ADC

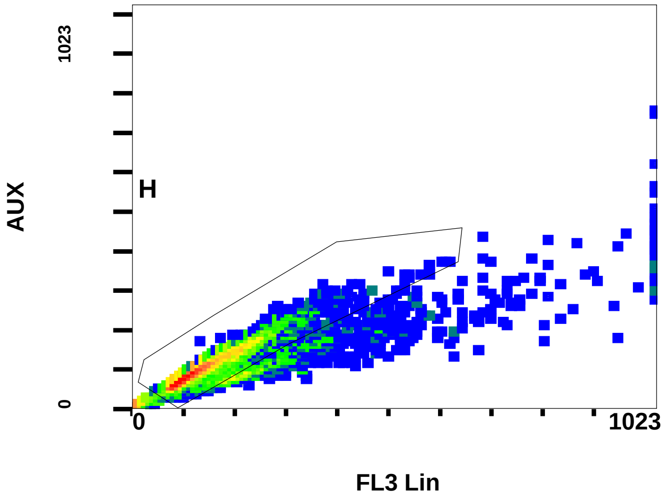

(F1)[H] 28_00010640 503.LMD : FL3 Lin - ADC

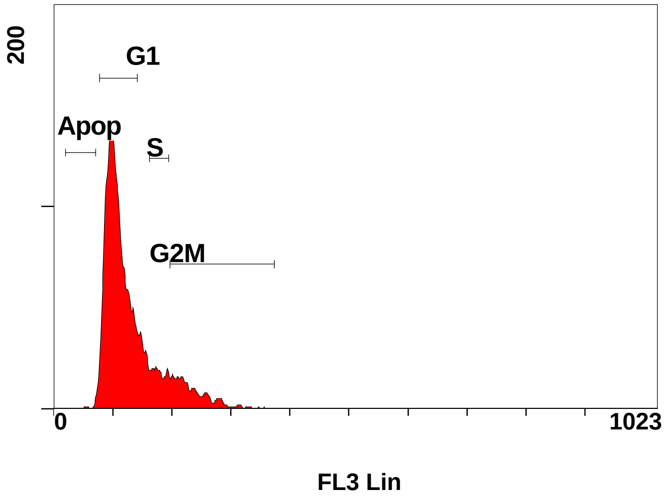

(F1)[H] 28_00010640 503.LMD : FL3 Lin/SS Lin - ADC

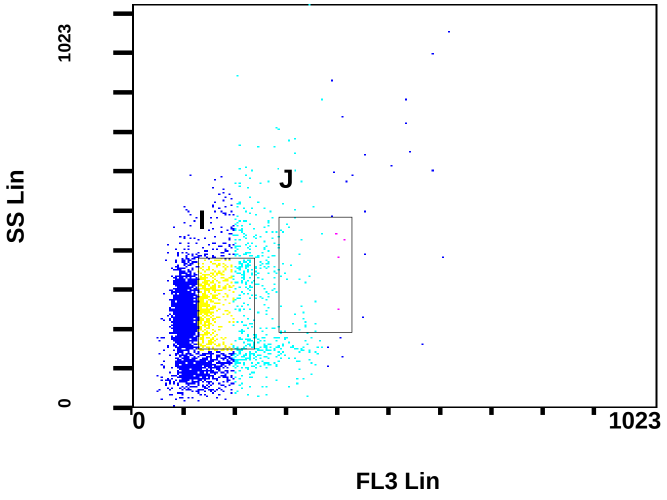

Statistical Analysis

PROGRAM INFORMATION

File:- 28_00010640 503.LMD						
Gate:- H [H]						
Compensation:- Advanced						
Filename:- 28_00010640 503.LMD						
Mean Calculation Method:-LOG-LOG						
Region	Number	%Total	%Gated	X-Mean	X-HPCV	Y-Mean
ALL	8144	81.44	100.00	137	12.30	###
ALL	8144	81.44	100.00	137	12.30	228
Apop	60	0.60	0.74	61	0.81	###
G1	5458	54.58	67.02	106	12.30	###
G2M	1173	11.73	14.40	245	1.61	###
I	1358	13.58	16.67	166	4.07	262
J	82	0.82	1.01	329	0.69	316
S	664	6.64	8.15	178	0.53	###

File:- 28_00010640 503.LMD						
Gate:- Ungated						
Compensation:- Advanced						
Filename:- 28_00010640 503.LMD						
Mean Calculation Method:-LOG-LOG						
Region	Number	%Total	%Gated	X-Mean	X-HPCV	Y-Mean
ALL	10000	100.00	100.00	139	###	94.1
ALL	10000	100.00	100.00	270	7.90	221
C	7651	76.51	76.51	308	7.90	263
H	8144	81.44	81.44	137	12.30	101

Institution:

Protocol: 202_00010650 513.PRO

Listmode Replay: New Protocol

Analysis Date: 03-May-2012, 12:59:02

Settings File: 00Ciclo Celular de Javi.PRO, 18-Apr-2012, 11:34:20

Listmode File: 28_00010640 503.LMD

Run Date: 02-May-12, 14:21:21

Sample ID: Well 28

User ID: anajavi

Acquisition Time/Events: 184.4s / 10000 (UNKNOWN)

Tube ID: NoRead

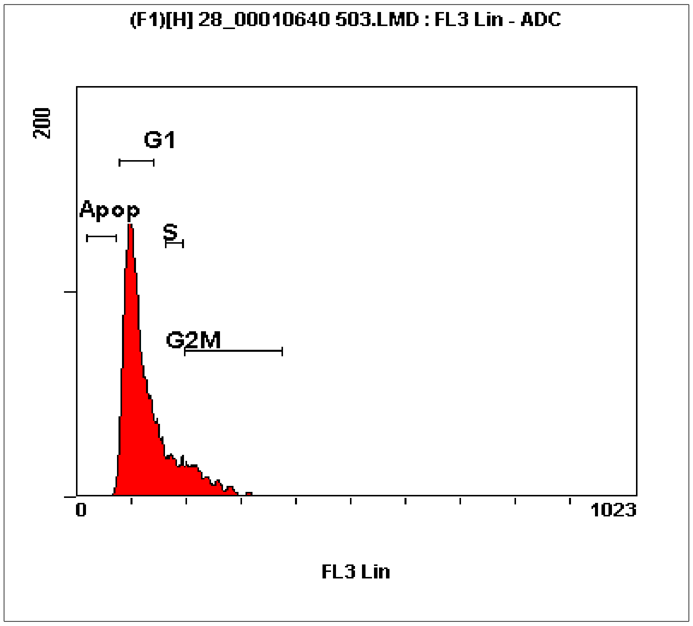

(F1)[H] 28_00010640 503.LMD : FL3 Lin

Region	Number	%Total	%Gated	X-Mean	HP X-CV	Y-Mea
ALL	8144	81.44	100.00	137	12.30	###
Apop	60	0.60	0.74	61	0.81	###
G1	5458	54.58	67.02	106	12.30	###
G2M	1173	11.73	14.40	245	1.61	###
S	664	6.64	8.15	178	0.53	###

Institution:
Protocol: 202_00010650 513.PRO
Listmode Replay: New Protocol
Analysis Date: 03-May-2012, 13:00:00
Settings File: 00Ciclo Celular de Javi.PRO, 18-Apr-2012, 11:34:20
Listmode File: 29_00010641 504.LMD

Run Date: 02-May-12, 14:25:01
Sample ID: Well 29
User ID: anajavi
Acquisition Time/Events: 65.2s / 10000 (UNKNOWN)

Instrument SN: AL01002 Software Version: CXP v2.2

(F1)[Ungated] 29_00010641 504.LMD : FS Lin/SS Lin - ADC

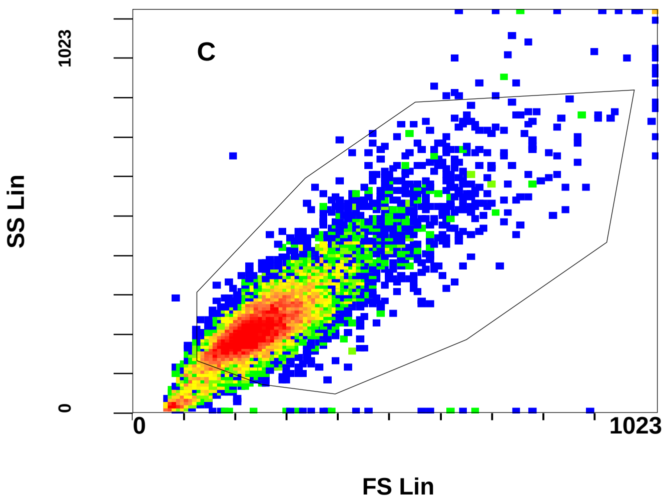

(F1)[Ungated] 29_00010641 504.LMD : FL3 Lin/AUX - ADC

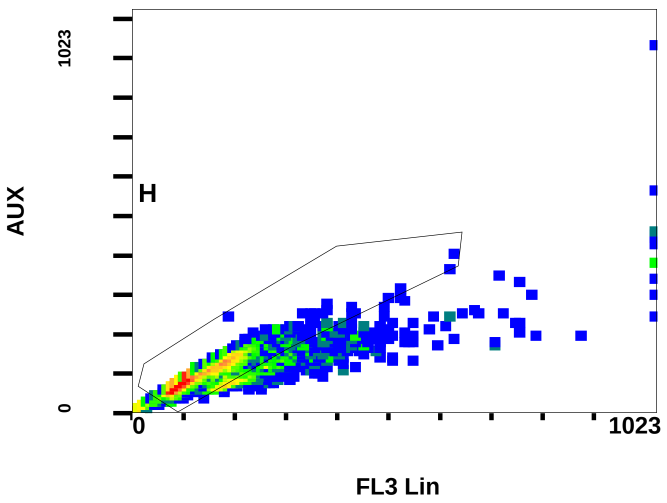

(F1)[H] 29_00010641 504.LMD : FL3 Lin - ADC

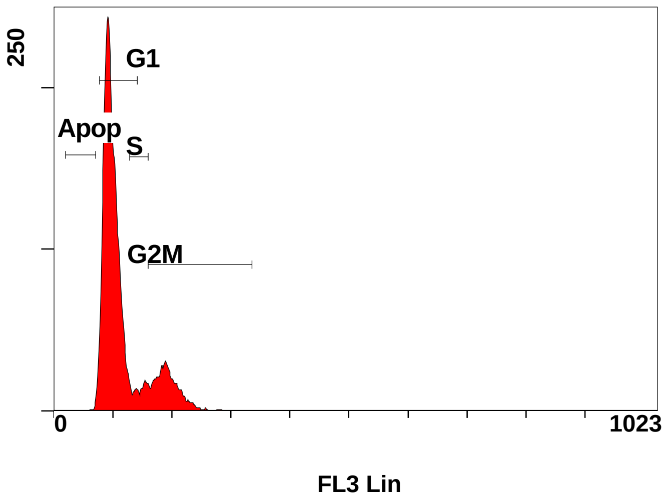

(F1)[H] 29_00010641 504.LMD : FL3 Lin/SS Lin - ADC

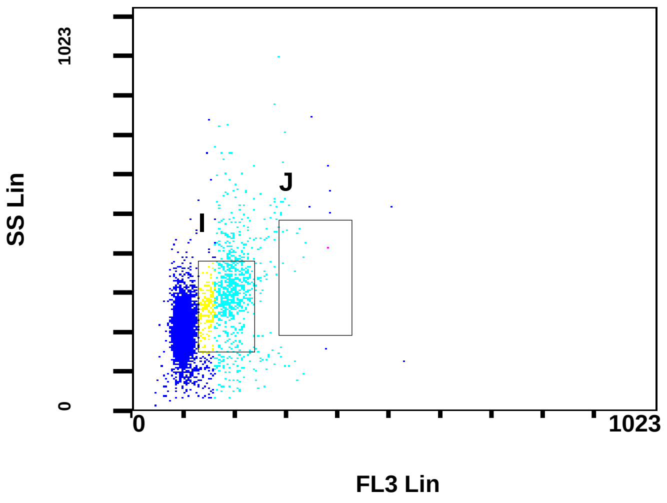

Statistical Analysis

PROGRAM INFORMATION

File:- 29_00010641 504.LMD						
Gate:- H [H]						
Compensation:- Advanced						
Filename:- 29_00010641 504.LMD						
Mean Calculation Method:-LOG-LOG						
Region	Number	%Total	%Gated	X-Mean	X-HPCV	Y-Mean
ALL	8783	87.83	100.00	119	10.29	###
ALL	8783	87.83	100.00	119	10.29	228
Apop	60	0.60	0.68	63	1.20	###
G1	6660	66.60	75.83	98	10.29	###
G2M	1626	16.26	18.51	200	4.88	###
I	1422	14.22	16.19	179	3.40	283
J	24	0.24	0.27	326	0.22	393
S	523	5.23	5.95	145	2.64	###

File:- 29_00010641 504.LMD						
Gate:- Ungated						
Compensation:- Advanced						
Filename:- 29_00010641 504.LMD						
Mean Calculation Method:-LOG-LOG						
Region	Number	%Total	%Gated	X-Mean	X-HPCV	Y-Mean
ALL	10000	100.00	100.00	269	9.12	232
ALL	10000	100.00	100.00	124	10.29	85.1
C	8951	89.51	89.51	282	9.11	248
H	8783	87.83	87.83	119	10.29	87.3

Institution:

Protocol: 202_00010650 513.PRO

Listmode Replay: New Protocol

Analysis Date: 03-May-2012, 13:00:00

Settings File: 00Ciclo Celular de Javi.PRO, 18-Apr-2012, 11:34:20

Listmode File: 29_00010641 504.LMD

Run Date: 02-May-12, 14:25:01

Sample ID: Well 29

User ID: anajavi

Acquisition Time/Events: 65.2s / 10000 (UNKNOWN)

Tube ID: NoRead

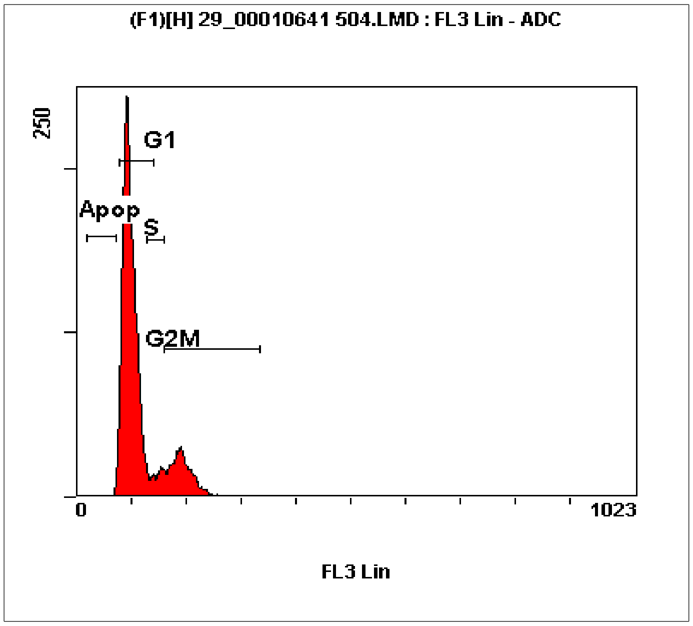

(F1)[H] 29_00010641 504.LMD : FL3 Lin

Region	Number	%Total	%Gated	X-Mean	HP X-CV	Y-Mea
ALL	8783	87.83	100.00	119	10.29	###
Apop	60	0.60	0.68	63	1.20	###
G1	6660	66.60	75.83	98	10.29	###
G2M	1626	16.26	18.51	200	4.88	###
S	523	5.23	5.95	145	2.64	###

Institution:
Protocol: 202_00010650 513.PRO
Listmode Replay: New Protocol
Analysis Date: 03-May-2012, 13:00:43
Settings File: 00Ciclo Celular de Javi.PRO, 18-Apr-2012, 11:34:20
Listmode File: 30_00010642 505.LMD

Run Date: 02-May-12, 14:26:43
Sample ID: Well 30
User ID: anajavi
Acquisition Time/Events: 132.0s / 10000 (UNKNOWN)

Instrument SN: AL01002 Software Version: CXP v2.2

(F1)[Ungated] 30_00010642 505.LMD : FS Lin/SS Lin - ADC

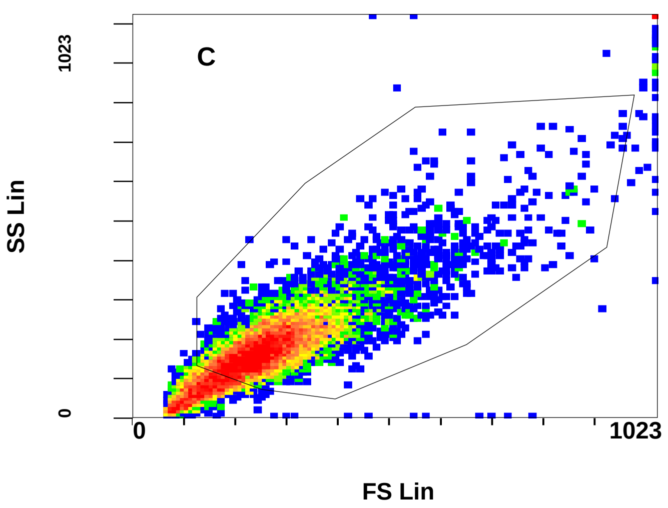

(F1)[Ungated] 30_00010642 505.LMD : FL3 Lin/AUX - ADC

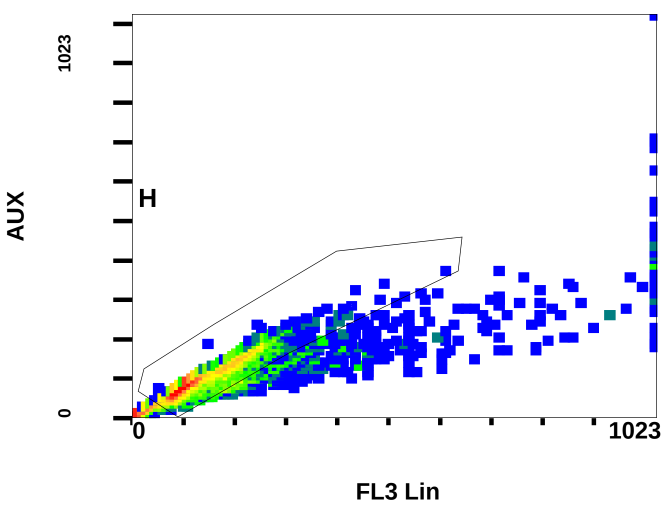

(F1)[H] 30_00010642 505.LMD : FL3 Lin - ADC

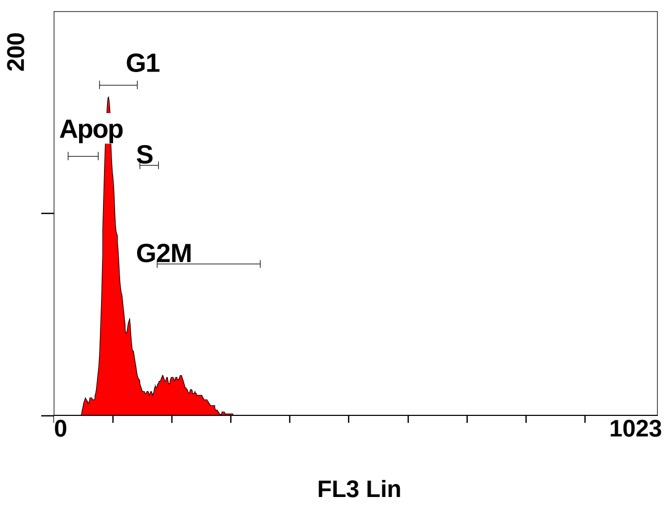

(F1)[H] 30_00010642 505.LMD : FL3 Lin/SS Lin - ADC

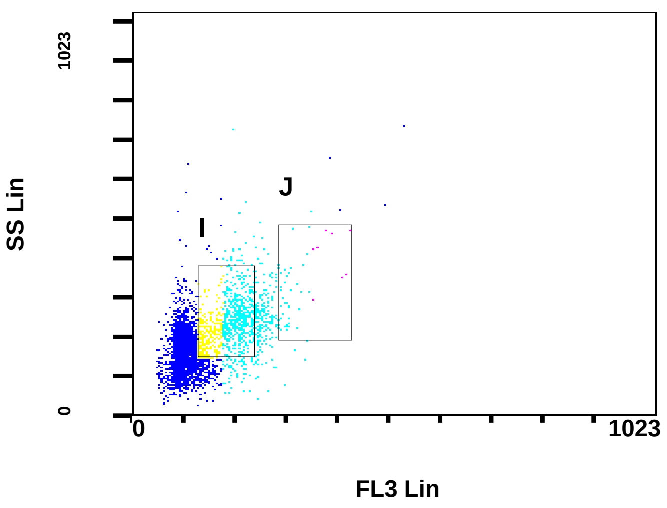

Statistical Analysis

PROGRAM INFORMATION

File:- 30_00010642 505.LMD
Gate:- H [H]
Compensation:- Advanced
Filename:- 30_00010642 505.LMD
Mean Calculation Method:-LOG-LOG

Region	Number	%Total	%Gated	X-Mean	X-HPCV	Y-Mean
ALL	7573	75.73	100.00	131	9.43	###
ALL	7573	75.73	100.00	131	9.43	186
Apop	300	3.00	3.96	63.5	2.67	###
G1	5113	51.13	67.52	102	9.43	###
G2M	1620	16.20	21.39	222	0.66	###
I	1594	15.94	21.05	183	1.14	236
J	79	0.79	1.04	319	0.27	324
S	478	4.78	6.31	161	0.65	###

File:- 30_00010642 505.LMD
Gate:- Ungated
Compensation:- Advanced
Filename:- 30_00010642 505.LMD
Mean Calculation Method:-LOG-LOG

Region	Number	%Total	%Gated	X-Mean	X-HPCV	Y-Mean
ALL	10000	100.00	100.00	124	###	82
ALL	10000	100.00	100.00	266	14.45	180
C	7823	78.23	78.23	294	11.68	203
H	7573	75.73	75.73	131	9.43	94.2

Institution:

Protocol: 202_00010650 513.PRO

Listmode Replay: New Protocol

Analysis Date: 03-May-2012, 13:00:43

Settings File: 00Ciclo Celular de Javi.PRO, 18-Apr-2012, 11:34:20

Listmode File: 30_00010642 505.LMD

Run Date: 02-May-12, 14:26:43

Sample ID: Well 30

User ID: anajavi

Acquisition Time/Events: 132.0s / 10000 (UNKNOWN)

Tube ID: NoRead

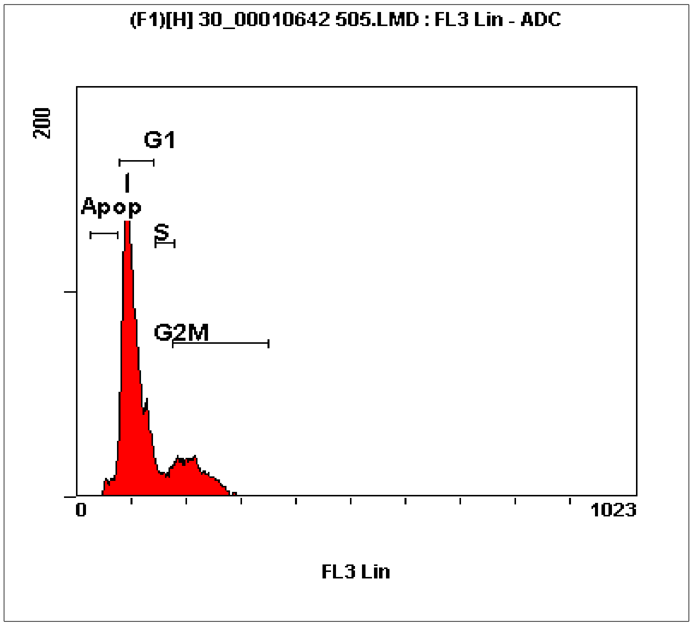

(F1)[H] 30_00010642 505.LMD : FL3 Lin

Region	Number	%Total	%Gated	X-Mean	HP X-CV	Y-Mea
ALL	7573	75.73	100.00	131	9.43	###
Apop	300	3.00	3.96	63.5	2.67	###
G1	5113	51.13	67.52	102	9.43	###
G2M	1620	16.20	21.39	222	0.66	###
S	478	4.78	6.31	161	0.65	###

Institution:
Protocol: 202_00010650 513.PRO
Listmode Replay: New Protocol
Analysis Date: 03-May-2012, 13:01:19
Settings File: 00Ciclo Celular de Javi.PRO, 18-Apr-2012, 11:34:20
Listmode File: 31_00010643 506.LMD

Run Date: 02-May-12, 14:29:32
Sample ID: Well 31
User ID: anajavi
Acquisition Time/Events: 86.3s / 10000 (UNKNOWN)

Instrument SN: AL01002 Software Version: CXP v2.2

(F1)[Ungated] 31_00010643 506.LMD : FS Lin/SS Lin - ADC

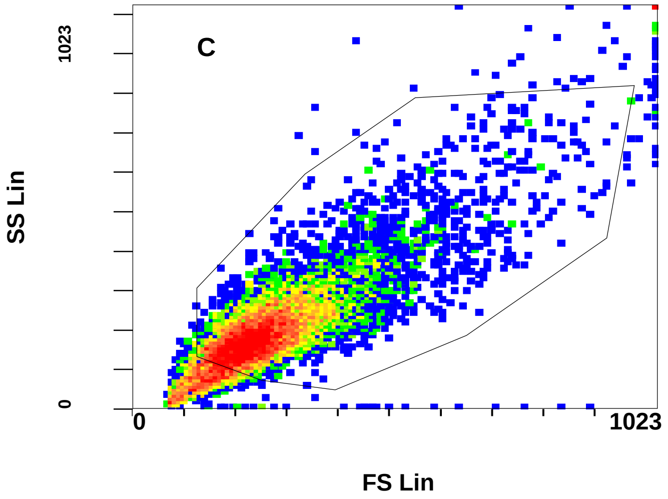

(F1)[Ungated] 31_00010643 506.LMD : FL3 Lin/AUX - ADC

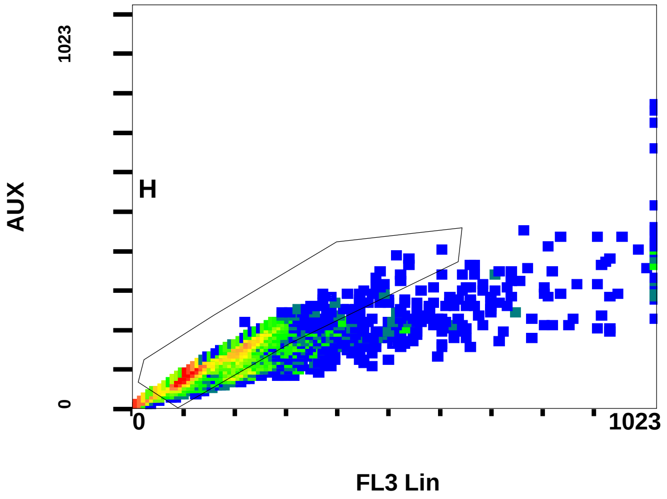

(F1)[H] 31_00010643 506.LMD : FL3 Lin - ADC

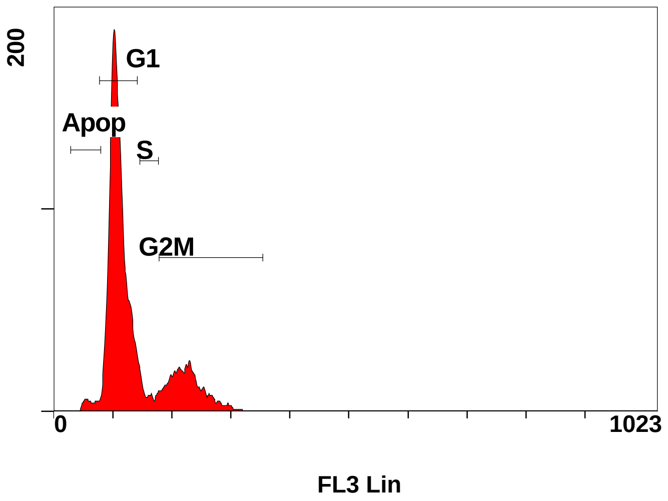

(F1)[H] 31_00010643 506.LMD : FL3 Lin/SS Lin - ADC

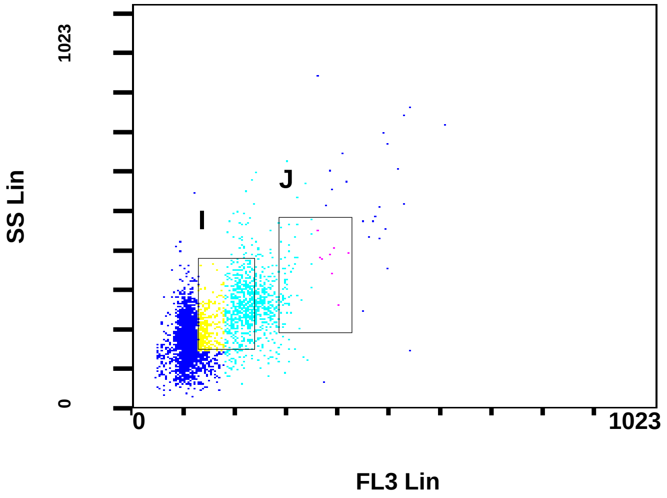

Statistical Analysis

PROGRAM INFORMATION

File:- 31_00010643 506.LMD						
Gate:- H [H]						
Compensation:- Advanced						
Filename:- 31_00010643 506.LMD						
Mean Calculation Method:-LOG-LOG						
Region	Number	%Total	%Gated	X-Mean	X-HPCV	Y-Mean
ALL	8024	80.24	100.00	140	9.37	###
ALL	8024	80.24	100.00	140	9.37	198
Apop	214	2.14	2.67	62.6	2.96	###
G1	5513	55.13	68.71	108	9.37	###
G2M	1822	18.22	22.71	231	1.02	###
I	1786	17.86	22.26	184	4.06	237
J	137	1.37	1.71	315	0.52	337
S	373	3.73	4.65	159	1.59	###

File:- 31_00010643 506.LMD						
Gate:- Ungated						
Compensation:- Advanced						
Filename:- 31_00010643 506.LMD						
Mean Calculation Method:-LOG-LOG						
Region	Number	%Total	%Gated	X-Mean	X-HPCV	Y-Mean
ALL	10000	100.00	100.00	270	16.55	203
ALL	10000	100.00	100.00	144	9.37	95.7
C	8371	83.71	83.71	287	14.28	219
H	8024	80.24	80.24	140	9.37	102

Institution:

Run Date: 02-May-12, 14:29:32

Protocol: 202_00010650 513.PRO

Sample ID: Well 31

Listmode Replay: New Protocol

User ID: anajavi

Analysis Date: 03-May-2012, 13:01:19

Acquisition Time/Events: 86.3s / 10000 (UNKNOWN)

Settings File: 00Ciclo Celular de Javi.PRO, 18-Apr-2012, 11:34:20

Tube ID: NoRead

Listmode File: 31_00010643 506.LMD

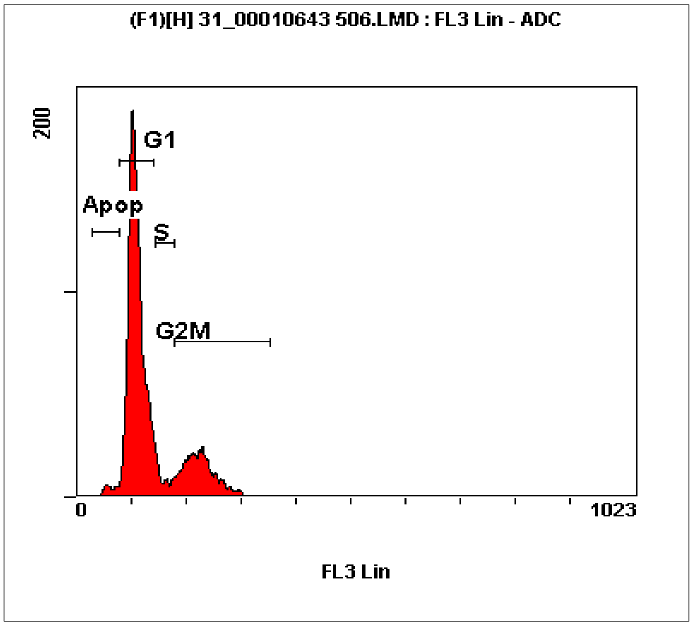

(F1)[H] 31_00010643 506.LMD : FL3 Lin						
Region	Number	%Total	%Gated	X-Mean	HP X-CV	Y-Mea
ALL	8024	80.24	100.00	140	9.37	###
Apop	214	2.14	2.67	62.6	2.96	###
G1	5513	55.13	68.71	108	9.37	###
G2M	1822	18.22	22.71	231	1.02	###
S	373	3.73	4.65	159	1.59	###

Institution:
Protocol: 202_00010650 513.PRO
Listmode Replay: New Protocol
Analysis Date: 03-May-2012, 13:01:55
Settings File: 00Ciclo Celular de Javi.PRO, 18-Apr-2012, 11:34:20
Listmode File: 33_00010647 510.LMD

Run Date: 02-May-12, 14:41:07
Sample ID: Well 33
User ID: anajavi
Acquisition Time/Events: 135.4s / 10000 (UNKNOWN)

Instrument SN: AL01002 Software Version: CXP v2.2

(F1)[Ungated] 33_00010647 510.LMD : FS Lin/SS Lin - ADC

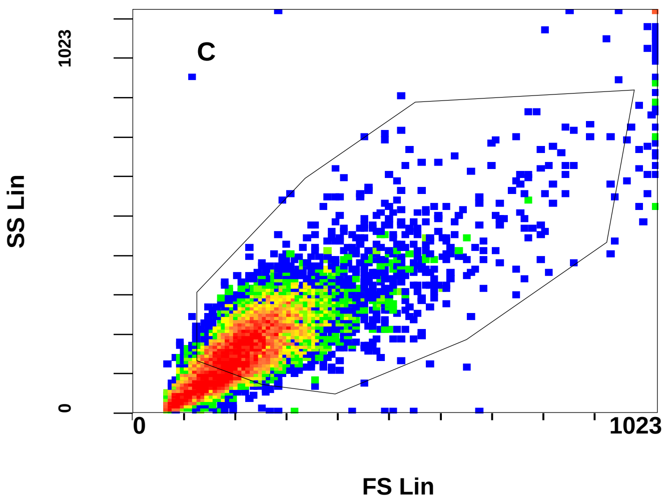

(F1)[Ungated] 33_00010647 510.LMD : FL3 Lin/AUX - ADC

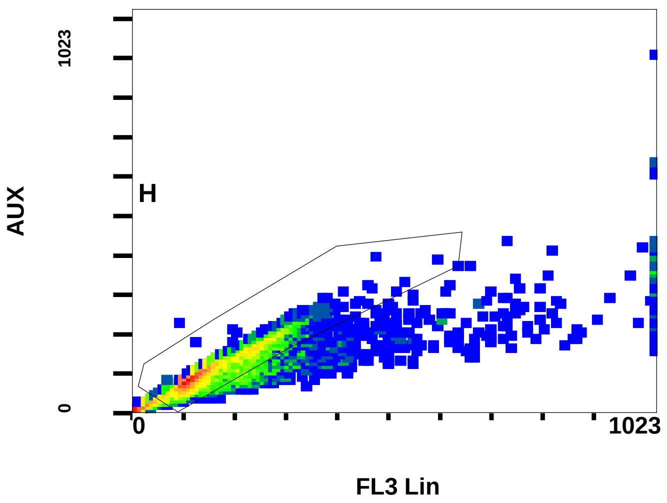

(F1)[H] 33_00010647 510.LMD : FL3 Lin - ADC

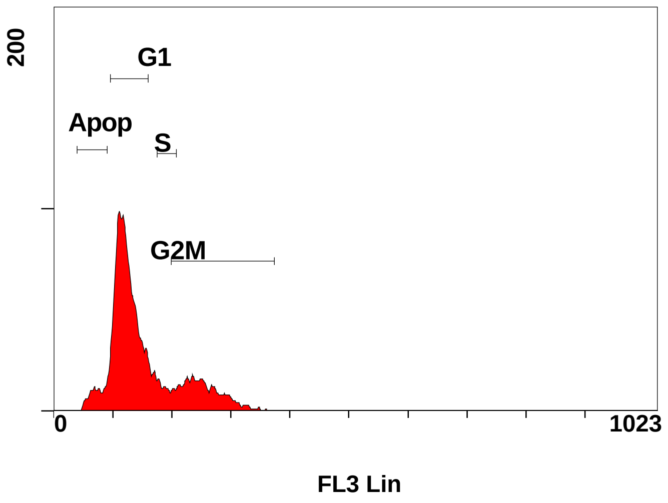

(F1)[H] 33_00010647 510.LMD : FL3 Lin/SS Lin - ADC

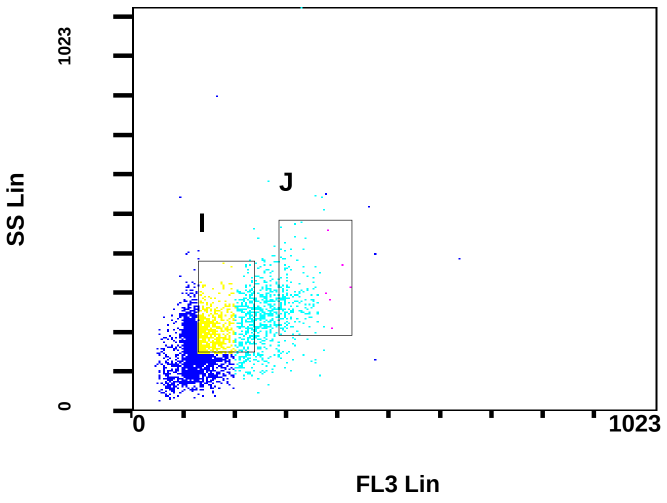

Statistical Analysis

PROGRAM INFORMATION

File:- 33_00010647 510.LMD						
Gate:- H [H]						
Compensation:- Advanced						
Filename:- 33_00010647 510.LMD						
Mean Calculation Method:-LOG-LOG						
Region	Number	%Total	%Gated	X-Mean	X-HPCV	Y-Mean
ALL	6886	68.86	100.00	158	10.71	###
ALL	6886	68.86	100.00	158	10.71	185
Apop	447	4.47	6.49	71.2	1.06	###
G1	4021	40.21	58.39	123	10.71	###
G2M	1655	16.55	24.03	258	2.49	###
I	1843	18.43	26.76	171	4.81	212
J	364	3.64	5.29	317	0.77	284
S	440	4.40	6.39	190	1.87	###

File:- 33_00010647 510.LMD						
Gate:- Ungated						
Compensation:- Advanced						
Filename:- 33_00010647 510.LMD						
Mean Calculation Method:-LOG-LOG						
Region	Number	%Total	%Gated	X-Mean	X-HPCV	Y-Mean
ALL	10000	100.00	100.00	141	517.09	88.4
ALL	10000	100.00	100.00	232	4.79	169
C	6982	69.82	69.82	266	2.85	205
H	6886	68.86	68.86	158	10.71	109

Institution:

Protocol: 202_00010650 513.PRO

Listmode Replay: New Protocol

Analysis Date: 03-May-2012, 13:01:56

Settings File: 00Ciclo Celular de Javi.PRO, 18-Apr-2012, 11:34:20

Listmode File: 33_00010647 510.LMD

Run Date: 02-May-12, 14:41:07

Sample ID: Well 33

User ID: anajavi

Acquisition Time/Events: 135.4s / 10000 (UNKNOWN)

Tube ID: NoRead

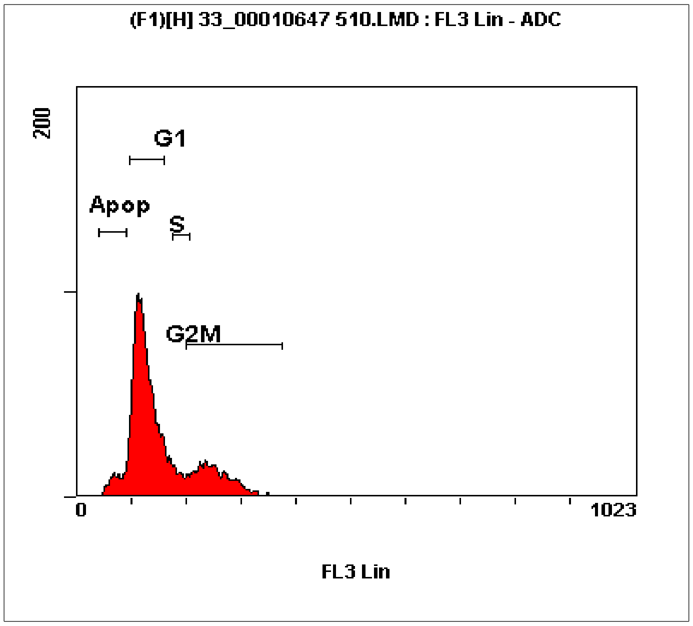

(F1)[H] 33_00010647 510.LMD : FL3 Lin

Region	Number	%Total	%Gated	X-Mean	HP X-CV	Y-Mea
ALL	6886	68.86	100.00	158	10.71	###
Apop	447	4.47	6.49	71.2	1.06	###
G1	4021	40.21	58.39	123	10.71	###
G2M	1655	16.55	24.03	258	2.49	###
S	440	4.40	6.39	190	1.87	###

Institution:

Protocol: 202_00010650 513.PRO

Listmode Replay: New Protocol

Analysis Date: 03-May-2012, 13:02:36

Settings File: 00Ciclo Celular de Javi.PRO, 18-Apr-2012, 11:34:20

Listmode File: 34_00010648 511.LMD

Run Date: 02-May-12, 14:43:58

Sample ID: Well 34

User ID: anajavi

Acquisition Time/Events: 146.0s / 10000 (UNKNOWN)

Instrument SN: AL01002 Software Version: CXP v2.2

(F1)[Ungated] 34_00010648 511.LMD : FS Lin/SS Lin - ADC

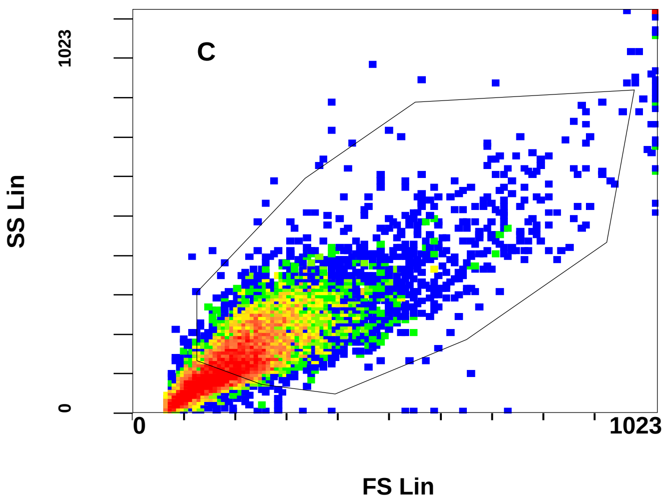

(F1)[Ungated] 34_00010648 511.LMD : FL3 Lin/AUX - ADC

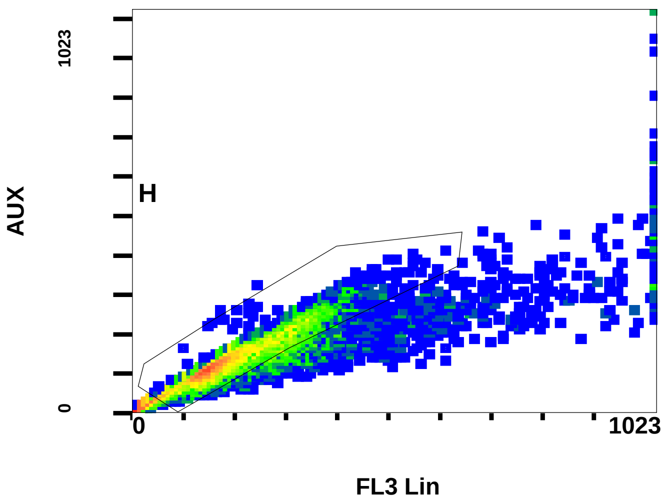

(F1)[H] 34_00010648 511.LMD : FL3 Lin - ADC

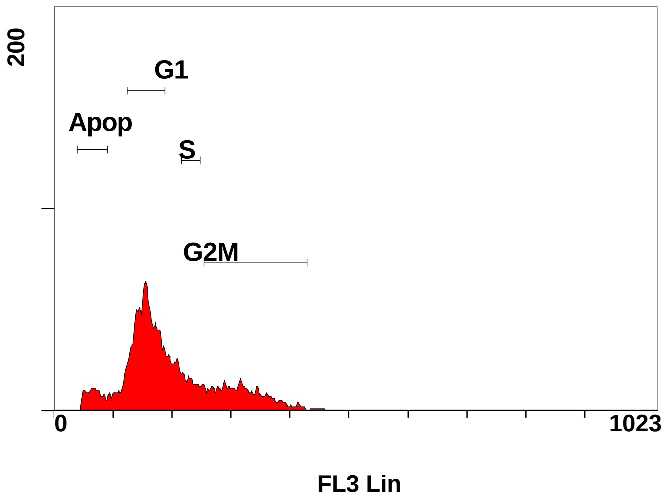

(F1)[H] 34_00010648 511.LMD : FL3 Lin/SS Lin - ADC

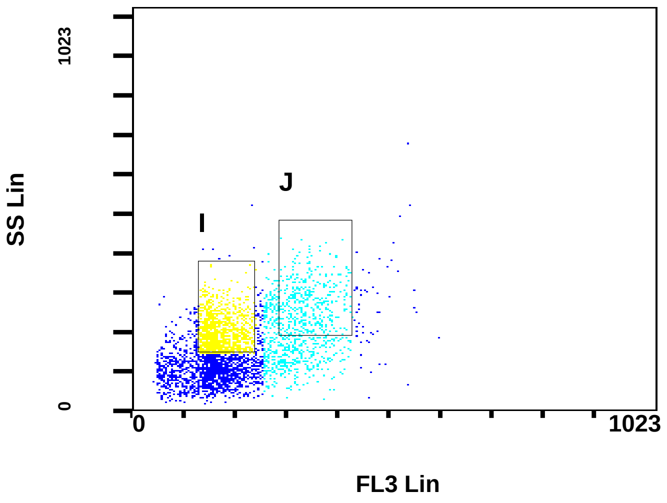

Statistical Analysis

PROGRAM INFORMATION

File:- 34_00010648 511.LMD						
Gate:- H [H]						
Compensation:- Advanced						
Filename:- 34_00010648 511.LMD						
Mean Calculation Method:-LOG-LOG						
Region	Number	%Total	%Gated	X-Mean	X-HPCV	Y-Mean
ALL	6937	69.37	100.00	205	8.45	###
ALL	6937	69.37	100.00	205	8.45	168
Apop	469	4.69	6.76	66.9	1.72	###
G1	2929	29.29	42.22	156	8.45	###
G2M	1693	16.93	24.41	323	0.50	###
I	2028	20.28	29.23	172	7.21	206
J	819	8.19	11.81	343	0.22	279
S	550	5.50	7.93	231	0.93	###

File:- 34_00010648 511.LMD						
Gate:- Ungated						
Compensation:- Advanced						
Filename:- 34_00010648 511.LMD						
Mean Calculation Method:-LOG-LOG						
Region	Number	%Total	%Gated	X-Mean	X-HPCV	Y-Mean
ALL	10000	100.00	100.00	198	396.16	123
ALL	10000	100.00	100.00	237	4.00	164
C	6532	65.32	65.32	284	4.02	206
H	6937	69.37	69.37	205	8.45	142

Institution:

Protocol: 202_00010650 513.PRO

Listmode Replay: New Protocol

Analysis Date: 03-May-2012, 13:02:36

Settings File: 00Ciclo Celular de Javi.PRO, 18-Apr-2012, 11:34:20

Listmode File: 34_00010648 511.LMD

Run Date: 02-May-12, 14:43:58

Sample ID: Well 34

User ID: anajavi

Acquisition Time/Events: 146.0s / 10000 (UNKNOWN)

Tube ID: NoRead

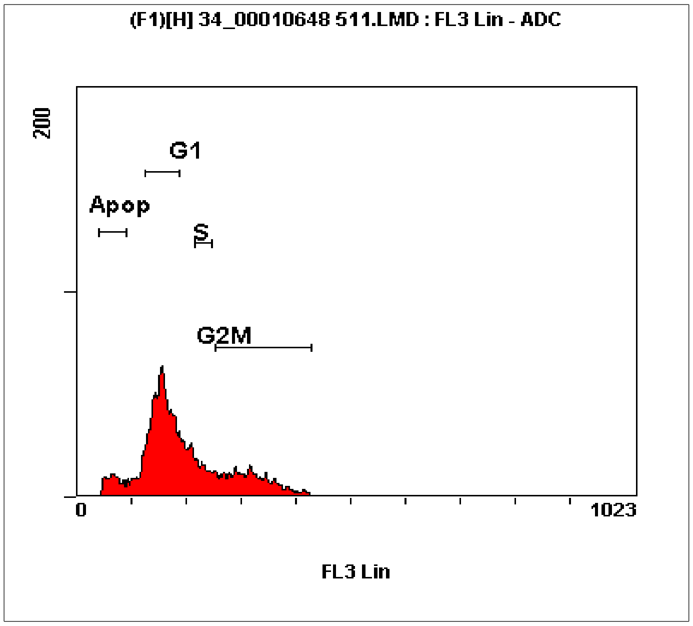

(F1)[H] 34_00010648 511.LMD : FL3 Lin

Region	Number	%Total	%Gated	X-Mean	HP X-CV	Y-Mea
ALL	6937	69.37	100.00	205	8.45	###
Apop	469	4.69	6.76	66.9	1.72	###
G1	2929	29.29	42.22	156	8.45	###
G2M	1693	16.93	24.41	323	0.50	###
S	550	5.50	7.93	231	0.93	###

Institution:
Protocol: 01_00010606 476.PRO
Listmode Replay: New Protocol
Analysis Date: 03-May-2012, 12:36:36
Settings File: 00Ciclo Celular de Javi.PRO, 18-Apr-2012, 11:34:20
Listmode File: 03_00010635 498.LMD

Run Date: 02-May-12, 14:01:20
Sample ID: Well 01
User ID: anajavi
Acquisition Time/Events: 93.8s / 10000 (UNKNOWN)

Instrument SN: AL01002 Software Version: CXP v2.2

(F1)[Ungated] 03_00010635 498.LMD : FS Lin/SS Lin - ADC

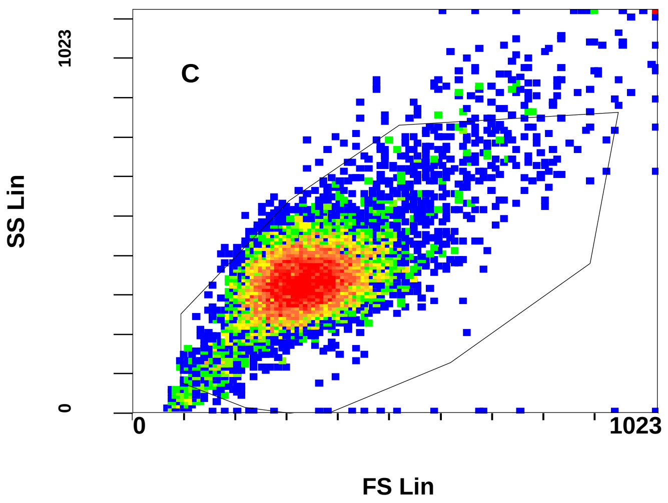

(F1)[Ungated] 03_00010635 498.LMD : FL3 Lin/AUX - ADC

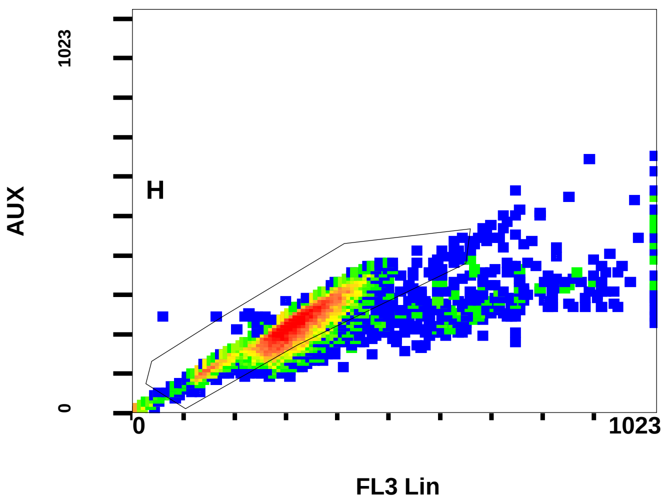

(F1)[H] 03_00010635 498.LMD : FL3 Lin - ADC

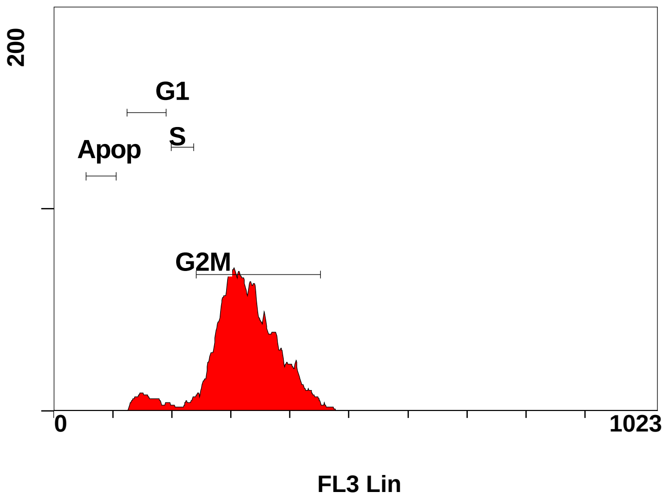

(F1)[H] 03_00010635 498.LMD : FL3 Lin/SS Lin - ADC

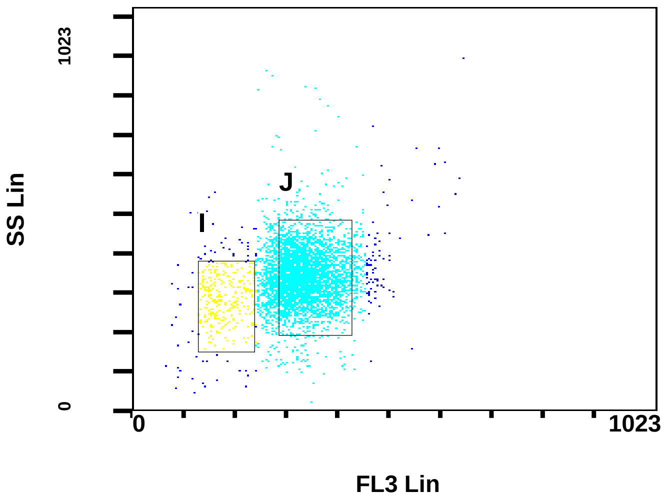

Statistical Analysis

PROGRAM INFORMATION

File:- 03_00010635 498.LMD

Gate:- H [H]

Compensation:- Advanced

Filename:- 03_00010635 498.LMD

Mean Calculation Method:-LOG-LOG

Region	Number	%Total	%Gated	X-Mean	X-HPCV	Y-Mean
ALL	8956	89.56	100.00	321	9.23	###
ALL	8956	89.56	100.00	321	9.23	340
Apop	22	0.22	0.25	84.5	1.07	###
G1	488	4.88	5.45	156	1.16	###
G2M	8029	80.29	89.65	332	9.23	###
I	615	6.15	6.87	177	0.80	285
J	6258	62.58	69.87	340	8.59	341
S	191	1.91	2.13	221	0.33	###

File:- 03_00010635 498.LMD

Gate:- Ungated

Compensation:- Advanced

Filename:- 03_00010635 498.LMD

Mean Calculation Method:-LOG-LOG

Region	Number	%Total	%Gated	X-Mean	X-HPCV	Y-Mean
ALL	10000	100.00	100.00	352	6.00	347
ALL	10000	100.00	100.00	328	8.32	220
C	9721	97.21	97.21	348	6.00	342
H	8956	89.56	89.56	321	9.23	225

Institution:

Protocol: 01_00010606 476.PRO

Listmode Replay: New Protocol

Analysis Date: 03-May-2012, 12:36:36

Settings File: 00Ciclo Celular de Javi.PRO, 18-Apr-2012, 11:34:20

Listmode File: 03_00010635 498.LMD

Run Date: 02-May-12, 14:01:20

Sample ID: Well 01

User ID: anajavi

Acquisition Time/Events: 93.8s / 10000 (UNKNOWN)

Tube ID: NoRead

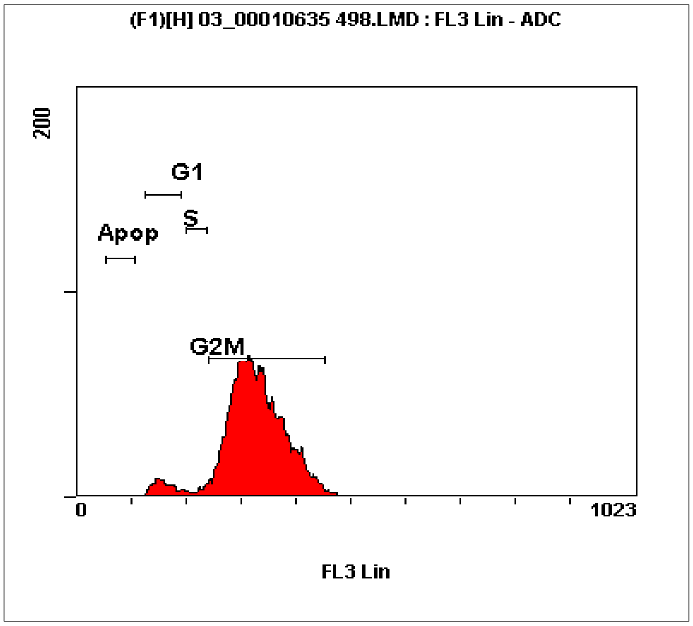

(F1)[H] 03_00010635 498.LMD : FL3 Lin

Region	Number	%Total	%Gated	X-Mean	HP X-CV	Y-Mea
ALL	8956	89.56	100.00	321	9.23	###
Apop	22	0.22	0.25	84.5	1.07	###
G1	488	4.88	5.45	156	1.16	###
G2M	8029	80.29	89.65	332	9.23	###
S	191	1.91	2.13	221	0.33	###

Institution:
Protocol: 01_00010606 476.PRO
Listmode Replay: New Protocol
Analysis Date: 03-May-2012, 12:37:05
Settings File: 00Ciclo Celular de Javi.PRO, 18-Apr-2012, 11:34:20
Listmode File: 04_00010609 479.LMD

Run Date: 02-May-12, 12:48:14
Sample ID: Well 04
User ID: anajavi
Acquisition Time/Events: 98.6s / 10000 (UNKNOWN)

Instrument SN: AL01002 Software Version: CXP v2.2

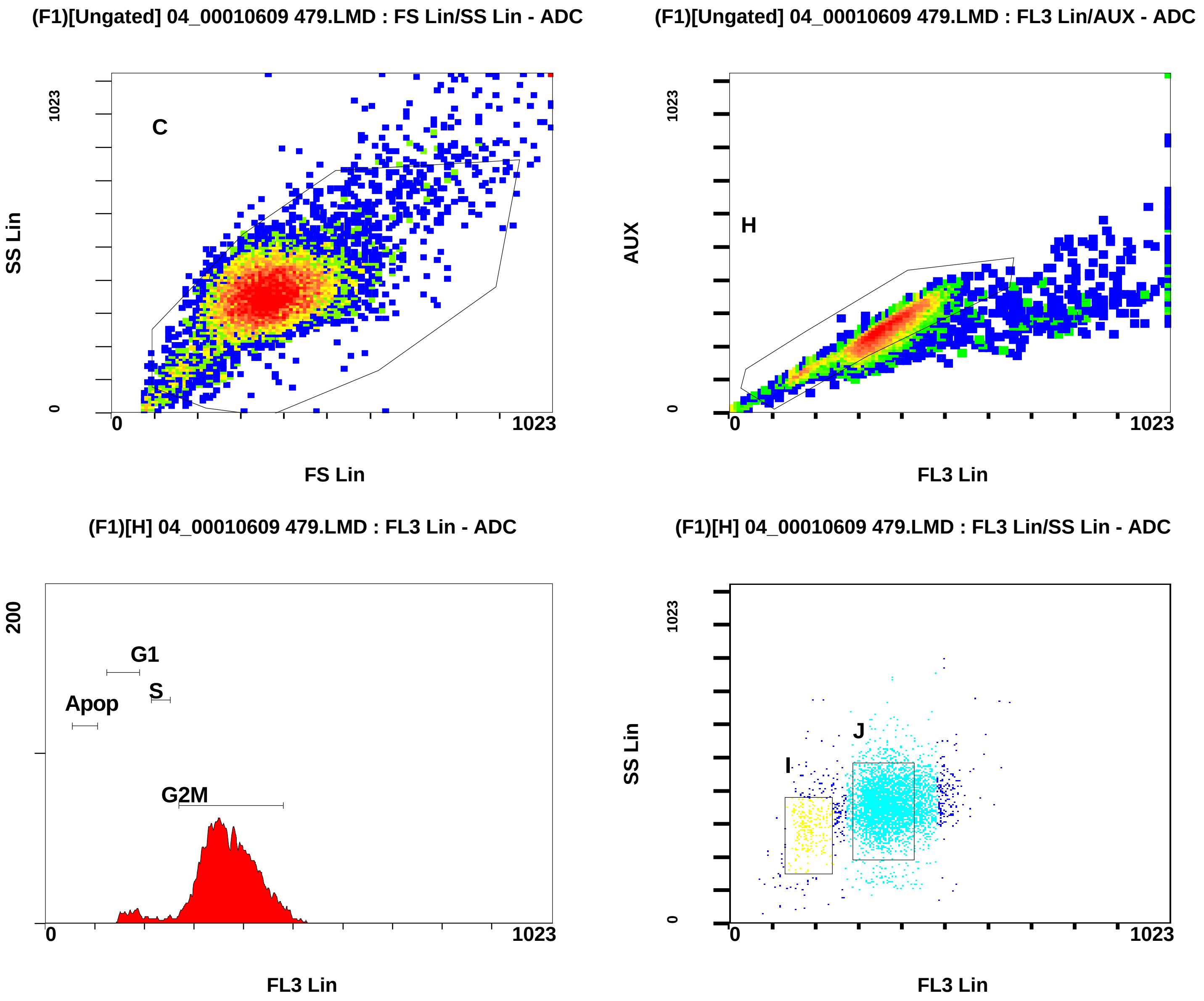

Statistical Analysis

PROGRAM INFORMATION

File:- 04_00010609 479.LMD						
Gate:- H [H]						
Compensation:- Advanced						
Filename:- 04_00010609 479.LMD						
Mean Calculation Method:-LOG-LOG						
Region	Number	%Total	%Gated	X-Mean	X-HPCV	Y-Mean
ALL	8929	89.29	100.00	360	11.93	###
ALL	8929	89.29	100.00	360	11.93	357
Apop	21	0.21	0.24	87.7	0.72	###
G1	388	3.88	4.35	168	1.87	###
G2M	7828	78.28	87.67	371	11.93	###
I	491	4.91	5.50	185	0.92	287
J	6171	61.71	69.11	360	11.95	357
S	175	1.75	1.96	233	0.26	###

File:- 04_00010609 479.LMD						
Gate:- Ungated						
Compensation:- Advanced						
Filename:- 04_00010609 479.LMD						
Mean Calculation Method:-LOG-LOG						
Region	Number	%Total	%Gated	X-Mean	X-HPCV	Y-Mean
ALL	10000	100.00	100.00	375	7.74	363
ALL	10000	100.00	100.00	367	11.93	244
C	9704	97.04	97.04	372	7.74	358
H	8929	89.29	89.29	360	11.93	250

Institution:

Protocol: 01_00010606 476.PRO

Listmode Replay: New Protocol

Analysis Date: 03-May-2012, 12:37:05

Settings File: 00Ciclo Celular de Javi.PRO, 18-Apr-2012, 11:34:20

Listmode File: 04_00010609 479.LMD

Run Date: 02-May-12, 12:48:14

Sample ID: Well 04

User ID: anajavi

Acquisition Time/Events: 98.6s / 10000 (UNKNOWN)

Tube ID: NoRead

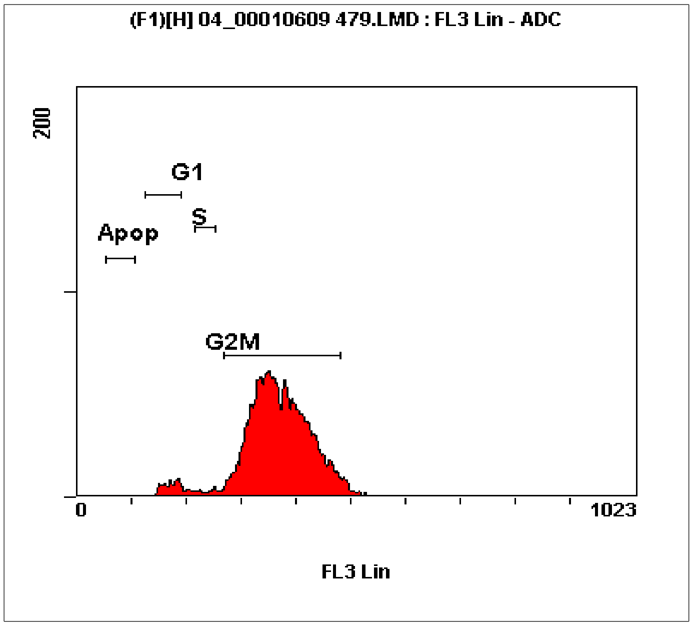

(F1)[H] 04_00010609 479.LMD : FL3 Lin

Region	Number	%Total	%Gated	X-Mean	HP X-CV	Y-Mea
ALL	8929	89.29	100.00	360	11.93	###
Apop	21	0.21	0.24	87.7	0.72	###
G1	388	3.88	4.35	168	1.87	###
G2M	7828	78.28	87.67	371	11.93	###
S	175	1.75	1.96	233	0.26	###
